# Synergistic Degradation of Durable Polymer Networks by Light and Acid Enabled by Pyrenylsilicon Crosslinks

**DOI:** 10.1002/adma.202412544

**Published:** 2024-12-04

**Authors:** Yutaro Kawano, Hiroshi Masai, Takuya Tsubokawa, Daisuke Yokogawa, Tomohiro Iwai, Jun Terao

**Affiliations:** ^1^ Department of Basic Science Graduate School of Arts and Sciences The University of Tokyo 3‐8‐1, Komaba, Meguro‐ku Tokyo 153‐8902 Japan; ^2^ PRESTO Japan Science and Technology Agency 4‐1‐8, Honcho, Kawaguchi Saitama 332‐0012 Japan

**Keywords:** 3D‐lithography, luminescence, microprocessing, photodegradation, soft materials

## Abstract

Material photocontrol has gained importance in process engineering and biomedical applications. However, highly photoreactive materials are intrinsically unstable to light, which limits their continuous use in lit environments owing to their gradual deterioration. Herein, synergistically photocontrollable materials in the presence of acid are developed to overcome the conventional trade‐off between their photoreactivity and photostability. Pyrenylsilicon derivatives are designed as synergistically cleavable moieties on C–Si bonds under simultaneous treatment with light and acid through photoinduced dearomatization and protonation to generate the Wheland intermediate, whereas the derivatives are highly stable to light or acid alone. The unique reactivity of pyrenylsilicon derivatives is applied to various polymer network crosslinkers, enabling synergistic control and degradation of materials with light and acids. Because of their high photostability in the absence of acids, these materials can be utilized as optical materials, robust elastomers, and 3D photoprinted gels.

## Graphical abstract

A significant advancement in resolving the incompatibility between photodegradablity and photostability of polymer materials is reported by developing a novel cleavage reaction of the C–Si bond under simultaneous exposure to light and acid. The synergistic activation of polymer materials can be applied to a wide range of materials, including color‐tunable optical materials, tough elastic materials, and additive/subtractive 3D‐photoprinting materials.

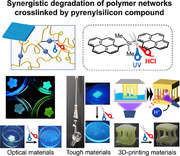

## 1 Introduction

**Figure 1:**
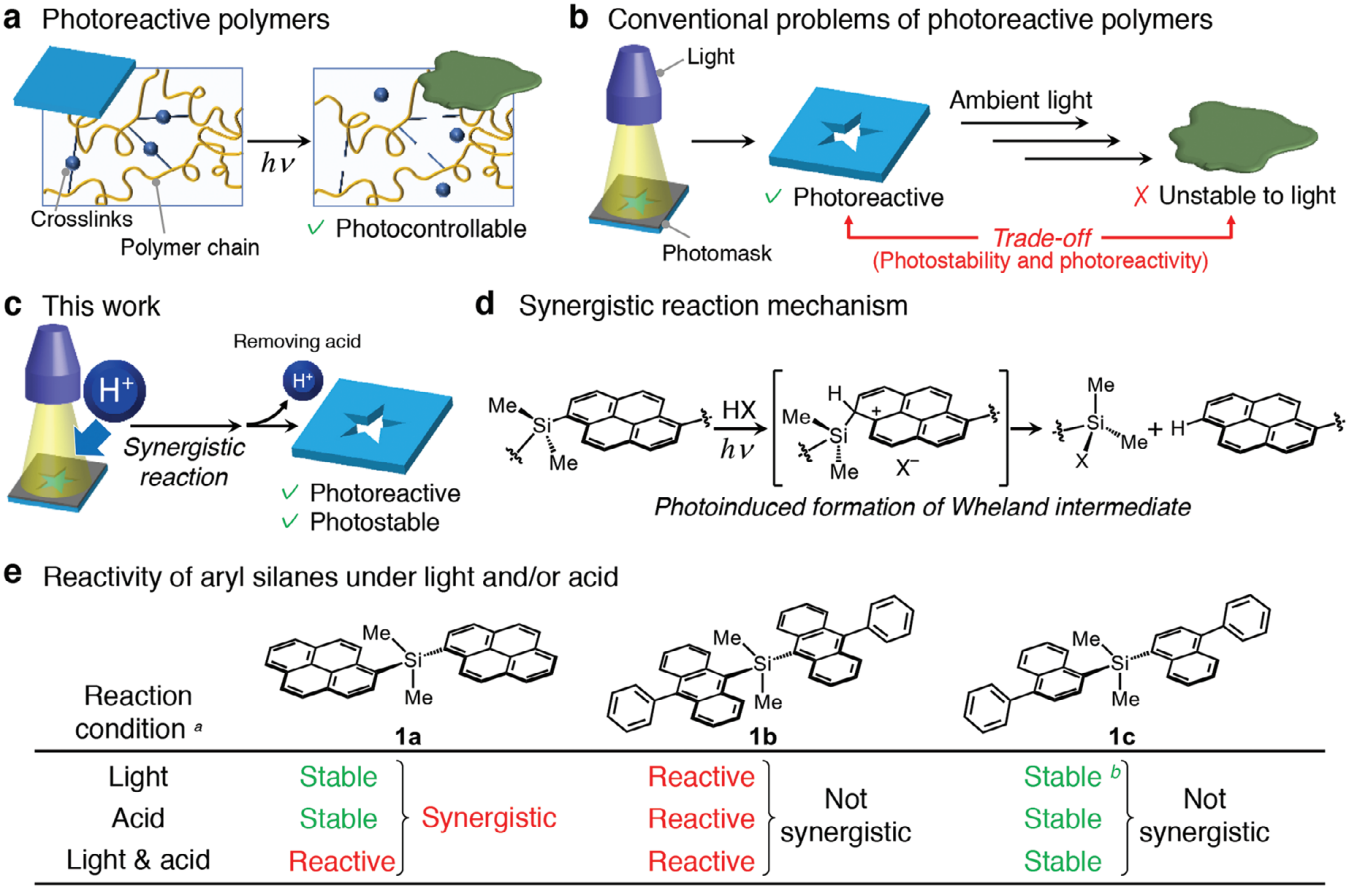
Conceptual illustrations of a) photoreactive polymer materials with simple reactivity to light, b) conventional trade‐off relationship between photoprocessability and photostability in typical photoreactive polymers, and c) synergistically reactive materials under simultaneous stimulation with light and acid (this work). d) Proposed reaction mechanism and key intermediate of synergistic cleavage reaction with light (*hν*) and acid (HX) on pyrenylsilicon derivative. e) Summary of reactivities of aryl silanes (1a–1c) under light and/or acid. *^a^* 1 (1.0 µmol) in THF/EtOAc (5/3, v/v) was stirred under 365 nm UV light (0.3 W·cm^−2^) and/or HCl (1.5 m) at room temperature for 30 min. *^b^* 1c has no absorption at 365 nm.

Photoreactive polymer materials have gained importance in various fields such as photoresists,^[^ ^1^, ^2^ ^]^ drug delivery systems,^[^ ^3^, ^4^ ^]^ cell cultures,^[^ ^5^, ^6^ ^]^ and soft actuators.^[^ ^7^, ^8^ ^]^ The shapes and properties of polymeric materials can be remotely and precisely manipulated via photochemical processing.^[^ ^9^, ^10^, ^11^ ^]^ In particular, the effective photocleavage of cross‐linking points in the polymer network enables rapid tuning and degradation of the materials (**Figure** **1**a).^[^ ^12^, ^13^, ^14^ ^]^ However, highly photoreactive materials are intrinsically unstable to light, limiting their continuous usage in a lit environment owing to their gradual deterioration (Figure 1b). Namely, efficient photoreactivity and long‐term photostability of materials have a trade‐off relationship, resulting in the restricted usage of conventional photoreactive materials in dark environments and temporal utilization. Accordingly, overcoming the tradeoff between photoreactivity and photostability would allow photoreactive material applications to be utilized under ambient light or in photoexcited environments before and after photoprocessing and to expand to diverse fields, including optical materials, material photofabrication, and long‐term structural materials.

To address this trade‐off, synergistic reactivity with multiple stimuli such as light and chemical reagents is a promising strategy. Such materials remain intact when exposed to a single stimulus (light *or* chemicals), whereas they are highly reactive to simultaneous treatment with multiple stimuli (light *and* chemicals) (Figure 1c). The materials’ photoreactivity is completely switchable by the addition or removal of an extra stimulus, leading to compatibility between the long‐term photostability and photoreactivity of the materials. Recently, sequential‐stimuli‐responsive polymers have been developed by incorporating multiple types of stimuli‐responsive units or stepwise responsive moieties into polymer network materials, providing advanced material reactivity with stimuli of light and chemicals above simple photodegradation.^[^ ^15^, ^16^, ^17^, ^18^, ^19^, ^20^, ^21^ ^]^ However, such sequential reactivities inevitably induce irreversible changes in the materials in ambient light or photoexcited environments, resulting in a loss of long‐term photostability. Therefore, the synergistic activation of materials is required to overcome the materials’ trade‐off between efficient photoreactivity and long‐term photostability.

We recently developed an acid‐induced photoreactive material incorporated with a supramolecular platinum acetylide complex as a synergistically reactive crosslinker, which was photocleavable in the presence of an acid but photostable in its absence.^[^ ^22^, ^23^ ^]^ The material achieved switching between photostability and photoreactivity, resolving the trade‐off to realize unprecedented phototechnologies, including the photoprocessing of photoluminescent and photopolymerized materials. However, the synergistic reactivity was derived from the specific supramolecular structure incorporating the precious metal Pt and required scarce chemicals and a laborious synthetic route. In addition, susceptible metal–carbon bonds would result in materials that are relatively fragile to various stimuli, including strong acid or UV light. These features hinder diverse applications of synergistically reactive materials owing to the durability and availability of the reactive moiety.

In this study, a novel methodology for the synergistically triggered activation of polymeric materials was developed using a newly designed pyrenylsilicon compound with a ubiquitous chemical structure in which the robust C–Si bond was synergistically photocleavable with acids (Figure 1d). The photoinduced protonation‐desilylation of arylsilanes has been fundamentally investigated for phenylsilane and anthracenylsilane derivatives.^[^ ^24^, ^25^, ^26^, ^27^ ^]^ Although the typical α‐protonation of arylsilanes requires harsh reaction conditions because of the formation of a dearomatic intermediate (Wheland intermediate) for protonation, the photoexcitation of the aromatic rings decreases the multiplicity of the chemical bonds (bond order) to induce dearomatization,^[^ ^28^, ^29^, ^30^ ^]^ leading to accelerated protonation and subsequent desilylation compared to the ground state.

Herein, we further developed the photoinduced reactivity of arylsilanes, directed toward synergistically reactive polymer materials, to achieve an optimal balance between high photostability and photoreactivity. As a preliminary experiment, the synergistic reactivities of arylsilanes (1a–1c) were evaluated using 365‐nm UV light and HCl (Figure 1e), as low‐influential stimuli on materials that are easy to apply and remove. Consequently, the pyrenylsilicon derivative (1a) possessed the best synergistic reactivity and was degraded under cooperative stimulation with light and acid, although it was stable under light or acid alone (Figure S4, Supporting Information). In contrast, anthracenylsilane (1b) was unstable even under a single stimulus, whereas naphthylsilane (1c) was stable under cooperative stimulation (Figures S5 and S6, Supporting Information). The compatibility of the high stability under acidic conditions and high absorption efficiency of the pyrenylsilicon moiety at 365 nm enabled excellent synergistic reactivity for C–Si bond cleavage. This study applied the unique reactivity of pyrenylsilicon derivatives to synergistically controllable materials with light and acid to overcome the tradeoff between photoreactivity and photostability through various hierarchical investigations, from nanoscale molecular reactions to macroscale material responsiveness.

## 2 Results and Discussions

### 2.1 Design of Synergistic Light–Acid Reactivity of Pyrenylsilicon Compound

**Figure 2:**
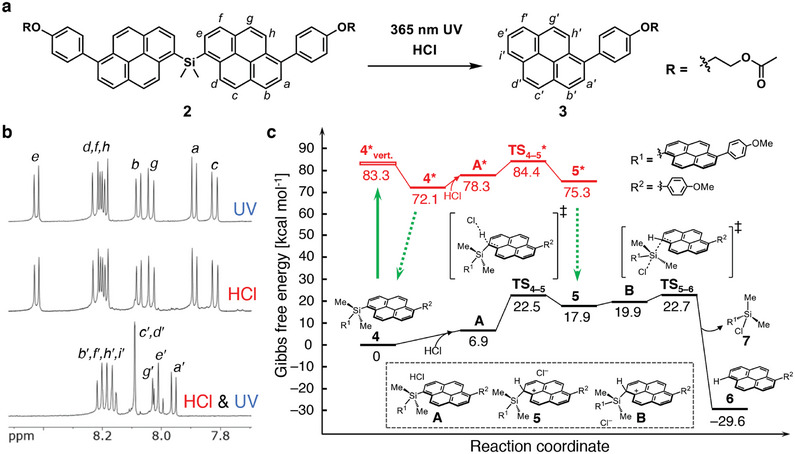
a) Scheme for degradation of **2** under simultaneous stimulation with light and acid. b) ^1^H NMR spectra (500 MHz, CDCl_3_, r.t.) recorded after reaction of **2** under three different arrangements of stimuli for 30 min (UV:  365 nm, 0.3 W·cm^−2^; HCl: 0.5 m HCl). c) Energy profile for desilylation of **4** with HCl in ground and excited states (CAM‐B3LYP/cc‐pVDZ (for H, C, Si); aug‐cc‐pVDZ (for O, Cl), SMD (EtOAc)). Green rigid arrow: photoexcitation; green dotted arrows: radiative or non‐radiative processes.

To exploit the pyrenylsilicon moieties as crosslinkers in the polymer networks, two phenyl ether tethers were introduced at the 6‐positions of the 1‐pyrenylsilicon derivative (**Figure** **2**a). The dipyrenyldimethylsilane moiety was prepared via halogen–lithium exchange of 1‐bromopyrene derivatives, followed by trapping with dichlorodimethylsilane (See Supporting Information).^[^ ^31^, ^32^ ^]^ The resultant acetyl‐terminated di(6‐phenylpyren‐1‐yl)dimethylsilane derivative (2) exhibited a strong absorption band at 320–400 nm (Figure S48, Supporting Information). As confirmed by the post‐reaction SEC chromatograms, 2 underwent a quantitative C–Si bond cleavage under the cooperative stimulation of acid (0.5 m HCl) and light (365 nm, 0.3 W·cm^−2^) (Figure S9, Supporting Information). In addition, the effects of HCl concentrations, intensities of UV light, and wavelengths of light exposure were investigated. The synergistic photocleavage was accelerated as HCl concentrations or UV light intensities increased (Figure S18a,b, Supporting Information). Furthermore, the synergistic cleavage was efficiently promoted under light at a wavelength below 400 nm, which was consistent with the absorption area of pyrenylsilane (Figures S18c and S48, Supporting Information). Based on further investigation by ^1^H NMR analysis, 3 was obtained in >95% yield after cooperative stimulation of acid (0.5 m HCl) and light (365 nm, 0.3 W·cm^−2^) (Figure 2b). Notably, under UV light (up to 0.8 W·cm^−2^) or HCl (0.5 m) alone, 2 remained unconverted, as confirmed by the ^1^H NMR spectra. The high robustness of the pyrenylsilicon derivative under a single stimulus of UV light or HCl contrasts the platinum complex in a previous study. Although the platinum complex was stable to weak UV light (0.008 W·cm^−2^) or HCl (0.2 m) alone,^[^ ^22^ ^]^ it was degradable even under light (0.3 W·cm^−2^) or acidic (0.5 m) conditions, which were stable conditions for pyrenylsilane (Figure S8, Supporting Information). Namely, the photostability of the silicon compound was ≈100 folds greater than that of platinum while showing excellent synergistic reactivity.

The synergistic reaction of **2** proceeds efficiently under either an aerobic or anaerobic atmosphere (Figure S13, Supporting Information). Similarly, the photocleavage of **2** with acid proceeded even in the presence of the 2,2,6,6‐tetramethylpiperidine‐1‐oxyl (TEMPO) radical as a radical scavenger and the trapped compound was not observed with or without acid (Figures S14 and S15, Supporting Information). Hence, the synergistic reaction proceeds through the photoexcited singlet species to react with HCl rather than with the triplet or radical species; this was in contrast to acid‐induced photocleavage reaction of the platinum complex.^[^ ^22^, ^23^ ^]^ A plausible reaction mechanism was revealed using density functional theory (DFT) calculations for the model reaction of the pyrenylsilicon derivative (**4**) with HCl in the ground (S_0_) and excited (S_1_) states (Figure 2c and Supporting Information). In the excited state, 4* is protonated with HCl through the transition state (TS_4–5_*) to yield the excited Wheland intermediate (5*), affording **6** via desilylation in the ground state to regain aromatic stability. The Gibbs free energy of the transition state for protonation in the excited state (Δ*G* * ^o‡^ **_4–5_**) was calculated as 12.3 kcal·mol^−1^, which is significantly lower than that in the ground state (Δ*G* ^o‡^ **_4–5_** = 22.5 kcal·mol^−1^). Moreover, the Gibbs free energies of protonation from **4** to **5** in the excited state were more favorable than that in the ground state (Δ*G* *°**_4–5_** = 3.2 kcal·mol^−1^ versus Δ*G*°**_4–5_** = 17.3 kcal·mol^−1^). The lower energies of activation (Δ*G* ^o‡^ **_4–5_**) and formation (Δ*G*°**_4–5_**) in the excited pathway were attributed to the dearomatization in the photoexcited substrate (4*), as a preparatory state of the protonated intermediate (5*). Accordingly, the excited‐state pathway decreased the activation barrier and endotherm in the protonation of **4** as compared to the ground‐state pathway, accelerating the formation of the Wheland intermediate, which contributed to the synergistic light–acid reactivity.

### 2.2 Synergistic Controlling of Polymeric Materials Bearing Dipyrenylsilane‐Based Crosslinker

**Figure 3:**
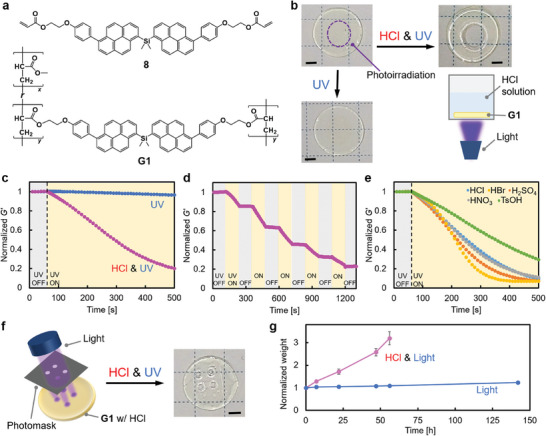
a) Chemical structure of dipyrenylsilicon crosslinker (8) and polymer network crosslinked with 8 (G1, x/y = 100/0.09). b) Photographs and schematic of synergistic photoprocessing of G1 before and after exposure to 365‐nm UV light (50 mW·cm^−2^) on center of gel with or without HCl (0.5 m). c) Evolution of normalized storage modulus *G′* of G1 during photo‐irradiation with (pink) or without (blue) HCl. d) Time‐course of *G′* during periodic ON‐OFF experiment of UV irradiation in presence of HCl. e) Evolution of normalized *G′* during processing of G1 under various acidic reagents with UV light irradiation. As standard conditions, 365‐nm UV light (2 mW·cm^−2^) and acidic reagents (0.2 m) were utilized. f) Photograph and illustration of G1 before and after exposure to 365‐nm UV (80 mW·cm^−2^) light with HCl (0.5 m) through a dot‐patterned photomask. g) Time‐course of normalized weight of processed gels (fabricated in (f)) under exposure to fluorescent light (20 W) in absence (blue) and presence (pink) of HCl (0.2 m) in DMF/EtOAc (19/1, v/v). Scale bar: 2 mm.

Synergistic reactivity was applied to the macroscopic control of polymeric materials by incorporating a dipyrenylsilicon derivative as a crosslinker in the polymer networks. Free‐radical chain copolymerization of methyl acrylate (1 equiv.) with a pyrenylsilane‐based crosslinker (**8**, 0.0009 equiv.) in the presence of a radical initiator yielded a polymer network that retained the DMF solvent to form a transparent and elastic gel (**G1**; **Figure** **3**a). Moreover, the crosslinker (**8**) could be incorporated into various types of polymer networks composed of polyacrylate, polyacrylamide, and polymethacrylate via copolymerization, providing diverse gels containing the synergistic reactive moiety in common polymers (**G8**–**G10**; Figure S2, Supporting Information). The macroscopic changes in shape were investigated to elucidate the synergistic light–acid responsiveness of **G1**. As a standard procedure, cooperative stimuli were provided to the disk‐shaped **G1** under 365‐nm UV light irradiation after introducing HCl via solvent replacement in the gel (Figure 3a, bottom). The cooperative stimuli induced local degradation of the photoirradiated area via a solation due to the synergistic cleavage of the pyrenylsilane‐based crosslinking points (Figure 3b). Notably, a small amount of the pyrenylsilicon moiety (below 1/1000 equivalent to the monomer component) induced macroscopic responsiveness via synergistic reactivity. In contrast, **G1** remained intact under photoirradiation in the absence of HCl. Furthermore, the application of cooperative stimuli in **G1** induced a twofold increase in the swelling degree of the gel because the cleavage of crosslinkers in polymer networks induced to increase osmotic pressure,^[^ ^33^ ^]^ whereas the swelling ratio was constant under treatment with light or acid alone (Figure S24, Supporting Information). The pyrenylsilane‐crosslinked material exhibited synergistic photodegradability with acid, whereas it was photostable under acid‐free conditions.

Quantitative rheological analyses were conducted for the disk‐shaped **G1** to clarify the time course of viscoelasticity under light irradiation in the presence of HCl. Because the storage modulus (*G*′) of network materials is proportional to the crosslinking density, the synergistic reactivity of pyrenylsilane‐based crosslinkers was confirmed by decreasing *G*′ of **G1** during photoirradiation in the presence of HCl (Figure 3c). The same reactivity was confirmed for various polymer networks including polyacrylate, polyacrylamide, and polymethacrylate (Figure S31, Supporting Information). In contrast, without HCl, the *G*′ was constant during the photoirradiation, demonstrating the drastic switching of the materials’ photoreactivity between acidic and non‐acidic conditions. The periodic ON‐OFF experiment of photoirradiation revealed that the decrease of *G*′ in **G1** was attributed to the light stimulus (Figure 3d). Furthermore, various acidic reagents, including sulfuric, nitric, and tosylic acids, were used for efficient photoreactions (Figure 3e). Weak acetic acid was also suitable for the material's degradation; however, the decrease in the rate of *G*′ was relatively slow (Figure S30b, Supporting Information), indicating that the synergistic reactivity required both light and diverse Brønsted acids.

The synergistic responsiveness to light and acid can be applied to the microfabrication of **G1** by exploiting the spatiotemporal resolution of light. In the presence of acid, light irradiation of **G1** through a photomask with a dotted pattern resulted in the micropatterned degradation of the irradiated area (Figure 3f). Furthermore, **G1′** (x/y = 100/0.03) was irradiated with 365‐nm UV light in the presence of acid through a photomask with 50‐µm wide line patterns (Figure S22a, Supporting Information). The gel was partially degraded in the exposed area, demonstrating 50‐µm scale resolution in microlithography using pyrenylsilicon‐crosslinked materials (Figure S22b, Supporting Information).

In addition, the material, after photoprocessing, regained its photostability when the acidic reagent was removed via solvent replacement. Photostability was evaluated by tracing the weight of the microfabricated gels with a dotted pattern under white fluorescent‐lamp irradiation (20 W). After the acidic reagent was removed, the gel became photostable under light for more than 140 h (Figure 3g). In contrast, in the presence of acid, the material significantly deformed with increasing weight of the gel within 24 h, owing to the photocleavage of the crosslinkers in the network. The pyrenylsilane‐crosslinked material drastically altered the photostable and photoprocessable states upon removing or adding acidic reagents, affording high stability in a light‐exposed environment over the long term, as well as microscale photoprocessability in the presence of acids.

### 2.3 Applications of Synergistic Reactivity to Functional Gels and Elastomers

**Figure 4:**
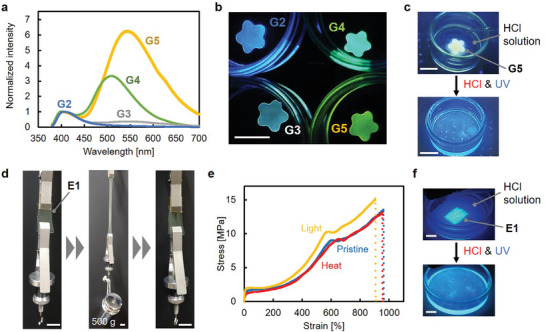
a) Normalized emission spectra of gels (**G2**–**G5**) under 365‐nm excitation. Solvent ratios (toluene/dimethylaniline/DMSO) of **G2**, **G3**, **G4**, and **G5** are 7/0.25/3, 7/1/3, 7/1/0, and 7/3/1, respectively. b) Photographs of **G2**, **G3**, **G4**, and **G5** under 365‐nm excitation. c) Photographs of **G5** before and after synergistic degradation with light and acid. d) Photographs of elastic material (**E1**) crosslinked by dipyrenysilicon crosslinker before and after elongation with a weight (500 g). e) Stress–strain curves of pristine (blue), heated (red), and irradiated (orange) **E1** (Heat: 150 °C for 24 h, Light: white fluorescent lamp (20 W) for 24 h). f) Photographs of **E1** before and after synergistic degradation with light and acid. Scale bar: 10 mm.

Synergistic light–acid reactive materials exhibit transient photocontrol and long‐term photostability, which can be applied to optical materials by removing acids. Here, the pyrenylsilane‐based crosslinking points in **G1** could be utilized as fluorescent chromophores under excitation at 365 nm without acid.^[^ ^34^, ^35^, ^36^ ^]^ The micropatterned **G1**, fabricated via synergistic degradation with 365‐nm light and HCl, as mentioned above, exhibited blue luminescence under 365‐nm excitation without photodegradation after removing the acid (Figure S32a, Supporting Information). In addition, the pyrene–dimethylaniline (DMA) exciplex was exploited for multi‐color luminescence.^[^ ^37^ ^]^ The emission color was controlled from blue to yellow by tuning the ratio between monomer and exciplex emissions, through changing the retaining solvent ratio of toluene/DMA/dimethyl sulfoxide (DMSO) (v/v) as 7/0.25/3 (**G2**), 7/1/3 (**G3**), 7/1/0 (**G4**), and 7/3/1 (**G5**) in polymer networks (**Figure** **4**a,b), in which toluene provided an efficient exciplex emission as a low‐polarity solvent, whereas DMSO induced a red‐shift of the emission wavelength as a high‐polarity solvent.^[^ ^38^, ^39^, ^40^ ^]^ The control of emission color was also achieved in the micropatterned gels via a synergistic light–acid reaction, followed by removing the acid (Figure S32c, Supporting Information). Furthermore, the multicolor luminescent gels were quickly photodegraded by immersion in HCl solution and exposure to 365‐nm light (Figure 4c). Hence, synergistically photodegradable materials can be exploited as photoexcitable materials because of their high photostability without acid. This is the first technology that renders fluorescent color‐tunable materials that are photodegradable and photoprocessable using irradiation of the same wavelength.

The synergistic degradability can be applied to structural materials involving robust elastomers without solvent retention. A pyrenylsilane‐crosslinked elastic sheet (**E1**), which was fabricated by removing the solvent of **G1**, could sustain a weight of 500 g with stretching and exhibited restoration to its original size after removing the weight (Figure 4d). Tensile tests of **E1** indicated the toughness of the materials with a Young's modulus of >25 MPa, tensile strain of >800%, and fracture energy of 50 MJ·m^−3^ (Figure 4e, Figure S35, Supporting Information). Moreover, durability tests with exposure to white fluorescent light or heating at 150 °C for 24 h demonstrated the material robustness of **E1**, because the mechanical properties were completely consistent with those of the pristine sample (Figure 4e, Figures S34 and S35, Supporting Information). The high robustness of **E1** under light and heat was in contrast to typical photoreactive materials, which can deteriorate through radical reactions induced by light or heat.^[^ ^41^, ^42^ ^]^ The high stability of synergistically reactive pyrenylsilane (**1a**) was also revealed by thermogravimetric analysis (TGA), demonstrating a decomposition temperature at >300 °C (Figure S39, Supporting Information). Despite the high robustness of **E1**, it was easily photodegraded in the presence of acids. By immersing the elastic sheet in an HCl solution and exposing it to light, **E1** decomposed through gradual swelling with the HCl solution (Figure 4f). Thus, synergistic light–acid reactivity with acids can be applied to structural materials with long‐term stability and toughness.

The synergistic reactivity enabled a new class of materials to be fabricated via photopolymerization and photodegradation with acid.^[^ ^23^ ^]^ Photopolymerization was conducted with methyl acrylate (1 equiv.) and crosslinker (**8**, 0.0003 equiv.) in the presence of a photoinitiator under light irradiation (365 nm, 0.3 mW·cm^−2^) to yield a transparent and elastic gel (See Supporting Information). The macroscopic degradability of the gel was confirmed by light irradiation (365 nm, 0.08 W·cm^−2^) in the presence of HCl, whereas the gel was photostable without the acid (Figure S40, Supporting Information), demonstrating the compatibility between photofabrication and photodegradation with the same wavelength of light irradiation, which has been conventionally regarded as an incompatible phototechnology owing to the interference of photoreactivity.

**Figure 5:**
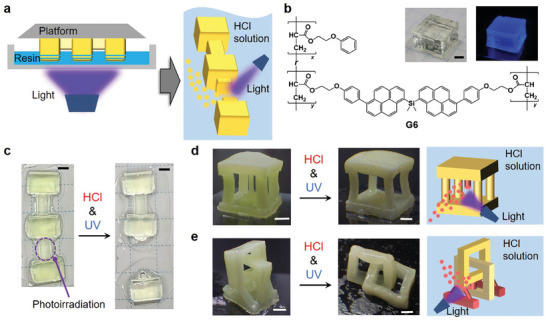
a) Schematic of light‐based 3D printing via layer‐by‐layer photopolymerization and its synergistic photoprocessing under acidic conditions. b) Chemical structure of polymer networks crosslinked by dipyrenylsilicon groups (**G6**, x/y = 100/0.03). Inset: Photographs of 3D‐printed cubic gel under ambient light (left) and 365‐nm UV irradiation (right). c) Photographs of 3D‐printed gel composed of three connected blocks before and after local degradation with 365‐nm UV light and HCl. d,e) Photographs and schematics of shrunken objects of d) cage and e) interlocked chain through 3D printing and subtractive manufacturing via synergistic light–acid degradation of the supporting objects (marked in pink in the schematic). Scale bar: 2 mm.

This compatibility can be applied to stereolithography, that is, light‐based 3D printing of photodegradable polymer materials. 3D printing has gained increasing attention in scientific and industrial fields owing to its capacity for precise and complicated manufacturing.^[^ ^43^, ^44^ ^]^ Light‐based 3D printing technology is based on layer‐by‐layer photopolymerization of polymer resin from the platform, yielding 3D objects of polymer networks (**Figure** **5**a).^[^ ^45^, ^46^ ^]^ The facile degradation of printed materials allows further processing of the materials via re/upcycling and subtractive manufacturing.^[^ ^47^, ^48^ ^]^ Here, 3D printing of the polymer network (**G6**) was conducted via photopolymerization of a resin composed of 2‐phenoxyethyl acrylate (1 equiv.), dipyrenylsilicon crosslinker (**8**, 0.0003 equiv.), and photoinitiator, using a consumer‐use 3D printer with handmade modifications (Figure 5b, Figure S41, Supporting Information), to yield a cubic structure swollen in DMF as a gel (Figure 5b, inset). Because pyrene moieties were successfully incorporated into the polymer network during 3D printing, the cubic objects exhibited blue luminescence under 365‐nm excitation as a photostable material. In contrast, after swelling in the HCl solution, the cubic structure was completely degraded under light irradiation, demonstrating the synergistic degradability of the 3D‐photoprinted gels (Figure S43, Supporting Information).

Moreover, the high spatial resolution of light enables local processing of 3D‐printed objects via a synergistic light–acid reaction. Another 3D‐printed gel, composed of three connected blocks, was locally photodegraded on one connector using HCl (Figure 5d). The irradiated target was completely degraded, whereas the non‐irradiated objects kept their shape perfectly. Furthermore, local operations can be applied to subtractive manufacturing of 3D‐printed objects as a practical material process. Typically, supporting objects are essential for preparing complicated 3D structures to assist their layer‐by‐layer fabrication.^[^ ^49^ ^]^ A cage object composed of two plates and four pillars was fabricated using a 3D printer assisted by another pillar as a supporting object (Figure 5d, Figure S42, Supporting Information). After fabricating the assisted cage structure, the supporting pillar was locally degraded under light irradiation with HCl to yield the target cage structure through local subtractive manufacturing. Similarly, an interlocked chain was fabricated via 3D printing, followed by local subtractive manufacturing (Figure 5e, Figure S44, Supporting Information). Accordingly, the synergistic light–acid reactivity of pyrenylsilicon crosslinkers allows for a new class of photoprocessing technologies for polymer network materials fabricated by light‐based 3D printing, directed toward material photodegradation and photomanufacturing.

## 3 Conclusion

In summary, synergistic light–acid reactivity was newly developed with pyrenylsilanes by exploiting the photoinduced dearomatization and following the protonation‐desilylation of pyrenylsilane. The C–Si bonds are cleaved under light in the presence of acidic reagents as a new class of synergistic light–acid reactivity. Conversely, the C–Si bonds remained intact under a single stimulus of light or acid. In particular, the photostability of pyrenylsilane was ≈100 folds greater than that of the previously reported platinum compound. The unique reactivity of pyrenylsilicon moiety could be applied to polymer network materials as crosslinkers, which were stable under light‐irradiated environments for the long term in the absence of acid. In addition to the degradation and micropatterning of their shapes and physical properties, they allowed diverse applications as optical materials, long‐term structural materials, and 3D‐photofabricated materials derived from the ubiquitous and robust pyrenylsilicon moiety. Their light‐irradiation applications are compatible with photodegradation and photoprocessing, which are conventionally regarded as conflicting photofunctions. These results demonstrate that synergistic reactivity could pave the way for overcoming the material trade‐off between photostability and photocontrollability, which would provide new material applications that are controllable and degradable with light irradiation.

## 4 Experimental Section

### Synthesis

The synthetic procedure and compound characterization data can be found in Supporting Information.

### Preparation of **G1**

A pregel solution containing monomers (methyl acrylate, 1 equiv.), crosslinkers (**8**, 0.0009 equiv.), and radical initiator (2,2′‐azobis(2,4‐dimethylvaleronitrile), 0.001 equiv.) in DMF was degassed three times via freeze‐thaw technique and was filled into the gap between two PTFE coated glass slides with a 0.3 mm or 0.5 mm PTFE thick spacer. The glass slides and spacer were held with binder clips. The reaction solution was then placed in an oven (60 °C, 18 h) for polymerization. After polymerization, the obtained network material was washed with solvents and then dried in a vacuum. The dried samples were reswollen with DMF to yield gel samples.

### General Procedure of Synergistic Light–Acid Processing

Disc‐shaped samples were cut from gels swollen in DMF. The samples were shrunk in MeOH and vacuumed to remove the original solvents in gels. Subsequently, the dried samples were immersed in the solvent according to reaction conditions for 3 h, to introduce the reaction solvent in gels. The gels were exposed to photo irradiation for synergistic light–acid processing. After the reaction, the gels were washed with EtOAc and MeOH and dried in a vacuum overnight. The dried samples were immersed in DMF and reswollen as the original solvent of gels.

### Partial Degradation of **G1**

For evaluating the macroscopic change of **G1** after UV irradiation in the absence and presence of acid, the **G1** samples were swollen in EtOAc or EtOAc with 0.5 m HCl, respectively. Each samples were irradiated with UV light (365 nm, 50 mW·cm^−2^) for 10 min in the center of the samples. After the stimulation, the solvent of gels was replaced to the original solvent (DMF) for the analyses.

### Rheological Measurements of **G1**

The rheological changes of disc‐shaped **G1** samples (14 mm in diameter, 0.6–0.8 mm in thickness) induced by photoprocessing was investigated in the presence and absence of HCl. As a standard condition, after holding the gel under a normal force of 1.5 N, the storage modulus (*G′*) of **G1** (*ν* = 0.09 mol%) in DMF/EtOAc (19/1 v/v) were measured under a constant gap while exposed to UV light (λ = 365 nm, 2 mW·cm^−2^) in the presence or absence of HCl (0.2 m). UV irradiation was started 60 s after the start of the rheology measurement except for the measurement of switching the UV light.

### Preparation of **E1**

The dumbbell‐shaped sample with a thickness of 0.5 mm was cut from **G1**. The cut gels were shrunk in MeOH and vacuumed at 50 °C for 24 h to obtain specimens of **E1** (a thickness was ≈0.2 mm).

### 3D Printing of **G6**

Crosslinker **8** (1.8 mg, 2.1 µmol) and phenylbis(2,4,6‐trimethylbenzoyl)phosphine oxide (29 mg, 69 µmol) were added to 2‐phenoxyethyl acrylate (1.2 mL, 6.9 mmol) and dissolved by sonication. The resulting solution was used as resin for 3D printing of gel materials. Printed gel materials were gently detached from the platform and washed by DMF and EtOAc with 0.5 m HCl.

### Local Photodegradation of **G6**

**G6** was swollen in large amounts of EtOAc with 0.5 m HCl and irradiated with a spot‐type UV light (CCS, 8332A‐AC8361‐AC8303 covered by aluminum foil with a 2 mm diameter hole, λ = 365 nm, 0.5 W·cm^−2^) for 2–10 min until photo‐degradation. After the degradation, the gel was washed with EtOAc, DMF, and MeOH and dried in a vacuum overnight. The dried sample was swollen in mixed solvent of DMF and MeOH.

## Conflict of Interest

The authors declare no conflict of interest.

## Supporting information



Supporting Information

## Data Availability

The data that support the findings of this study are available from the corresponding author upon reasonable request.
