# Supplementary material for: Synergistic Degradation of Durable Polymer Networks by Light and Acid Enabled by Pyrenylsilicon Crosslinks
Source: Adv Mater. 2024 Dec 4;37(3):2412544. doi: 10.1002/adma.202412544 (PMC11756034; doi:10.1002/adma.202412544)
Supplement: Supplementary file 1 — Supporting Information [file ADMA-37-2412544-s001.pdf]

ADVANCED MATERIALS

Supporting Information

for *Adv. Mater.*, DOI 10.1002/adma.202412544

Synergistic Degradation of Durable Polymer Networks by Light and Acid Enabled by Pyrenylsilicon Crosslinks

Yutaro Kawano, Hiroshi Masai, Takuya Tsubokawa, Daisuke Yokogawa, Tomohiro Iwai and Jun Terao**

Supporting Information

Synergistic Degradation of Durable Polymer Networks by Light and Acid Enabled by Pyrenylsilicon Crosslinks

Yutaro Kawano, Hiroshi Masai, Takuya Tsubokawa, Daisuke Yokogawa, Tomohiro Iwai, and Jun Terao**

Table of Contents

S1. General remarks S3

S1.1 Materials S3

S1.2 Instruments S3

S2. Synthetic procedures S5

S2.1 Synthesis of dipyrenylsilicon compounds S5

S2.2 Synthesis of gel materials via thermal-initiated radical polymerization S12

S3. Comparison of synergistic photoreactivity among 1a–1c S15

S3.1. General procedures S15

S4. Comparison of synergistic photoreactivity between 2 and S4 ..... S17

S4.1 General procedures S17

S4.2 ^{1}H NMR analyses of S4 ..... S18

S4.3 SEC and ^{1}H NMR analyses of 2 ..... S18

S4.4. Investigation of the effects of different HCl concentrations, intensities of UV light, and wavelengths of light exposure S23

S5. Synergistic photoreactivity of gel materials with acid S25

S5.1 General procedures of macroscopic processing S25

S5.2 Partial degradation of G1 ..... S25

S5.3 Micropatterning of G1 ..... S26

S5.4. Microlithography of G1 ..... S26

S5.5 Swelling of G7 ..... S27

S5.6 Photostability of G1 on white fluorescent light S28

S5.7 Rheological measurements on photoprocessing of G1, G8, G9, and G10 ..... S30

S6. Luminescence of gel materials S33

S6.1 Luminescence of micropatterned gel samples S33

S6.2 Synergistic photodegradation of luminescent materials S33

S7. Synergistic photodegradation of robust elastomers S34

S7.1 Preparation of E1 .... S34

S7.2 Elongation test of E1 .... S34

S7.3 Photostability and thermal stability of E1 .... S34

S7.4 Thermogravimetry analysis S36

S7.5 Synergistic photodegradation of E1 .... S37

S8. Synergistic photodegradation of photopolymerized materials S38

S8.1 Synthesis of G11 via photo-initiated radical polymerization S38

S8.2 Synergistic photodegradation of G11 .... S38

S8.3 3D printing of G6 .... S39

S8.4 Photodegradation of G6 .... S40

S8.5 Local photodegradation of G6 .... S40

S9. Computational calculations S41

S10. Absorption spectra S51

S11. NMR spectra S52

S11.1 ^{1}H NMR, ^{13}C{^{1}H} NMR, and ^{29}Si{^{1}H} NMR spectra S52

S11.2 2D NMR spectra S60

S12. Reference S63

S1. General remarks

S1.1 Materials

All reagents were commercially obtained and used as received unless otherwise noted. 0.2 M HCl solution in *N*,*N*-dimethylformamide (DMF)/ethyl acetate (EtOAc) (19/1, v/v) was obtained by dilution of 4 M HCl solution in EtOAc, purchased from FUJIFILM Wako Pure Chemical, with DMF. Similarly, 0.2 M, 0.5 M, and 1 M HCl solution in EtOAc were obtained by dilution of 4 M HCl solution with EtOAc. Degassed tetrahydrofuran (THF), degassed toluene, degassed CH_2Cl_2, and degassed DMF were purchased from Kanto Chemical and further purified by passage through activated alumina under positive nitrogen pressure as described by Grubbs et al.[1] Methyl acrylate was purchased from Kanto Chemical and purified by distillation. Dried potassium carbonate was purchased from FUJIFILM Wako Pure Chemical and dried by heating under vacuum. **S4** was prepared according to the previously reported procedures.[2]

S1.2 Instruments

NMR Spectroscopy: ^{1}H NMR (500 MHz), ^{13}C{^{1}H} NMR (126 MHz), ^{29}Si{^{1}H} NMR (99 MHz), and ^{1}H-^{1}H COSY NMR, NOESY NMR spectra were measured with a Bruker AVANCE III HD 500. The ^{1}H NMR chemical shifts were reported relative to tetramethylsilane (TMS, 0.00 ppm) or residual protonated solvents in $CDCl_3$ (7.26 ppm) or DMSO-d_6 (2.50 ppm). The ^{13}C{^{1}H} NMR chemical shifts were reported relative to $CDCl_3$ (77.16 ppm) or DMSO-d_6 (39.52 ppm). The ^{29}Si{^{1}H} NMR chemical shifts were reported by using TMS (0.00 pm) as an external standard.

High-Resolution Mass Spectroscopy (HR-MS): Electrospray ionization (ESI) mass spectra were recorded on micrOTOF II-KE02 *or* Exactive Plus using internal mass calibration with NaTFA cluster ions. Electron Ionization (EI) mass spectrum was recorded on JMS-SX102A.

Preparative Recycling Gel Permeation Chromatography (GPC): Preparative recycling GPC was performed with a Shimadzu LC-20AP System equipped with Shodex K-4002L column, a Shimadzu SPD-20A and a Shimadzu RID-10A, *or* a JAI LC9130NEXT System equipped with JAIGEL-2H column, a JAI UV DETECTOR 370NEXT, and a JAI RI DETECTOR RI 700NEXT, *or* a JAI LaboACE LC-5060 Plus II equipped with JAIGEL-2H column, using $CHCl_3$ as the eluent at flow rate of 14 $mL\cdot min^{-1}$.

Analytical Size-exclusion Chromatography (SEC): Analytical SEC was performed with a GL-Science GL-7400 HPLC System equipped with Shodex KF-802, -803, -804 columns, a GL-7410 HPLC pump, a GL-7400 UV detector, and a GL-7454 RI detector using THF as the eluent at a flow rate of 0.6 $mL\cdot min^{-1}$ or a Shimadzu LC-20A system equipped with Shim-pack GPC-802, a DGU-20A, a LC-20AD, a SIL-20A, a CBM-20A, a RID-20A, a SPD-20A, and a CTO-40C, using THF as the eluent at flow rate 1 $mL\cdot min^{-1}$.

Luminescence Spectroscopy: Emission spectra were measured with a Shimadzu RF-6000 Spectro Fluorophotometer.

UV light irradiation: A spot UV lamp (CCS, HLV-24UV365-4WNRBT, 365 nm, 8 mm in diameter) was used unless otherwise noted.

White Fluorescent Light: A white fluorescent lamp (Panasonic, EFD25ED20E, 20 W) was used to examine the stability of the gel and elastomer.

Rheology Measurement: Photorheological studies were carried out on a strain-controlled rheometer (Anton Paar, MCR302e), equipped with a spot-type UV source (Hamamatsu, LIGHTNINGCURE LC-L1, 365 nm). Samples were placed on the top of a plate and fixed with a round plate (12 mm in diameter). As a standard setting, a frequency of 1 Hz and a shear strain of 1% were maintained during the measurement.

Tensile testing: Tensile tests were performed with a Shimadzu EZ-SX tester equipped with a 50 N load-cell. The test piece was stretched at a constant crosshead speed of 50 $mm \cdot min^{-1}$ until the test piece failure. At least five test pieces were tested at room temperature, and their mean and standard error were calculated.

Thermogravimetry analysis (TGA): TGA was conducted with a Thermo Plus 2 thermogravimetric analyzer (Rigaku) with α-Al_2O_3 as a reference under a dry N_2 flow (100 $mL \cdot min^{-1}$) in the temperature range of 25−500 °C with an increasing rate of 5 $°C \cdot min^{-1}$.

3D printing: 3D printing of gel materials was carried out on a Elegoo Mars 3 pro. Handmade vat and platform were used for 3D printing.

S2. Synthetic procedures

S2.1 Synthesis of dipyrenylsilicon compounds

Scheme S1. Synthetic route to the dipyrenylsilicon derivatives.

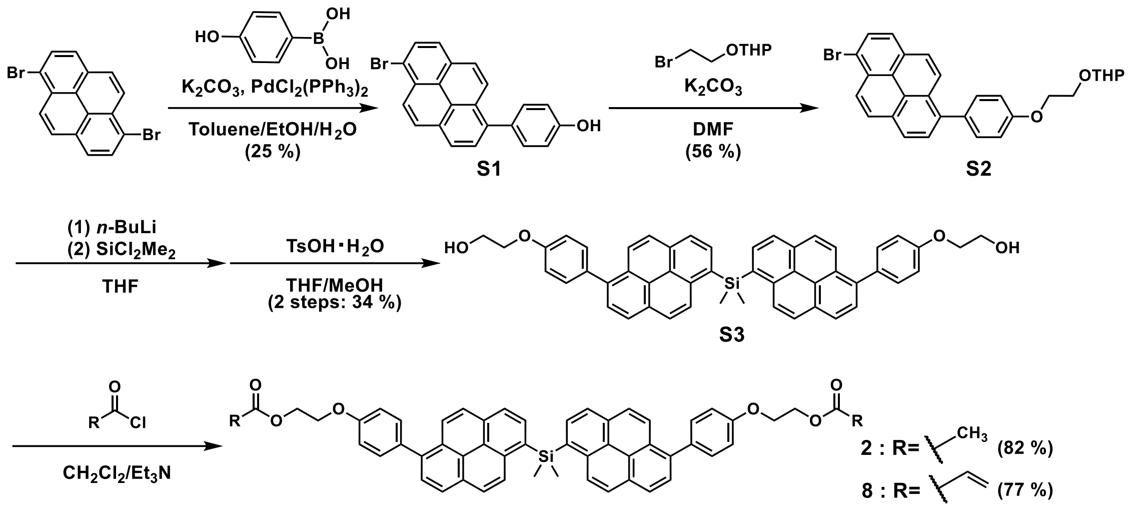

Scheme S2. Synthetic scheme of compound **2** via desilylation using fluoride ion.

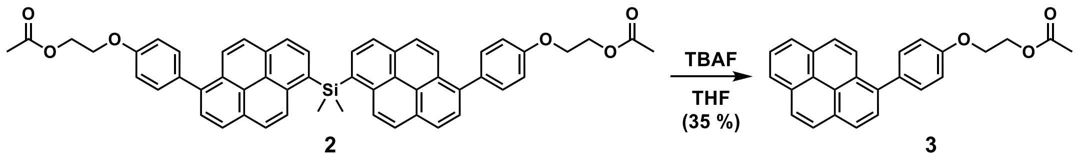

Synthesis of **S1**

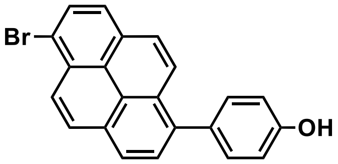

Under a nitrogen atmosphere, 1,6-dibromopyrene (20.0 g, 55.6 mmol), 4-hydroxyphenylboronic acid (6.13 g, 44.4 mmol), and potassium carbonate (15.3 g, 111 mmol) were added in degassed toluene (160 mL), ethanol (80 mL), and H_2O (40 mL), which were degassed by bubbling N_2 for 10 min. Dichloro(bistriphenylphosphine)palladium (390 mg, 0.556 mmol) was added to the solution. The reaction mixture was stirred at 70 °C overnight. The mixture was quenched with H_2O, filtered, and washed with chloroform. The filtrate was extracted with chloroform. The organic layer was separated and dried over $MgSO_4$, filtrated, and evaporated *in vacuo*. The residue was purified by column chromatography on silica gel (hexane/CH_2Cl_2 1:1 to 0:1) and washed with hexane to yield **S1** as a white solid (4.10 g, 25%).

EI HR-MS: (m/z) 372.0149 ($[M]^+$ $C_{22}H_{13}BrO$ calcd. 372.0150).
^1H NMR (500 MHz; DMSO-d_6): δ_H 9.73 (s, 1H), 8.38 (d, J = 7.9 Hz, 1H), 8.33 (m, 3H), 8.19–8.12 (m, 3H),

8.02 (d, J = 7.8 Hz, 1H), 7.44 (d, J = 8.4 Hz, 2H), 7.01 (d, J = 8.4 Hz, 2H).
$^{13}C\{^1H\}$ *NMR (126 MHz; DMSO-d*$_6$*)*: δ_C 157.12, 138.36, 130.56, 130.17, 130.06, 130.05, 129.38, 129.33, 129.30, 129.01, 128.41, 127.68, 127.03, 125.59, 125.55, 125.32, 124.75, 123.53, 118.93, 115.49.

Synthesis of **S2**

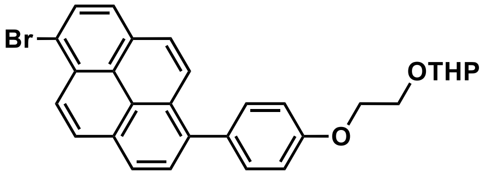

Under a nitrogen atmosphere, **S1** (4.00 g, 10.7 mmol) and dried potassium carbonate (4.44 g, 32.2 mmol) were added in degassed *N*,*N*-dimethylformamide (DMF) (70 mL) and 2-(2-bromoethoxy)tetrahydro-2*H*-pyran (2.24 mL, 16.1 mmol) was added to the solution. The reaction mixture was stirred at 60 °C overnight. The mixture was quenched with water, and extracted with CH_2Cl_2. The combined organic layer was washed with brine, dried over Na_2SO_4, filtered, and evaporated *in vacuo*. The residue was washed with methanol and purified by column chromatography on silica gel (CH_2Cl_2) to yield **S2** as a yellow solid (3.10 g, 58%).

ESI HR-MS:(m/z) 523.08574 ($[M+Na]^+$ $C_{29}H_{25}BrO_3Na$ calcd. 523.08793).
^1H *NMR (500 MHz; CDCl*$_3$*)*; δ_H 8.39 (d, J = 9.2 Hz, 1H), 8.18 (m, 3H), 8.12 (d, J = 9.2 Hz, 1H), 7.97 (d, J = 7.8 Hz, 1H), 7.91 (dd, J = 8.4, 6.0 Hz, 2H), 7.52 (d, J = 8.3 Hz, 2H), 7.13 (d, J = 8.3 Hz, 2H), 4.79 (s, 1H), 4.28 (d, J = 4.4 Hz, 2H), 4.15 (dt, J = 10.6, 5.0 Hz, 1H), 4.00–3.90 (m, 2H), 3.61–3.59 (m, 1H), 1.92–1.57 (m, 6H).
$^{13}C\{^1H^\}$ *NMR (126 MHz; CDCl*$_3$*)*: δ_C 158.54, 138.31, 133.48, 131.69, 130.50, 130.18, 130.17, 129.97, 129.05, 128.66, 128.37, 127.03, 126.16, 125.76 (peaks overlapped), 125.25 (peaks overlapped), 124.42, 119.90, 114.76, 99.15, 67.68, 66.00, 62.36, 30.68, 25.57, 19.53.

Synthesis of **S3**

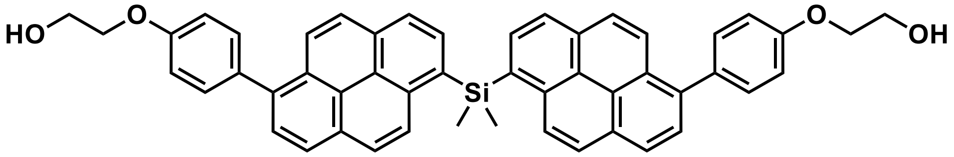

Under a nitrogen atmosphere, **S2** (3.00 g, 5.99 mmol) was dissolved in degassed THF (150 mL). *n*-Buthyllithium (1.6 M in *n*-hexane) (4.5 mL, 7.2 mmol) was slowly added to the solution at below –70 °C and the reaction mixture was stirred at below –70 °C for 30 min. Dimethyldichlorosilane (325 μL, 2.70 mmol) was slowly added to the mixture at below –70 °C. The mixture was allowed to warm to room temperature and stirred overnight. The mixture was quenched with H_2O at room temperature, and extracted with CH_2Cl_2. The combined organic layer was washed with brine, dried over $MgSO_4$, filtered, and evaporated *in vacuo*. The residue was purified by GPC with $CHCl_3$ as the eluent to yield as a yellow solid. The solid was dissolved in degassed THF (40 mL) and methanol (40 mL). *p*-Toluenesulfonic acid monohydrate (177 mg, 0.932 mmol) was added to the solution and the mixture was stirred at room temperature overnight. The mixture was

quenched with H_2O at room temperature, and extracted with CH_2Cl_2. The combined organic layer was washed with brine, dried over $MgSO_4$, filtered, and evaporated *in vacuo*. The residue was purified by washing with methanol to yield **S3** as a yellow solid (663 mg, 34%, 2 steps yield).

ESI HR-MS:(m/z) 755.26354 ($[M+Na]^+$ $C_{50}H_{40}O_4SiNa$ calcd. 755.25881).
^1H NMR (500 MHz; DMSO-d_6): δ_H 8.51 (d, *J* = 7.7 Hz, 2H), 8.37 (d, *J* = 7.7 Hz, 2H), 8.20–8.12 (m, 8H), 7.94–7.92 (m, 4H), 7.51 (d, *J* = 8.6 Hz, 4H), 7.16 (d, *J* = 8.7 Hz, 4H), 4.94 (t, *J* = 5.5 Hz, 2H), 4.10 (t, *J* = 4.9 Hz, 4H), 3.79 (q, *J* = 5.0 Hz, 4H), 1.08 (s, 6H).
$^{13}C\{^1H\}$ NMR (126 MHz; DMSO-d_6): δ_C 158.20, 137.21, 135.37, 134.17, 132.82, 132.32, 131.74, 131.40, 129.08, 127.75, 127.71, 127.54, 127.25, 126.82, 125.48, 125.13, 124.39, 124.28, 124.19, 114.58, 69.64, 59.61, 0.18.

Synthesis of **2**

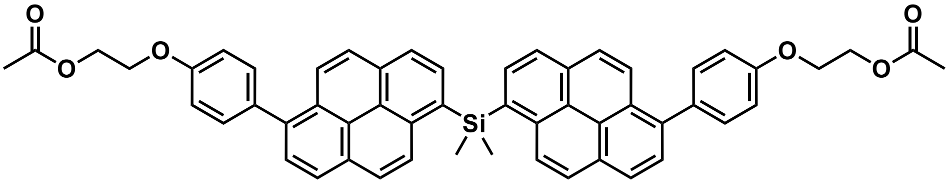

Under a nitrogen atmosphere, **S3** (300 mg, 0.410 mmol) was dissolved in degassed CH_2Cl_2 (30 mL) and triethylamine (800 μL) and acetyl chloride (233 μL, 3.28 mmol) were added to the solution at 0 °C. The mixture was allowed to warm to room temperature and stirred for 2 h. After the reaction was quenched with saturated $NaHCO_3$ aq., extracted with CH_2Cl_2. The combined organic layer was washed with brine, dried over $MgSO_4$, filtered, and evaporated *in vacuo*. The residue was purified by GPC with $CHCl_3$ as the eluent to yield **2** as a yellow solid (275 mg, 82%).

ESI HR-MS:(m/z) 839.2804 ($[M+Na]^+$ $C_{54}H_{44}O_6SiNa$ calcd. 839.2799).
^1H NMR (500 MHz; $CDCl_3$): δ_H 8.44 (d, *J* = 7.6 Hz, 2H), 8.24 (d, *J* = 9.2 Hz, 2H), 8.20 (d, *J* = 8.7 Hz, 4H), 8.07 (d, *J* = 7.9 Hz, 2H), 8.04 (d, *J* = 9.4 Hz, 2H), 7.89 (d, *J* = 7.8 Hz, 2H), 7.82 (d, *J* = 9.2 Hz, 2H), 7.53 (d, *J* = 8.6 Hz, 4H), 7.10 (d, *J* = 8.6 Hz, 4H), 4.51 (t, *J* = 4.7 Hz, 4H), 4.29 (t, *J* = 4.7 Hz, 4H), 2.16 (s, 6H), 1.11 (s, 6H).
$^{13}C\{^1H\}$ NMR (126 MHz; $CDCl_3$): δ_C 171.16, 158.01, 137.55, 136.34, 134.68, 134.28, 133.02, 132.39, 131.85, 129.97, 128.81, 127.78, 127.76, 127.61, 127.36, 126.09, 125.26, 125.17, 125.01, 124.36, 114.65, 66.21, 63.01, 21.08, 0.75.

Synthesis of **8**

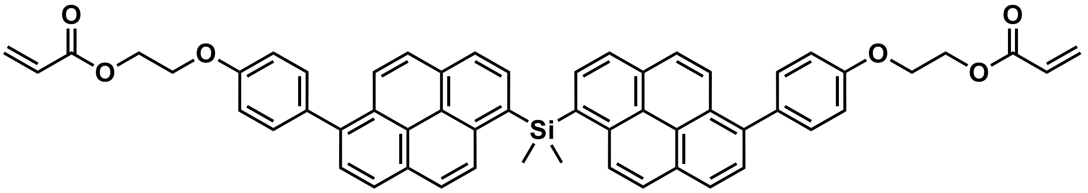

Under a nitrogen atmosphere, **S3** (660 mg, 0.902 mmol) was dissolved in degassed CH_2Cl_2 (75 mL) and triethylamine (1.6 mL) and acryloyl chloride (586 μL, 7.21 mmol) was added to the solution at 0 °C. The mixture was allowed to warm to room temperature and stirred for 2 h. The solution was diluted in CH_2Cl_2, then washed by water and 1 M HCl aq. The combined organic layer was washed with brine, dried over $MgSO_4$, filtered, and evaporated *in vacuo*. The residue was purified by GPC with $CHCl_3$ as the eluent to yield **8** as a yellow solid (580 mg, 77%).

ESI HR-MS:(m/z) 866.28918 ($[M+Na]^+$ $C_{56}H_{44}O_6SiNa$ calcd. 866.28987).
^1H NMR (500 MHz; $CDCl_3$): δ_H 8.44 (d, J = 7.6 Hz, 2H), 8.26–8.20 (m, 6H), 8.07 (d, J = 7.9 Hz, 2H), 8.04 (d, J = 9.3 Hz, 2H), 7.89 (d, J = 7.8 Hz, 2H), 7.82 (d, J = 9.2 Hz, 2H), 7.53 (d, J = 8.6 Hz, 4H), 7.11 (d, J = 8.6 Hz, 4H), 6.51 (dd, J = 17.3, 1.2 Hz, 2H), 6.23 (dd, J = 17.3, 10.4 Hz, 2H), 5.90 (dd, J = 10.4, 1.3 Hz, 2H), 4.60 (t, J = 4.7 Hz, 4H), 4.33 (t, J = 4.7 Hz, 4H), 1.11 (s, 6H).
$^{13}C\{^1H\}$ NMR (126 MHz; $CDCl_3$): δ_C 166.25, 158.03, 137.55, 136.33, 134.67, 134.27, 133.02, 132.38, 131.85, 131.51, 129.96, 128.80, 128.25, 127.77, 127.75, 127.61, 127.36, 126.09, 125.26, 125.17, 125.01, 124.35, 114.68, 66.20, 63.09, 0.75.
^{29}Si NMR (99 MHz; $CDCl_3$): δ_{Si} –7.03.

Synthesis of **3**

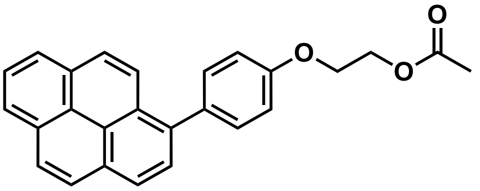

Under a nitrogen atmosphere, **2** (275 mg, 0.337 mmol) was dissolved in degassed THF (10 mL) and tetrabutylammonium fluoride (1 M in THF) (1.2 mL) was added to the solution. The reaction mixture was stirred at room temperature for 30 h. After the reaction was quenched with brine, extracted with EtOAc. The combined organic layer was washed with brine, dried over $MgSO_4$, filtered, and evaporated *in vacuo*. The residue was purified by GPC with $CHCl_3$ and washed with hexane as the eluent to yield **3** as a white solid (90 mg, 35%).

ESI HR-MS:(m/z) 403.12814 ($[M+Na]^+$ $C_{26}H_{20}O_3Na$ calcd. 403.13047).
^1H NMR (500 MHz; CDCl$_3$): δ_H 8.22–8.16 (m, 4H), 8.09 (s, 2H), 8.02 (m, 2H), 7.96 (d, *J* = 7.8 Hz, 1H), 7.57 (d, *J* = 8.4 Hz, 2H), 7.11 (d, *J* = 8.5 Hz, 2H), 4.51 (t, *J* = 4.7 Hz, 2H), 4.29 (t, *J* = 4.7 Hz, 2H), 2.16 (s, 3H).
$^{13}C\{^1H\}$ NMR (126 MHz; CDCl$_3$): δ_C 171.16, 158.04, 137.44, 134.26, 131.83, 131.65, 131.14, 130.56, 128.72, 127.78, 127.56, 127.51, 127.43, 126.12, 125.42, 125.17, 125.14, 125.08, 124.88, 124.77, 114.69, 66.23, 63.02, 21.07.

Synthesis of **1a**

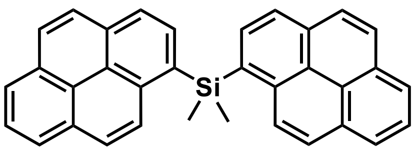

Under a nitrogen atmosphere, 1-bromopyrene (2.00 g, 7.14 mmol) was dissolved in degassed THF (20 mL). *n*-Buthyllithium (1.6 M in *n*-hexane) (4.91 mL) was slowly added to the solution at below –70 °C and the reaction mixture was stirred at below –70 °C for 1 h. Dimethyldichlorosilane (388 μL, 3.21 mmol) was dissolved in THF (5 mL) and the solution was slowly added to the mixture at below –70 °C. The mixture was allowed to warm to room temperature and stirred 2 h. The mixture was quenched with H_2O at room temperature, and extracted with CH_2Cl_2. The combined organic layer was washed with brine, dried over $MgSO_4$, and filtered. The filtrate was left at room temperature for 1 h. The produced solid was filtered. The filtrate was evaporated and the residue was washed with CH_2Cl_2. These solids were combined to yield **1a** as a white solid (1.02 g, 69%).

^{1}H NMR (500 MHz; $CDCl_3$): δ_H 8.41 (d, *J* = 7.6 Hz, 2H), 8.22 (d, *J* = 8.8 Hz, 4H), 8.16 (d, *J* = 7.5 Hz, 2H), 8.10 (s, 4H), 8.06 (d, *J* = 7.6 Hz, 2H), 7.94 (t, *J* = 7.6 Hz, 2H), 7.82 (d, *J* = 9.2 Hz, 2H), 1.09 (s, 6H).
The ^{1}H NMR spectrum was consistent with that previously reported.[3]

Synthesis of **1b**

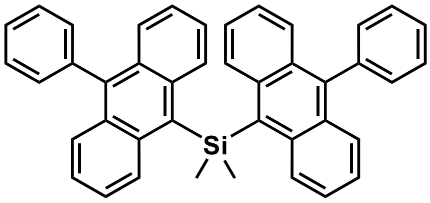

Under a nitrogen atmosphere, 9-bromo-10-phenylanthracene (495 mg, 1.49 mmol) was dissolved in degassed THF (20 mL). *n*-Buthyllithium (1.6 M in *n*-hexane) (1.0 mL, 1.6 mmol) was slowly added to the solution at below –70 °C and the reaction mixture was stirred at below –70 °C for 30 min. Dimethyldichlorosilane (87 μL, 0.72 mmol) was slowly added to the mixture at below –70 °C. The mixture was allowed to warm to room temperature and stirred overnight. The mixture was quenched with H_2O at room temperature, and extracted with CH_2Cl_2. The combined organic layer was dried over $MgSO_4$, and filtered. The filtrate was evaporated *in vacuo*. The residue was purified by GPC with $CHCl_3$ to yield **1b** as a yellow solid (351 mg, 86%).

ESI HR-MS:(m/z) 587.21753 ($[M+Na]^+$ $C_{34}H_{28}SiNa$ calcd. 587.21655).
^{1}H NMR (500 MHz; $CDCl_3$): δ_H 8.67 (d, J = 9.9 Hz, 4H), 7.67–7.64 (m, 4H), 7.60–7.56 (m, 4H), 7.55–7.51 (m, 2H), 7.44–7.42 (m, 4H), 7.25–7.22 (m, 8H), 1.34 (s, 6H).
^{13}C{^{1}H} NMR (126 MHz; $CDCl_3$): δ_C 139.98, 139.29, 137.55, 135.89, 131.13, 130.02, 128.33, 128.29, 128.14, 127.42, 124.82, 124.41, 8.56.

Synthesis of **1c**

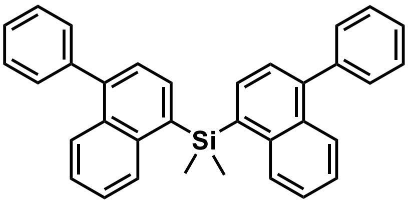

Under a nitrogen atmosphere, 1-bromo-4-phenylnaphthalene (1.20 g, 4.24 mmol) was dissolved in degassed THF (20 mL). *n*-Buthyllithium (1.6 M in *n*-hexane) (2.9 mL, 4.6 mmol) was slowly added to the solution at below –70 °C and the reaction mixture was stirred at below –70 °C for 30 min. Dimethyldichlorosilane (245 μL, 2.03 mmol) was slowly added to the mixture at below –70 °C. The mixture was allowed to warm to room temperature and stirred overnight. The mixture was quenched with H_2O at room temperature, and extracted with CH_2Cl_2. The combined organic layer was dried over $MgSO_4$, and filtered. The filtrate was evaporated and the residue was washed with hexane to yield **1c** as a white solid (730 mg, 77%).

ESI HR-MS:(m/z) 487.18721 ($[M+Na]^+$ $C_{34}H_{28}SiNa$ calcd. 487.18525).
^1H NMR (500 MHz; $CDCl_3$): δ_H 8.15 (d, J = 7.5 Hz, 2H), 7.96–7.94 (m, 2H), 7.93 (d, J = 7.0 Hz, 2H), 7.54–7.49 (m, 8H), 7.46–7.43 (m, 4H), 7.40–7.34 (m, 4H), 0.94 (s, 6H).
$^{13}C\{^1H\}$ NMR (126 MHz; $CDCl_3$): δ_C 142.12, 140.85, 137.38, 136.39, 133.96, 131.73, 130.08, 128.81, 128.26, 127.31, 126.97, 126.37, 125.61, 125.50, 0.18.

S2.2 Synthesis of gel materials via thermal-initiated radical polymerization

Gels were synthesized via thermal-initiated radical polymerization of the corresponding monomer and crosslinker. A pregel solution containing monomers, crosslinkers, and radical initiator in DMF, as shown in **Table S1** was degassed three times via freeze-thaw technique and was filled into the gap between two PTFE coated glass slides with a 0.3 mm or 0.5 mm PTFE thick spacer. The glass slides and spacer were held with binder clips (**Figure S1**). The reaction solution was then placed in an oven (60 °C, 18 h) for polymerization. After polymerization, the obtained network material was washed with solvents and then dried in a vacuum. The dried samples were reswollen with DMF to yield gel samples.

Synthesis of ***G1***

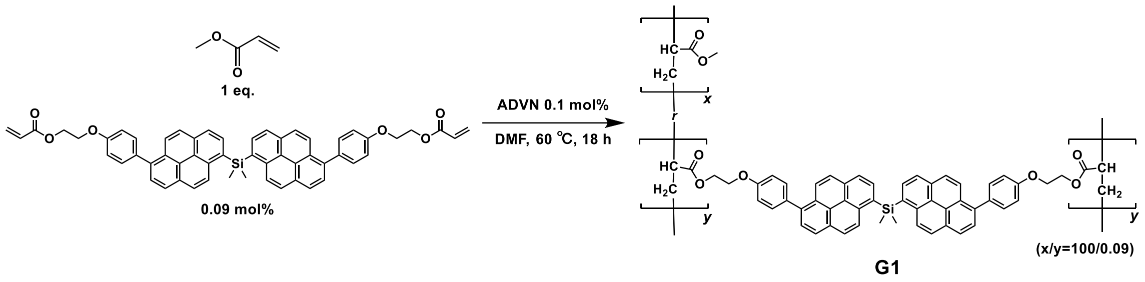

A pregel solution of **G1** was placed in an oven (60 °C, 18 h) for polymerization to obtain a transparent and elastic gel. The gel sample was washed with large amount of DMF and CH_2Cl_2, followed by shrinkage in methanol and drying in a vacuum. The dried samples were reswollen with DMF to yield **G1** samples.

Synthesis of ***G7***

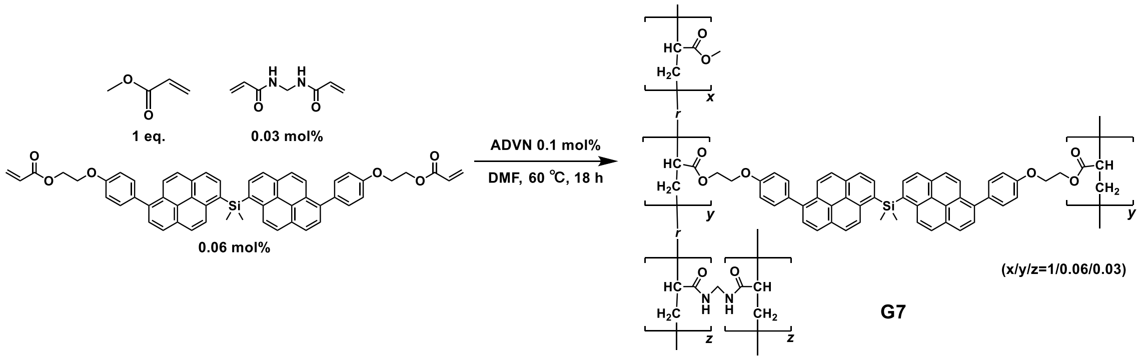

A pregel solution of **G7** was placed in an oven (60 °C, 18 h) for polymerization to obtain a transparent and elastic gel. The gel sample was washed with large amount of DMF and CH_2Cl_2, followed by shrinkage in methanol and drying in a vacuum. The dried samples were reswollen with DMF to yield **G7** samples.

Synthesis of ***G8***

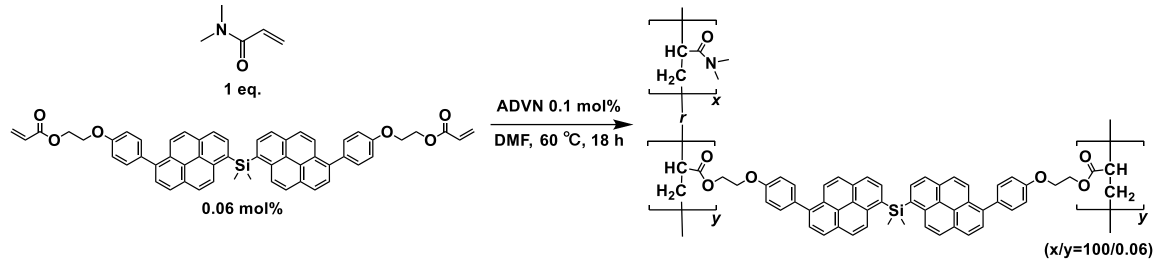

G8

A pregel solution of **G8** was placed in an oven (60 °C, 18 h) for polymerization to obtain a transparent and elastic gel. The gel sample was washed with large amount of DMF, followed by shrinkage in acetone and drying in a vacuum. The dried samples were reswollen with DMF to yield **G8** samples.

Synthesis of ***G9***

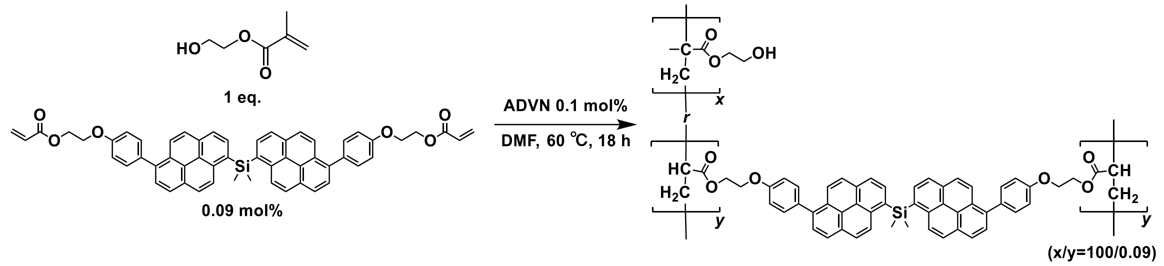

G9

A pregel solution of **G9** was placed in an oven (60 °C, 18 h) for polymerization to obtain a transparent and elastic gel. The gel sample was washed with large amount of DMF, followed by shrinkage in acetone and drying in a vacuum. The gel sample was washed with large amount of DMF, followed by shrinkage in acetone and drying in a vacuum. The dried samples were reswollen with DMF to yield **G9** samples.

Synthesis of ***G10***

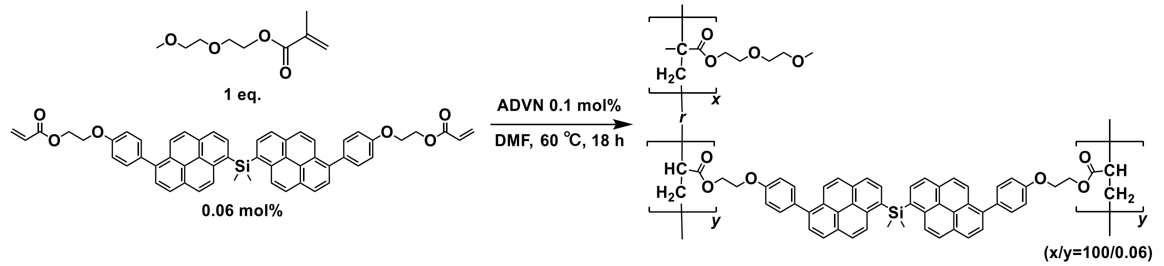

G10

A pregel solution of **G10** was placed in an oven (60 °C, 18 h) for polymerization to obtain a transparent and elastic gel. The gel sample was washed with large amount of DMF and CH_2Cl_2, followed by shrinkage in methanol and drying in a vacuum. The gel sample was washed with large amount of DMF, followed by shrinkage in acetone and drying in a vacuum. The dried samples were reswollen with DMF to yield **G10** samples.

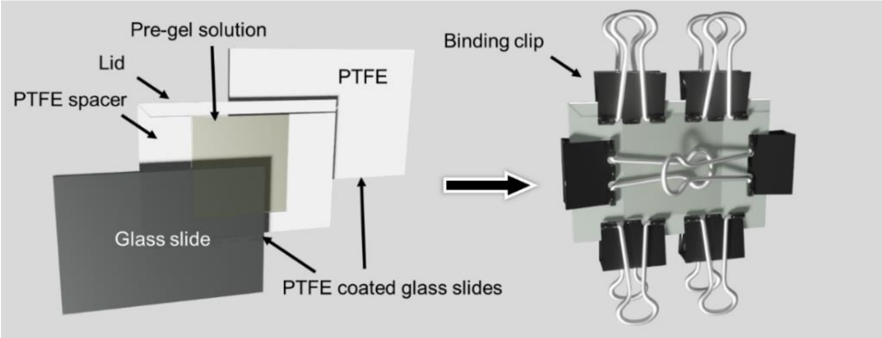

Figure S1. Schematic representation of procedure adopted for preparing the gels.

Table S1. Summary of composition of the reaction solution for the synthesis of gels.

Gels	Monomer			Crosslinker				ADVN		DMF
				**8**		MBA				
	Reagent	[μL]	[mmol]	[mg]	[μmol]	[mg]	[μmol]	[mg]	[μmol]	[μL]
**G1** (0.3 mm)	MA	372	4.1	3.1	3.7	—	—	1.0	4.1	173
**G1** (0.5 mm)	MA	600	6.7	5.1	6.0	—	—	1.7	6.7	281
**G1′** (0.3 mm)	MA	372	4.1	1.0	1.2	—	—	1.0	4.1	173
**G7** (0.3 mm)	MA	372	4.1	1.9	2.3	0.17	1.1	1.0	4.1	173
**G8** (0.5 mm)	DMAA	700	6.8	3.5	4.1	—	—	1.7	6.8	286
**G9** (0.5 mm)	HEMA	600	4.9	3.7	4.4	—	—	1.2	4.9	281
**G10** (0.5 mm)	DEGMA	700	3.8	1.9	2.2	—	—	0.94	3.8	150

MA: Methyl acrylate, MBA: *N*,*N'*-Methylenebisacrylamide, ADVN: 2,2'-Azobis(2,4-dimethylvaleronitrile), DMAA: *N*,*N*-Dimethylacrylamide, HEMA: 2-Hydroxyethy methacrylate, DEGMA: Diethylene glycol monomethyl ether methacrylate. The thickness of PTFE spacers during the polymerization was shown in parentheses.

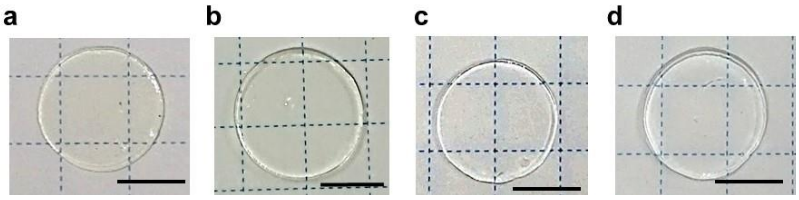

Figure S2. Photographs of (**a**) **G1**, (**b**) **G8**, (**c**) **G9**, and (**d**) **G10** after cutting into a disk shape. Scale bar: 5 mm.

S3. Comparison of synergistic photoreactivity among 1a–1c

S3.1. General procedures

The stimuli-responsiveness of **1a**, **1b** and **1c** was compared through ^{1}H NMR analyses (**Figure S4–S6**). A solution of each reagent (1.04 μmol) in the reaction solvent (2.2 mL) was exposed to stimulus for 30 min as shown below (UV, HCl, or HCl & UV). After the reaction, 800 μL of Et_3N was added to the solution at 0 °C, and the solution was evaporated. THF was added to the residue and the solution was filtered and evaporated. THF was added to the residue and the solution was filtered and evaporated. The residue was dissolved in $CDCl_3$ (700 μL) for ^{1}H NMR analysis (**Figure S3**).

Stimulation with UV light (noted as UV)

To subject **1a**, **1b**, and **1c** to UV light, each reagent was dissolved in THF/EtOAc (5/3, v/v) and exposed to UV light (λ = 365 nm, 0.3 W cm^{-2}). The solution was stirred at room temperature for 30 min.

Stimulation with HCl (noted as HCl)

To subject **1a**, **1b**, and **1c** to HCl, each reagent was dissolved in 1.5 M HCl solution in THF/EtOAc (5/3, v/v). The solution was stirred at room temperature for 30 min.

Stimulation with HCl and UV light (noted as HCl & UV)

To subject **1a**, **1b**, and **1c** to HCl and UV light, each reagent was dissolved in 1.5 M HCl solution in THF/EtOAc (5/3, v/v) and exposed to UV light (λ = 365 nm, 0.3 W·cm^{-2}). The solution was stirred at room temperature for 30 min.

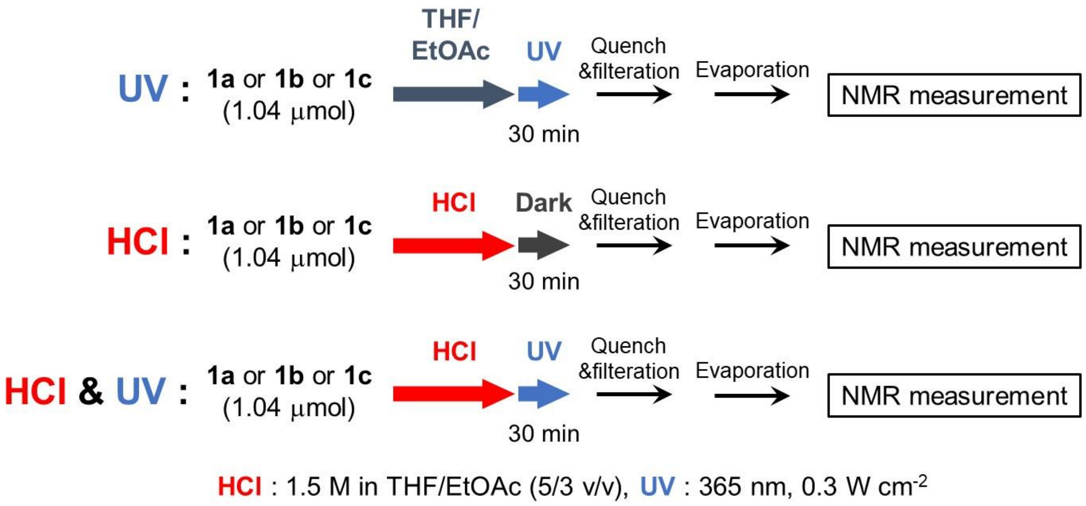

Figure S3. A detailed schematic timeline of the methods of stimulating **1a**, **1b**, and **1c** under different conditions.

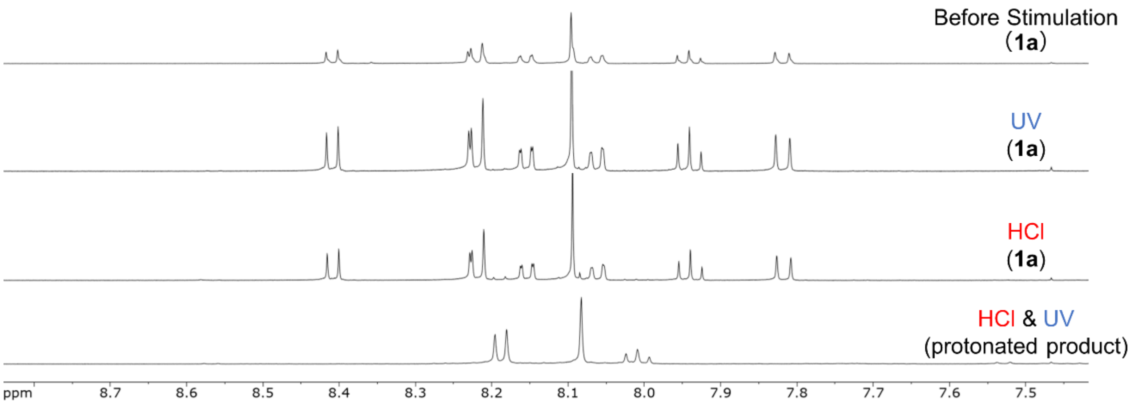

Figure S4. ^{1}H NMR spectra (500 MHz, $CDCl_3$, r.t.) recorded before and after the reaction of **1a** under three different stimuli for 30 min (UV: 365 nm, 0.3 W·cm^{-2}; HCl: 1.5 M HCl).

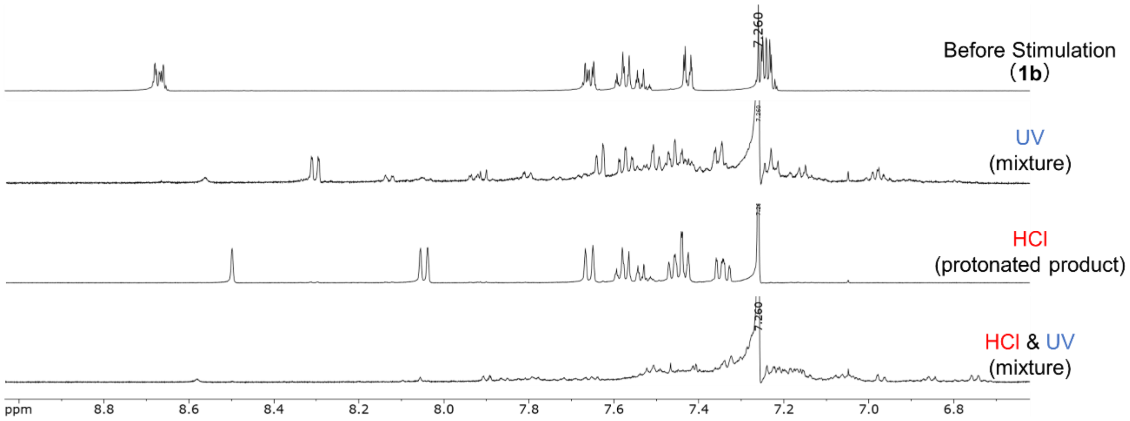

Figure S5. ^{1}H NMR spectra (500 MHz, $CDCl_3$, r.t.) recorded before and after the reaction of **1b** under three different stimuli for 30 min (UV: 365 nm, 0.3 W·cm^{-2}; HCl: 1.5 M HCl).

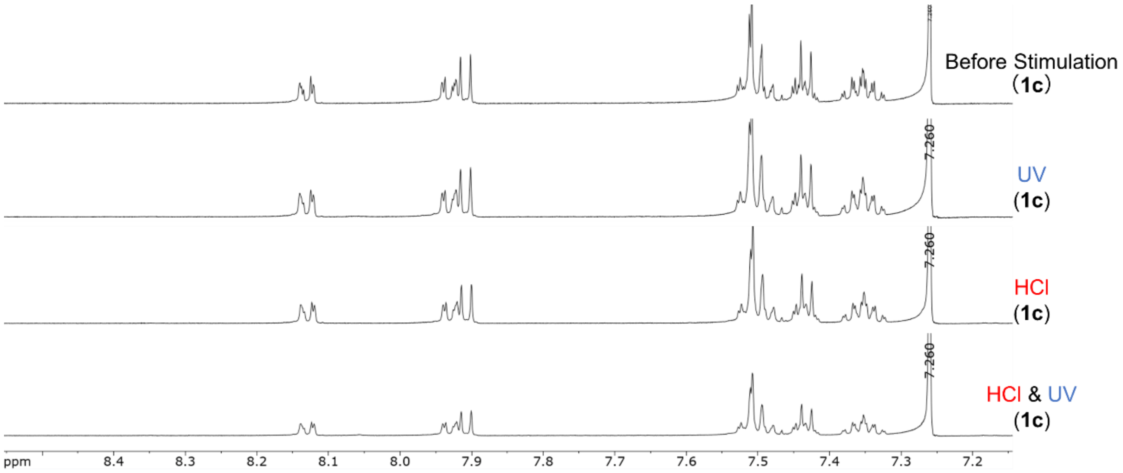

Figure S6. ^{1}H NMR spectra (500 MHz, $CDCl_3$, r.t.) recorded before and after the reaction of **1c** under three different stimuli for 30 min (UV: 365 nm, 0.3 W·cm^{-2}; HCl: 1.5 M HCl).

S4. Comparison of synergistic photoreactivity between 2 and S4

S4.1 General procedures

The stimuli-responsiveness of **2** and **S4** under UV irradiation alone, HCl alone, or UV irradiation in the presence of HCl was investigated by ^{1}H NMR and SEC analyses. A solution of each reagent (1.04 μmol) in the reaction solvent (2.2 mL) was exposed to stimulus for 30 min as shown below (UV, HCl, or HCl & UV). After the reaction, 500 μL of Et_3N was added to the solution at 0 °C, and the solution was evaporated. THF was added to the residue and the solution was filtered and evaporated. The residue was dissolved in THF for SEC analysis, while the residue was dissolved in $CDCl_3$ (700 μL) with 1,3,5-trimethoxybenzene (0.18 mg) as an internal standard for ^{1}H NMR analysis (**Figure S7**).

Stimulation with UV light

To subject **2** and **S4** to UV light, each reagent was dissolved in EtOAc and exposed to UV light (λ = 365 nm, 0.3 W·cm^{-2} or 0.8 W·cm^{-2}). The solution was stirred at room temperature for 30 min.
To subject **2** to stronger UV light, **2** was dissolved in EtOAc and exposed to UV light (λ = 365 nm, 2.4 W·cm^{-2}). The solution was stirred at room temperature for 60 min.

Stimulation with HCl

To subject **2** and **S4** to HCl, each reagent was dissolved in 0.5 M HCl solution in EtOAc. The solution was stirred at room temperature for 30 min.
To subject **2** to harsher HCl, **2** was dissolved in 2 M HCl solution in EtOAc. The solution was stirred at room temperature for 30 min.

Stimulation with HCl and UV light

To subject **2** and **S4** to HCl and UV light, each reagent was dissolved in 0.5 M HCl solution in EtOAc and exposed to UV light (λ = 365 nm, 0.3 W·cm^{-2}). The solution was stirred at room temperature for 30 min.

Stimulation with HCl and UV light in the anaerobic atmosphere

To subject **2** to HCl and UV light in the anaerobic atmosphere, **2** was dissolved in 0.5 M HCl solution in EtOAc. The solution was degassed by N_2 bubbling for 10 min. The solution was stirred at room temperature for 30 min under exposure to UV light (λ = 365 nm, 0.3 W·cm^{-2}).

Stimulation with HCl and UV light in the presence of TEMPO radical

To subject **2** to HCl and UV light in the presence of 2,2,6,6-tetramethylpiperidine 1-oxyl (TEMPO) radical, **2** and TEMPO radical (1.9 mg, 10 equiv.) was dissolved in 0.5 M HCl solution in EtOAc. The solution was stirred at room temperature for 30 min under exposure to UV light (λ = 365 nm, 0.3 W·cm^{-2}).

Stimulation with UV light in the presence of TEMPO radical

To subject **2** to UV light in the presence of TEMPO radical, **2** and TEMPO radical (3.8 mg, 20 equiv.) was dissolved in EtOAc. The solution was stirred at room temperature for 30 min under exposure to UV light (λ = 365 nm, 0.3 W·cm^{-2}).

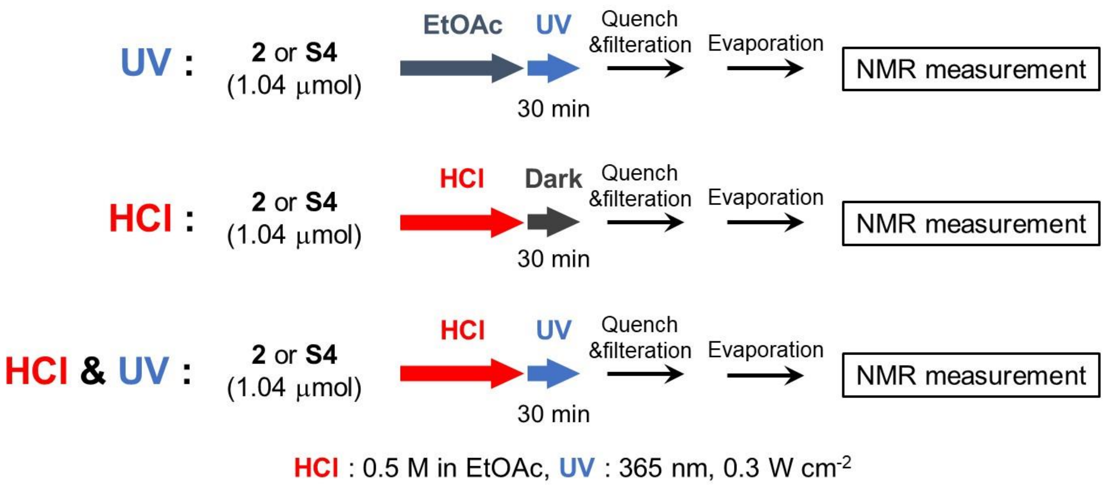

Figure S7. A detailed schematic timeline of the methods of stimulating **2** and **S4** under different conditions.

S4.2 ^{1}H NMR analyses of S4

As observed through ^{1}H NMR analyses after the reaction, **S4** was degraded under the stronger condition of UV light (0.3 W·cm^{-2}) or HCl (0.5 M) alone as the stable condition for pyrenylsilane (**Figure S8**).

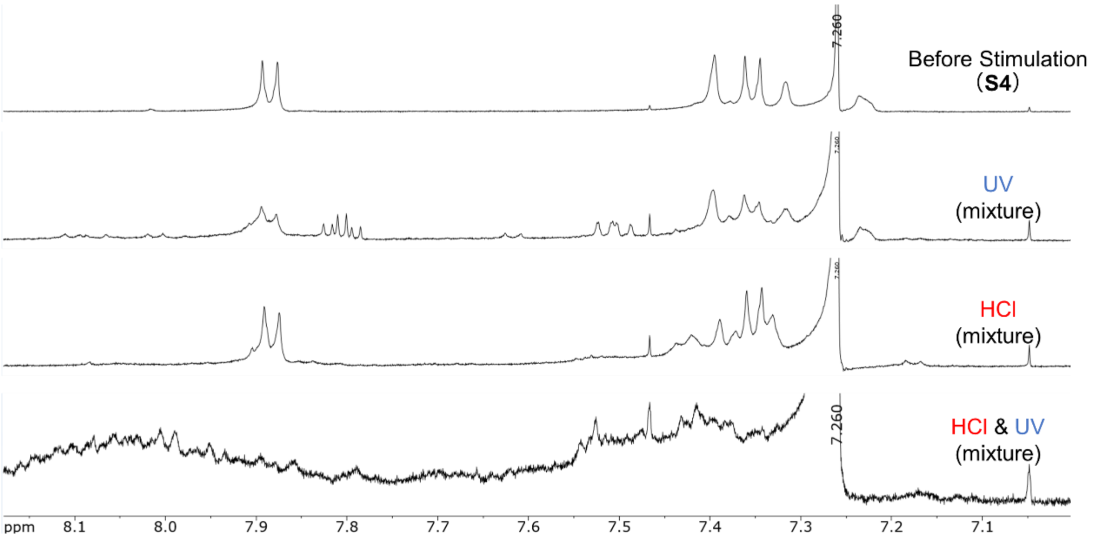

Figure S8. ^{1}H NMR spectra (500 MHz, $CDCl_3$, r.t.) recorded before and after the reaction of **S4** under three different stimuli for 30 min (UV: 365 nm, 0.3 W·cm^{-2}; HCl: 0.5 M HCl).

S4.3 SEC and ^{1}H NMR analyses of 2

As observed through SEC and ^{1}H NMR analyses after the reaction, **2** was synergistically cleaved into **3** when exposed to HCl and UV light (**Figure 2b**, **Figure S9–S12**). In contrast, **2** was stable when exposed to HCl

or UV light alone. Furthermore, the synergistic reactions proceeded even under an anaerobic atmosphere or in the presence of TEMPO radical as a radical scavenger (**Figure S13–S15**).

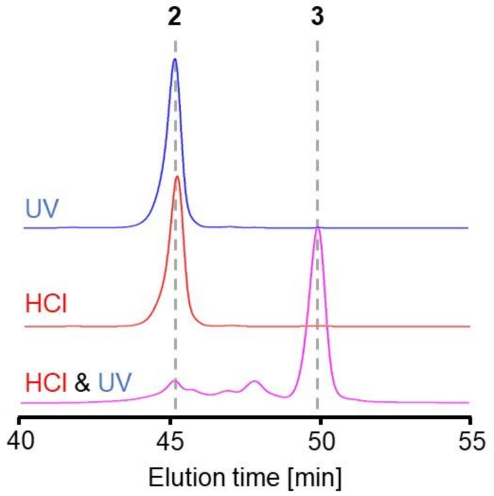

Figure S9. SEC results for **2** to **3** after three types of stimuli (SEC eluent: THF, detection wavelength: 365 nm).

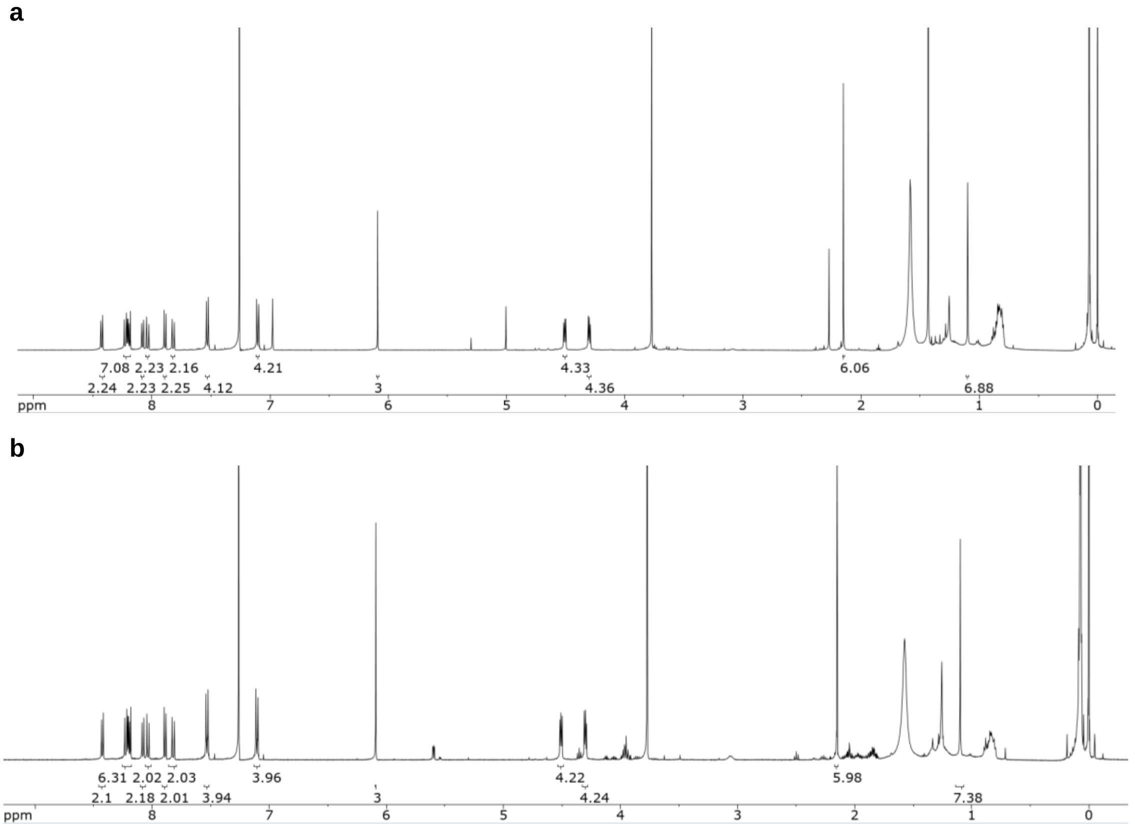

Figure S10. ^{1}H NMR spectra recorded after the reaction of **2** under the stimulation with UV light as (**a**) the standard condition (0.3 W·cm^{-2}) and (**b**) the stronger condition (0.8 W·cm^{-2}) (500 MHz, $CDCl_3$, r.t.).

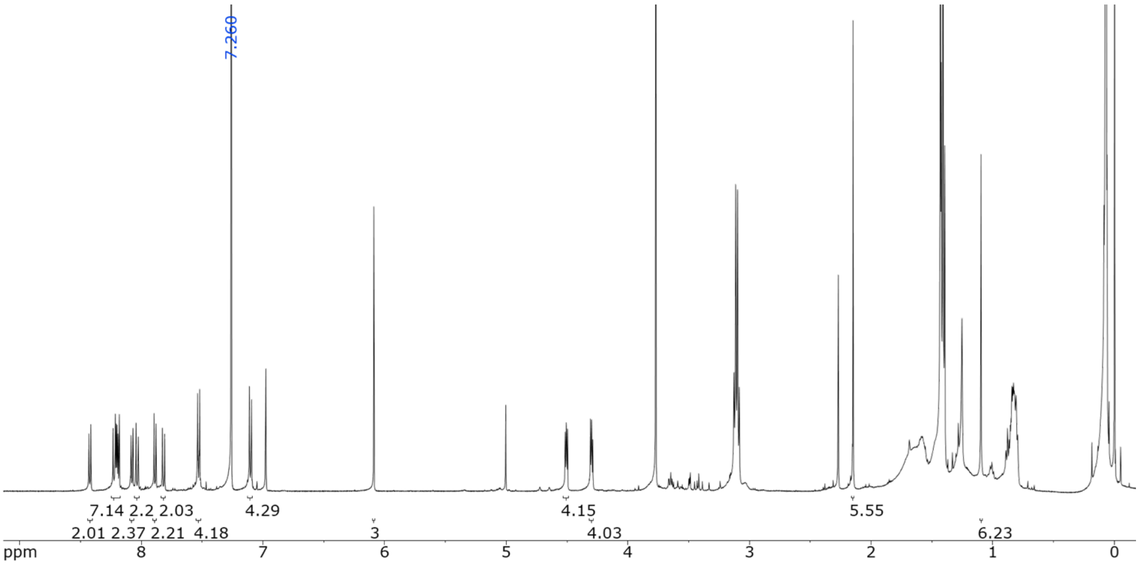

Figure S11. ^{1}H NMR spectra recorded after the reaction of **2** under the stimulation with HCl (500 MHz, $CDCl_3$, r.t.).

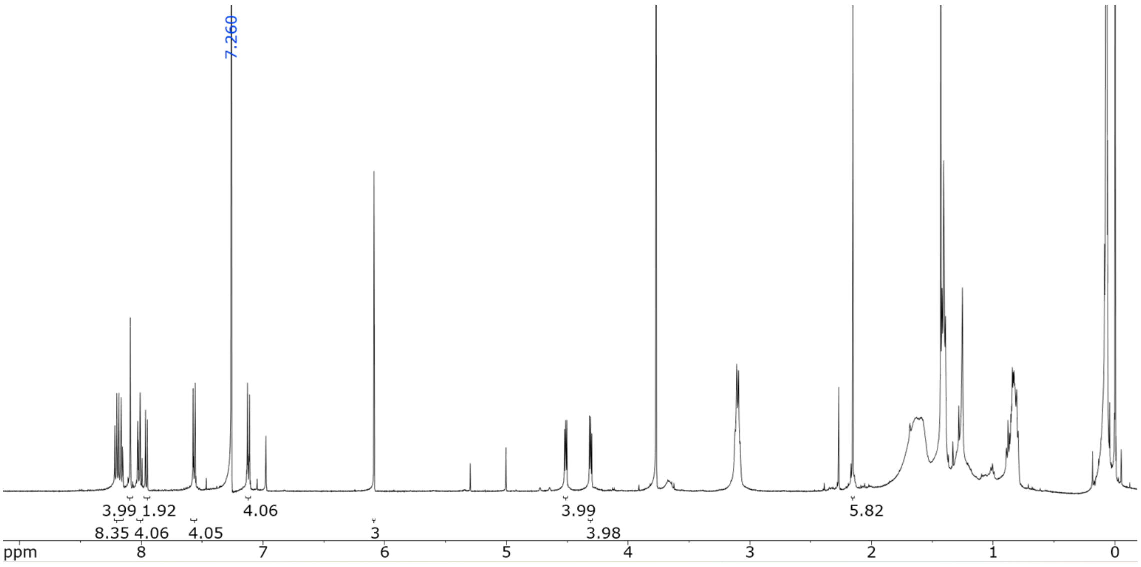

Figure S12. ^{1}H NMR spectra recorded after the reaction of **2** under the stimulation with HCl and UV light (500 MHz, $CDCl_3$, r.t.).

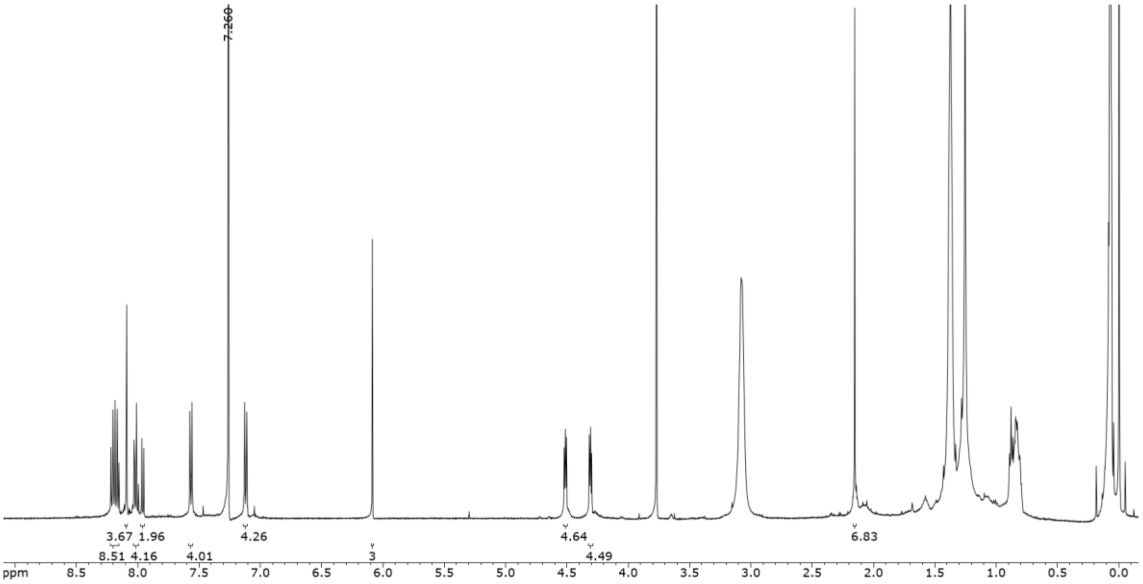

Figure S13. ¹H NMR spectra recorded after the reaction of **2** under the stimulation with HCl and UV light in the anaerobic atmosphere (500 MHz, $CDCl_3$, r.t.).

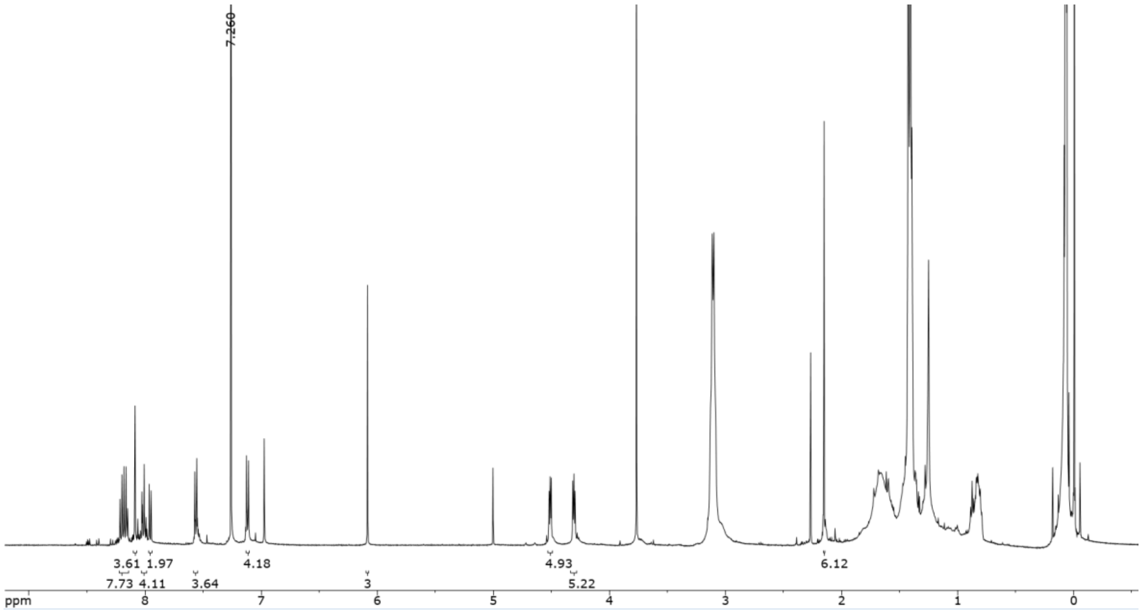

Figure S14. ¹H NMR spectra recorded after the reaction of **2** under the stimulation with HCl and UV light in the presence of TEMPO radical (500 MHz, $CDCl_3$, r.t.).

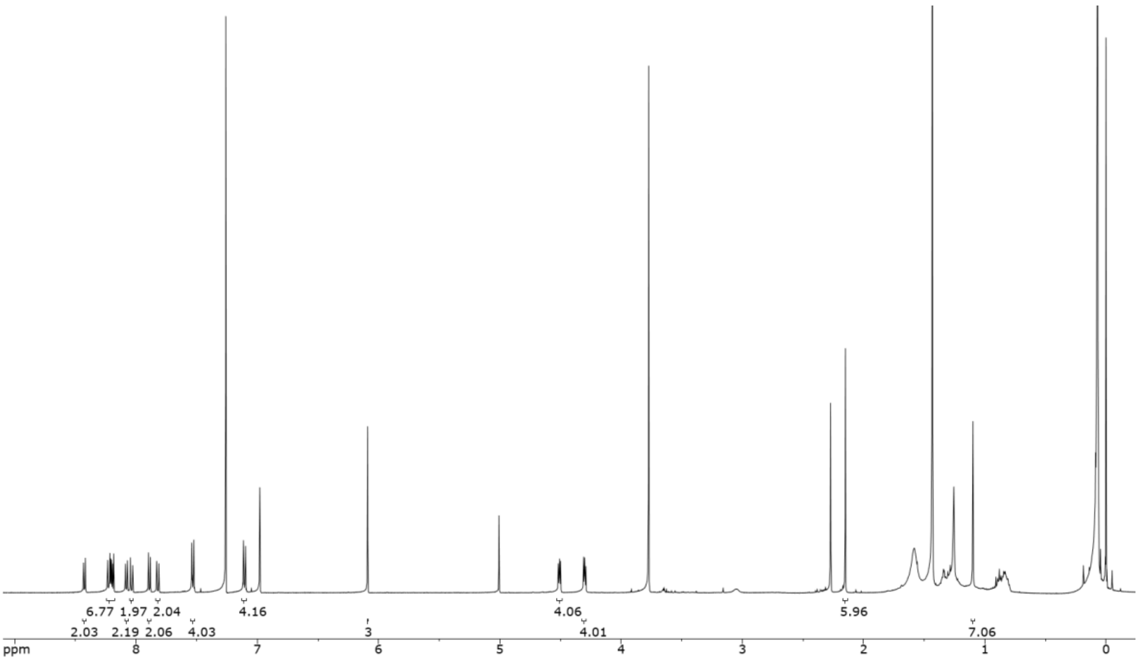

Figure S15. ^{1}H NMR spectra recorded after the reaction of **2** under the stimulation with UV light in the presence of TEMPO radical (500 MHz, $CDCl_3$, r.t.).

To further investigate the stability of compound **2**, the stimuli responsiveness of **2** under harsh UV light or acidic conditions as compared to standard conditions was measured through ^{1}H NMR measurements. Compound **2** was slightly degradable even under harsh UV light (2.4 W·cm^{-2}, 60 min) or acidic (2 M) conditions alone (**Figure S16, S17**), while **2** was completely stable up to UV light (0.8 W·cm^{-2}, 30 min) or HCl (0.5 M) conditions alone.

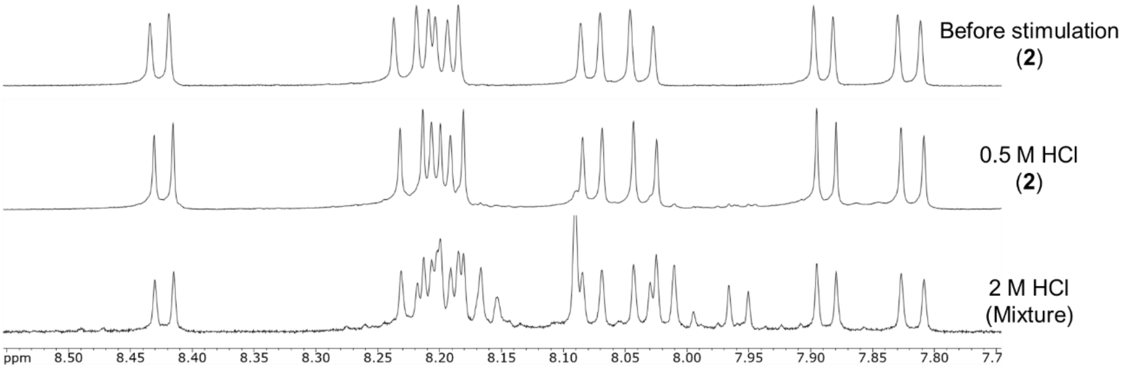

Figure S16. ^{1}H NMR spectra recorded before and after the reaction of **2** under the stimulation of HCl for 30 min in standard and harsher conditions (500 MHz, $CDCl_3$, r.t.).

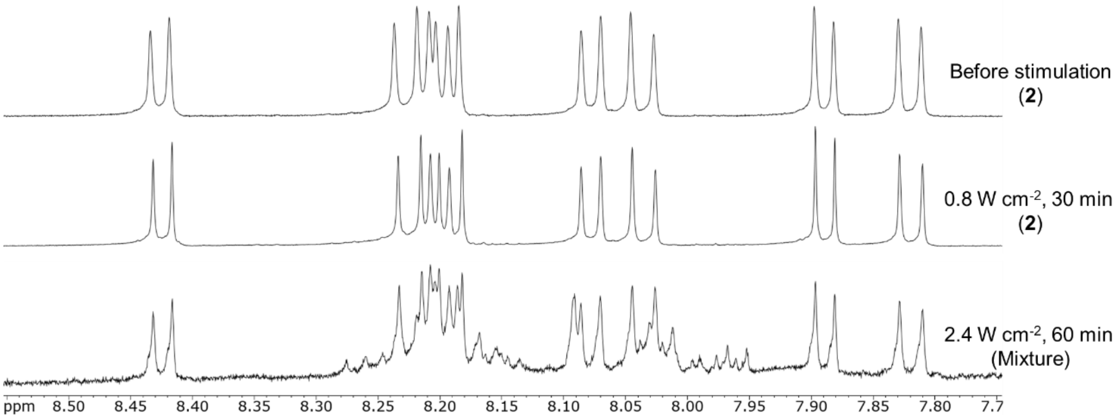

Figure S17. ^{1}H NMR spectra recorded before and after the reaction of **2** under the stimulation of UV light in standard and harsher conditions (500 MHz, $CDCl_3$, r.t.).

S4.4. Investigation of the effects of different HCl concentrations, intensities of UV light, and wavelengths of light exposure

General procedure

A solution of **2** in the reaction solvent was exposed to stimulus for 30 min, as shown below. Then, the reaction was quenched, and the residue was dissolved in THF with pyrene for SEC analysis as an internal standard. The conversion of **2** was quantified by a percentage decrease in the area ratio between the region of **2** and that of pyrene before and after the reaction.

Investigation for different HCl concentration

A solution of **2** (1.04 μmol) in HCl solution of each concentration (0.01–0.5 M) in EtOAc (2.2 mL) was exposed to UV light (λ = 365 nm, 0.3 W·cm^{-2}). After the reaction, 500 μL of Et_3N was added to the solution at 0 °C, and the solution was evaporated. The residue was dissolved in THF with pyrene (0.43 mg) for SEC analysis.

Investigation for the varying intensities of UV light

A solution of **2** (1.04 μmol) in 0.5 M HCl solution in EtOAc (2.2 mL) and exposed to UV light (λ = 365 nm, 0.02–0.3 W·cm^{-2}) at each intensity. After the reaction, 500 μL of Et_3N was added to the solution at 0 °C, and the solution was evaporated. The residue was dissolved in THF with pyrene (0.43 mg) for SEC analysis.

Investigation for varying wavelength of light exposure

A solution of **2** (0.59 μmol) in 0.5 M HCl solution in EtOAc (1.5 mL) and exposed to light with various wavelengths (365–470 nm, Asahi Spectra, CL-1503, 17 mm in diameter, approximately 0.017 W·cm^{-2}). After the reaction, brine, water, and pyrene (0.20 mg) were added to the solution. 100 μL of solution extracted from the organic layer was dissolved in approximately 2 mL THF for SEC analysis.

a

HCl concentration[M]	Conv. [%]
0.01	15
0.05	46
0.1	61
0.2	87
0.5	>95

b

UV intensity [W·cm^{-2}]	Conv. [%]
0.02	30
0.08	56
0.14	88
0.23	>95
0.30	>95

c

Wavelength [nm]	Conv. [%]
365	38
385	41
395	27
405	12
430	6
470	<5

Figure S18. Summary of conversion of **2** under (**a**) various HCl concentrations (0.01–0.5 M) with UV light (365 nm, 0.3 W·cm^{-2}), (**b**) various UV light intensities (365 nm, 0.02–0.3 W·cm^{-2}) with HCl (0.5 M), and (**c**) various wavelength of light exposure (365–470 nm, approximately 0.017 W·cm^{-2}) with HCl (0.5 M).

S5. Synergistic photoreactivity of gel materials with acid

S5.1 General procedures of macroscopic processing

The reactivity of gels under various stimuli was evaluated according to the reaction procedure shown in **Figure S19**. Disc-shaped samples (**G1** or **G7**, 9 mm in diameter, 0.4–0.5 mm in thickness) were cut from gels swollen in DMF. The samples were shrunk in MeOH and vacuumed to remove the original solvents in gels. Subsequently, the dried samples were immersed in the solvent according to reaction conditions for 3 h, to introduce the reaction solvent in gels. The gels were stimulated for partial degradation and swelling measurement. After the reaction, the gels were washed with EtOAc and MeOH and dried in a vacuum overnight. The dried samples were immersed in DMF and reswollen as the original solvent of gels (**G1** or **G7**).

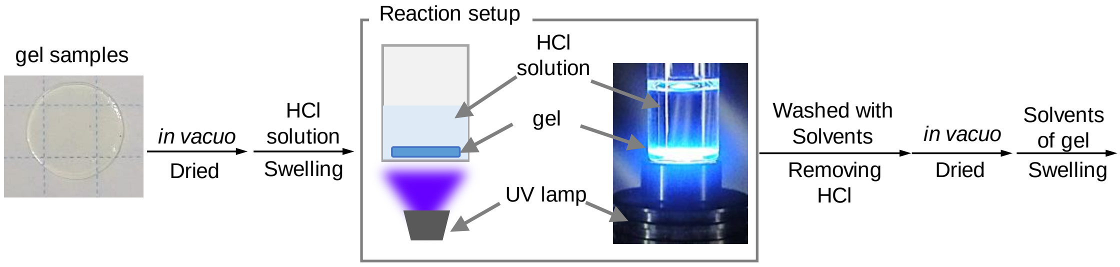

Figure S19. A detailed schematic procedure of photoprocessing for gel samples.

S5.2 Partial degradation of G1

For evaluating the macroscopic change of **G1**, the gels were exposed to stimulus as shown below (UV or HCl & UV) for 10 min. After the stimulation, the solvent of the gels was replaced to the original solvent (DMF) for the analyses (**Figure S20**).

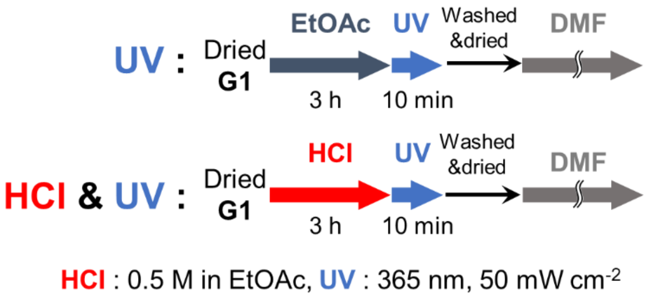

Figure S20. A detailed schematic timeline of the methods of stimulating **G1** under different conditions.

Stimulation with UV light (noted as UV)

The **G1** samples swollen in EtOAc were irradiated with UV light (365 nm, 50 mW·cm^{-2}) for 10 min in the center of the samples.

Stimulation with UV light and HCl (noted as HCl & UV)

The **G1** samples swollen in EtOAc with 0.5 M HCl were irradiated with UV light (365 nm, 50 mW·cm^{-2}) for 10 min in the center of the samples.

S5.3 Micropatterning of G1

For micropatterning of **G1**, the samples swollen in EtOAc with 0.5 M HCl were irradiated with UV light (365 nm, 80 mW·cm^{-2}) through a dotted photomask (**Figure S21a**) for 10 min. The gel was partially degraded in the areas exposed to UV light through the photomask (**Figure S21b**).

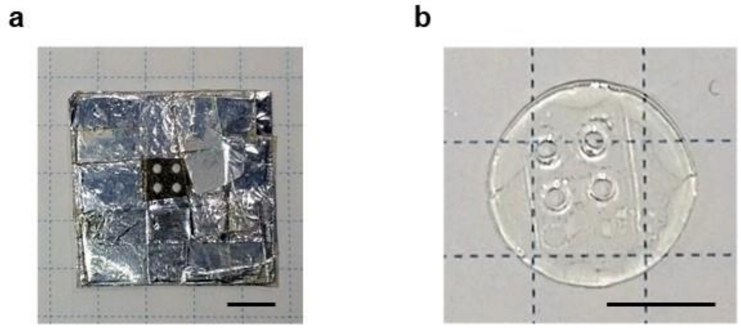

Figure S21. (**a**) The photograph of photomask for micropatterning. The distance between dots was 1 mm. (**b**) The photograph of micropatterned **G1**. Scale bar: 5 mm.

S5.4. Microlithography of G1

Square-shaped gels (**G1′**, 0.4–0.5 mm in thickness) samples were immersed in EtOAc with 0.2 M HCl for 2 h. A soda-lime glass with a 0.15 mm thickness was placed over the gels, and a line-patterned photomask made through the vapor-deposition of chromium and gold on quartz glass was placed on top of the glass (**Figure S22a**). The sample was irradiated with UV light (365 nm, 15 mW·cm^{-2}) for 1 min, and the surface of the micropatterned gel was observed with a microscope (**Figure S22b**).

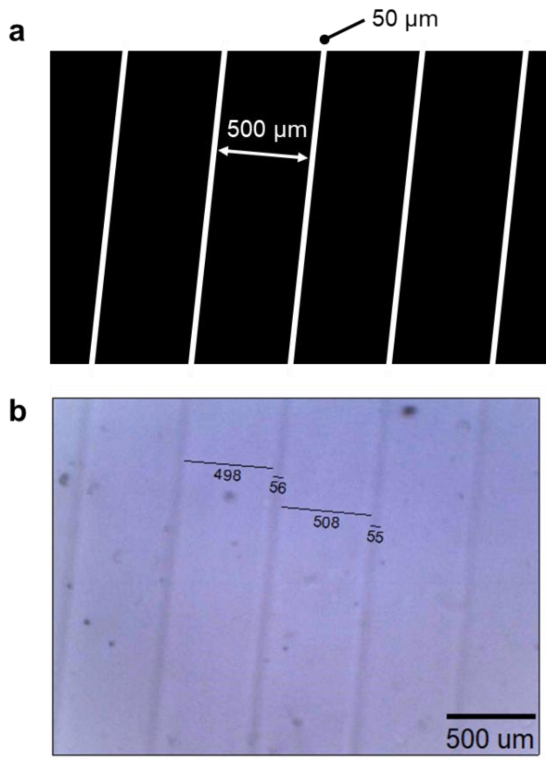

Figure S22. (**a**) The illustration of a photomask with line pattern. (**b**) Microscopy image of **G1′** after exposure to 365-nm UV light (15 mW·cm^{-2}) with HCl (0.2 M) through a photomask. The scale in the figure is in µm.

S5.5 Swelling of G7

To quantitatively evaluate the reactivity of gels with light and acid, the ratio of swelling degree before and after the reaction (S/S_0) was measured. Here, the swelling degree (S) was calculated as the ratio of the mass of the gel in the swollen state to the mass of the gel in the dried state. Before the reaction, the initial swelling degree (S_0) of **G7** in DMF was measured. After the reaction, the solvent of the gels was replaced to the original solvent (DMF). The swelling degree after the reaction (S) was measured to calculate the ratio of swelling degree (S/S_0) (**Figure S23**). Each experiment was conducted three times; the data and error bars represent averages and standard error, respectively.

As a result, S/S_0 of **G7** irradiated with UV light in the presence of HCl substantially increased to 2.3 ± 0.2 via cleaving the crosslinking points by synergistic stimulation, while S/S_0 of **G7** exposed to UV light alone or HCl alone were almost constant (1.06 ± 0.1 or 1.07 ± 0.2, respectively) (**Figure S24**).

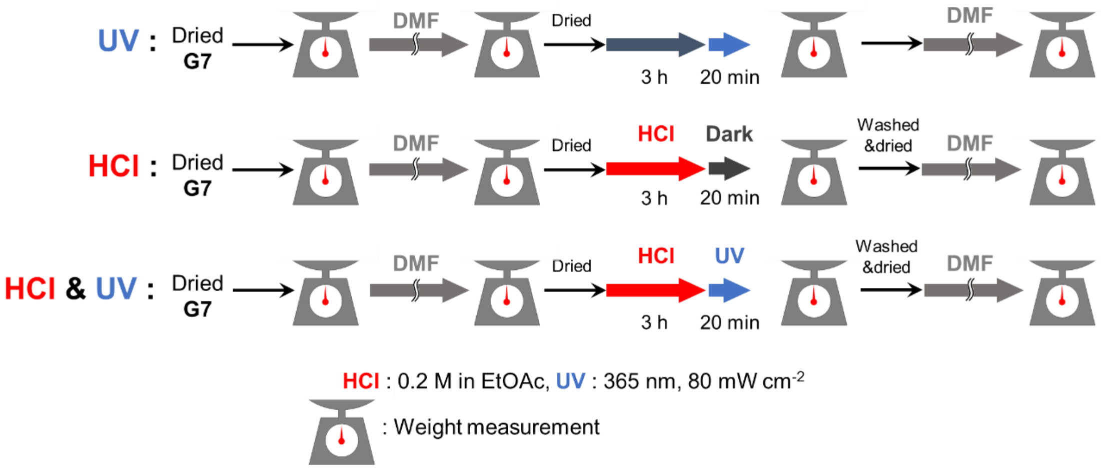

Figure S23. A detailed schematic timeline of the methods of stimulating **G7** under different conditions.

Stimulation with UV light (noted as UV)

To subject **G7** to UV light, dried **G7** samples were immersed in EtOAc for 3 h and exposed to UV light (365 nm, 80 mW·cm^{-2}) for 20 min.

Stimulation with HCl (noted as HCl)

To subject **G7** to HCl, dried **G7** samples were immersed into 0.2 M HCl solution in EtOAc for 3 h. The sample vials were then immersed at 35 °C for 20 min.

Stimulation with HCl and UV light (noted as HCl & UV)

To subject **G7** to combined HCl and UV light, dried **G7** samples were immersed in 0.2 M HCl solution in EtOAc for 3 h. The gels were then exposed to UV light (365 nm, 80 mW·cm^{-2}) for 20 min.

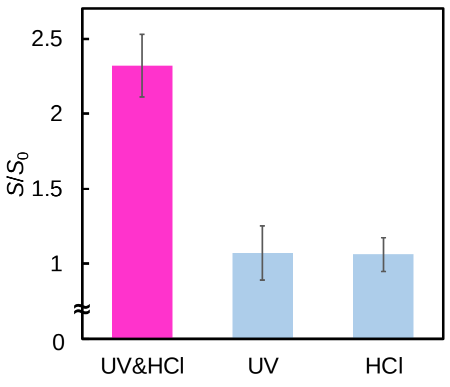

Figure S24. Comparison of the ratio of swelling degree before and after the stimuli (S/S_0).

S5.6 Photostability of G1 on white fluorescent light

The photostability of **G1** after photoprocess was examined under exposure to 20 W white fluorescent light (**Figure S26a**) in the presence or absence of HCl. Gel samples, which were micropatterned with a photomask, were swelled in DMF/EtOAc (19/1, v/v) with or without 0.2 M HCl and exposed to the fluorescent light at a

distance of 5 cm (noted as HCl & Light, Light, respectively). The mass of the gels was measured during the stimulation and was normalized by the initial mass as the mass ratio (normalized weight) (**Figure S21**). Each experiment was conducted three times; the data and error bars represent averages and standard error, respectively.

The photostability of **G1** under acidic conditions was further examined under exposure to 20 W white fluorescent light in the presence of HCl (0.01 M or 0.05 M). Gels were swelled in DMF/EtOAc (19/1, v/v) with 0.01 M or 0.05 M HCl and the time course of weight was measured through abovementioned procedure. Even under mildly acidic conditions below 0.1 M, this material gradually degraded and swelled during photoirradiation.

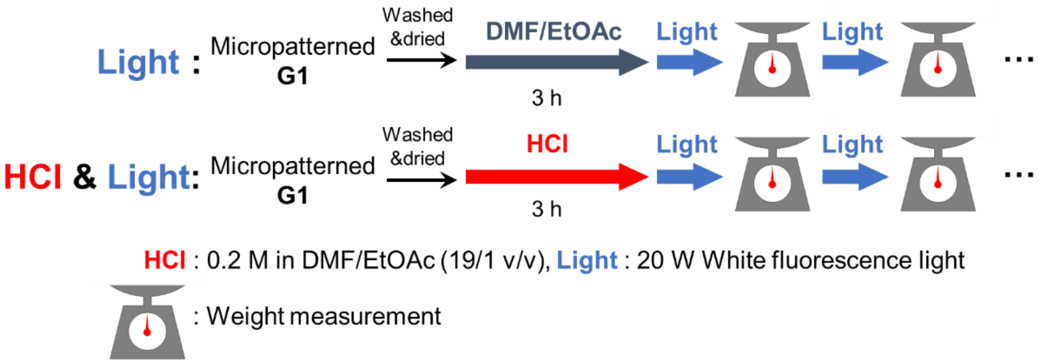

Figure S25. A detailed schematic timeline of the methods of stimulating **G7** under different conditions.

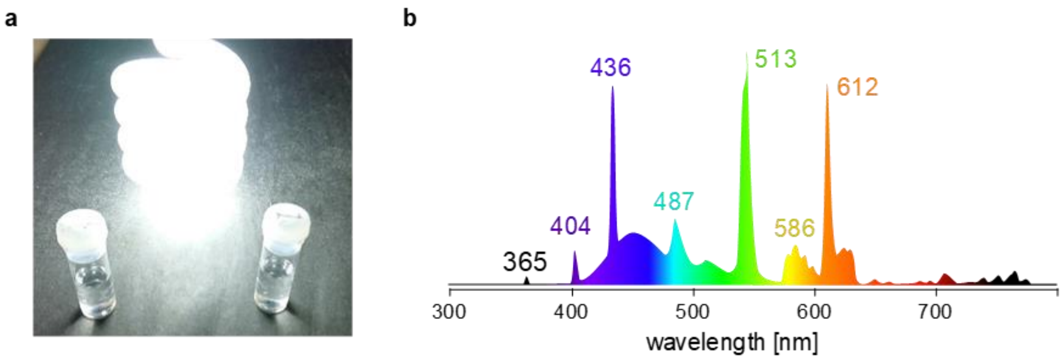

Figure S26. (**a**) Setup for the white fluorescent light irradiation to dot-shaped photoprocessed samples in DMF/EtOAc (19/1, v/v) with or without HCl (0.2 M) at a distance of 5 cm. (**b**) The luminescence spectrum of the white fluorescent light.

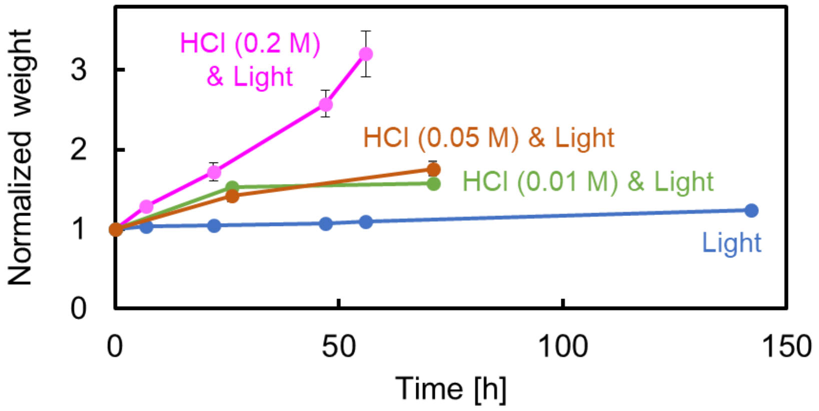

Figure S27. Time-course of the normalized weight of gel samples in fluorescent light (20 W) in the absence (blue) and presence of HCl (0.01 M, 0,05 M, and 0.2 M; green, brown, and pink, respectively) in DMF/EtOAc (19/1, v/v).

S5.7 Rheological measurements on photoprocessing of G1, G8, G9, and G10

To assess the synergistic reactivity of **G1**, **G8**, **G9**, and **G10**, the storage modulus *G′* of the gels (14 mm in diameter, 0.6–0.8 mm in thickness) during photoirradiation was measured (**Figure S28**). Additionally, the rheological changes of **G1** samples induced by photoreaction were investigated as a function of HCl concentration, UV intensity, crosslinker ratio (ν), and acidic reagents. As a standard condition, after holding the gel under an initial force (1.5 N for **G1**; 0.5 N for **G8**, **G9**, and **G10**) the storage modulus (*G′*) of gel samples in DMF/EtOAc (19/1, v/v) were measured under a constant gap while exposed to UV light (λ = 365 nm, 2 mW·cm^{-2}) in the presence or absence of HCl (0.2 M). In the case of changing acidic reagents, gel samples were swelled in DMF/H_2O (99.7/0.3, v/v) with acidic reagents (0.2 M). UV irradiation was started 60 s after the start of the rheology measurement except for the periodic ON-OFF experiment of UV irradiation. For the periodic ON-OFF experiment of UV irradiation, UV irradiation was started 120 s after the start of the rheology measurement and the gel sample was exposed to UV light discontinuously by shuttering the light (120 s with UV light on and 120 s with UV light off).

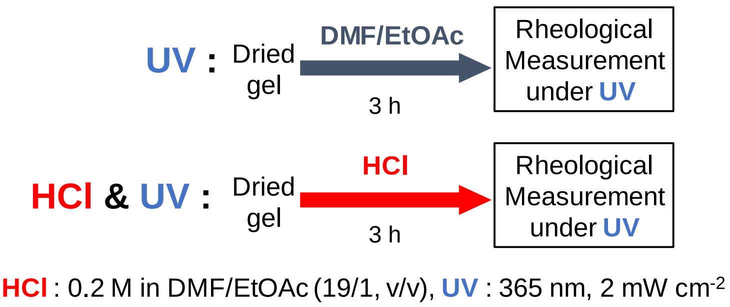

Figure S28. A detailed schematic timeline of rheological measurements on photoprocessing of gels.

Investigation of the rheological changes induced by photoreaction as a function of HCl concentration, UV intensity, crosslinker ratio (ν)

The HCl concentration was varied between 0 M and 0.2 M upon UV light irradiation (2 mW·cm^{-2}), accelerating the photoreaction with increasing the HCl concentration (**Figure S29a**). Likewise, varying the intensity of UV light (0–2 mW·cm^{-2}) in the presence of HCl (0.2 M) revealed to enhance the reactivity with increasing the UV light intensity (**Figure S29b**). In addition, the time-evolutions of G' under cooperative stimulus depended on the crosslinking ratios (ν = 0.03–0.15 mol%) of the fabricated polymer networks; Increasing the ν deteriorated the relative response ratio on G' of the materials (**Figure S29c**). Nevertheless, by prolonged light irradiation, G' was ultimately decreased to ~100 Pa even in the highly crosslinked material (ν = 0.15 mol%, **Figure S30a**). The result supported the evolution of G' was attributed to the cleavage of the pyrenylsilane-based crosslinker in the network materials.

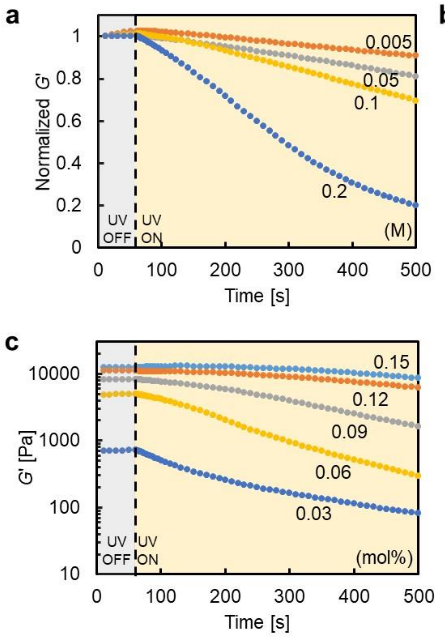

Figure S29. (**a, b**) Evolution of normalized G' during processing of **G1** under (**a**) various HCl concentrations (0.005–0.2 M) with UV light, and (**b**) various UV intensities (0.1–2 mW·cm^{-2}) with HCl. (**c**) Evaluation of G' under UV light irradiation with HCl to **G1** bearing different crosslinker ratio (0.03–0.15 mol% for fabrication). As standard conditions, 365 nm UV light (2 mW·cm^{-2}), HCl (0.2 M), and crosslinker ratio (0.09 mol% for fabrication) were utilized.

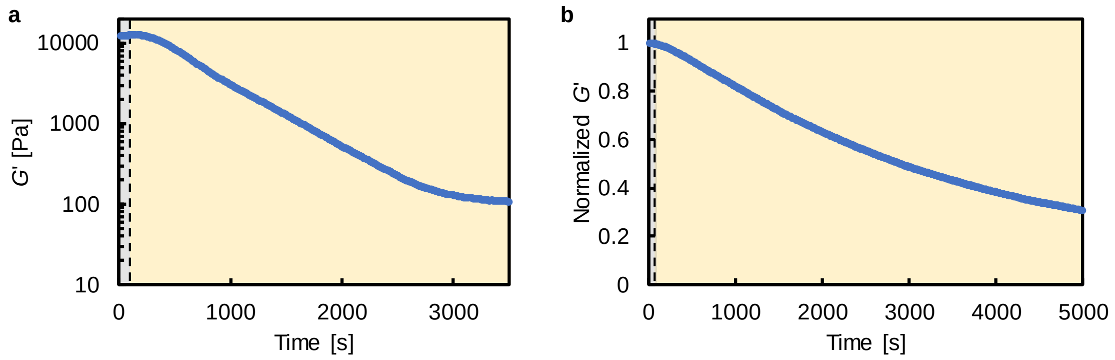

Figure S30. Evolution of *G′* of **G1** during photo-irradiation (λ = 365 nm, 2 mW·cm^{-2}). (**a**) **G1** (ν = 0.15 mol%) under HCl (0.2 M, DMF/EtOAc = 19/1, v/v). (**b**) **G1** (ν = 0.09 mol%) under acetic acid (0.2 M, DMF/H_2O = 99.7/0.3, v/v). The irradiation started at 60 s after the start of rheology measurement. Even in the high crosslinking density (ν = 0.15 mol%) or weak acidic reagent (acetic acid), *G′* was ultimately decreased to below 20% by the prolonged photo-irradiation.

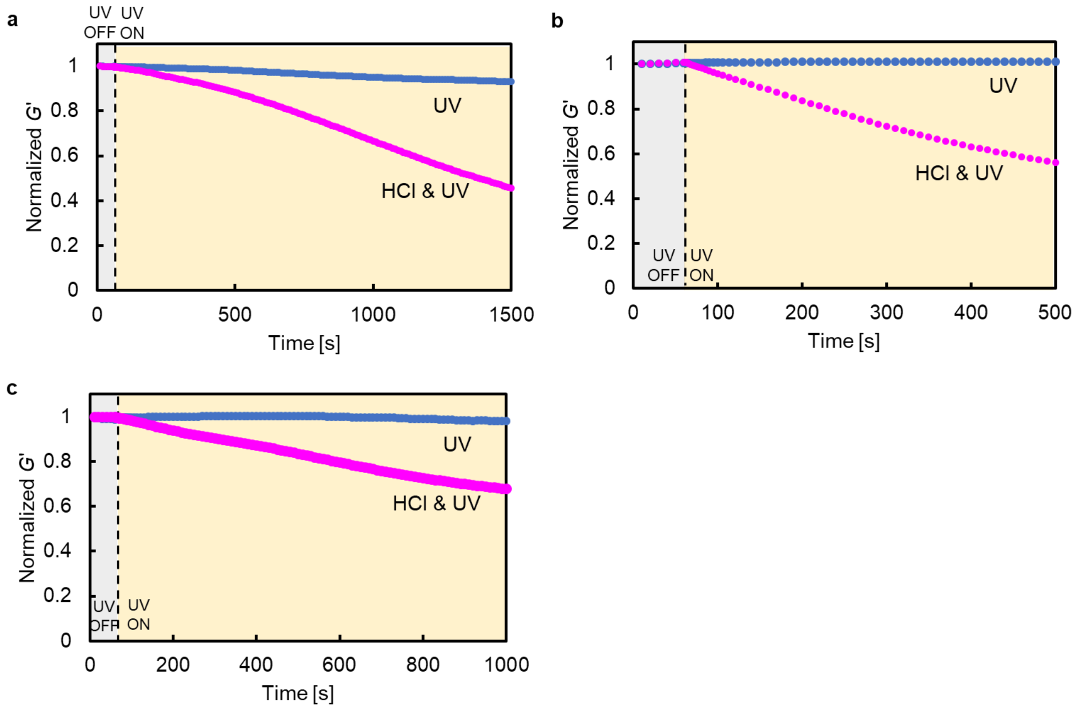

Figure S31. (**a**) Evolution of the normalized *G′* of **G8** during photo-irradiation. (**b**) Evolution of the normalized *G′* of **G9** during photo-irradiation. (**c**) Evolution of the normalized *G′* of **G10** during photo-irradiation. (Standard condition: UV (λ = 365 nm, 2 mW·cm^{-2}) HCl (0.2 M, DMF/EtOAc = 19/1, v/v) The irradiation started at 60 s after the start of rheology measurement. All gels exhibited a decrease in *G′* rapidly from the moment of UV irradiation in the presence of acid. In contrast, the *G′* of each gels were almost constant in the absence of HCl during the photoirradiation.

S6. Luminescence of gel materials

S6.1 Luminescence of micropatterned gel samples

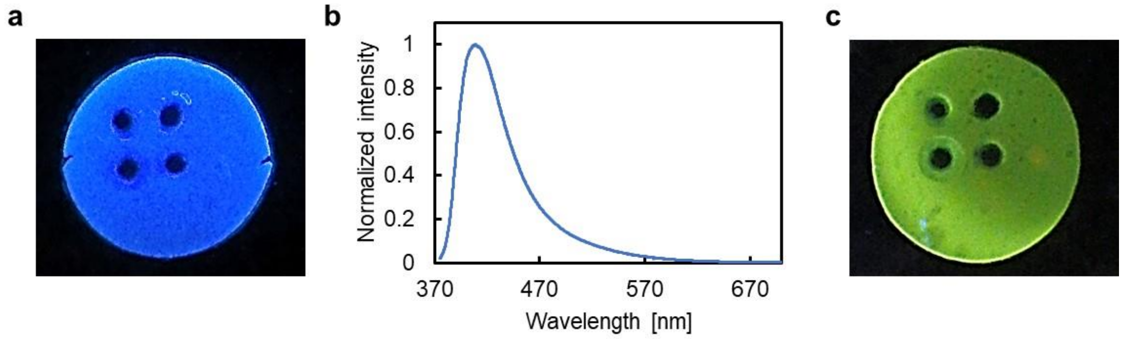

Figure S32. (**a**) Blue luminescence of micropatterned **G1** sample under 365 nm excitation. (**b**) The spectra of normalized luminescence intensity for **G1**. (Excitation = 365 nm) (**c**) Yellow luminescence of micropatterned **G5** sample under 365 nm excitation.

S6.2 Synergistic photodegradation of luminescent materials

To assess the synergistic photodegradation of luminescent material, **G5** was placed in the HCl solvent (0.5 M, EtOAc), and was irradiated with a square-shaped UV light (CCS, HLDL-50U6-NWPSC, λ = 365 nm, 45 mW·cm^{-2}, 5 cm square) for 30 min.

S7. Synergistic photodegradation of robust elastomers

S7.1 Preparation of E1

The dumbbell-shaped sample (ISO 37-4) with a thickness of 0.5 mm was cut from **G1**. The cut gels were shrunk in MeOH and vacuumed at 50 °C for 24 h to obtain specimens of **E1** (a thickness was approximately 0.2 mm).

S7.2 Elongation test of E1

The gel material was shrink in MeOH and vacuumed at 50 °C for 24 h. The square-shaped specimen of **E1** was cut from the obtained film. The top and bottom edges of the specimen were attached with clip (**Figure 4d** left). The specimen was loaded with a 500 g weight (**Figure 4d** middle). One day after removing the weight and another day after removing the clips, the specimen had returned to the original size (**Figure 4d** right).

S7.3 Photostability and thermal stability of E1

To assess the photostability and thermal stability, **E1** samples were exposed to stimulus as shown below (Pristine, Heat, or Light) for 24 h. After the stimulation, **E1** samples were used for tensile testing (**Figure S33**).

Without stimulation (noted as Pristine)

E1 samples were left in dark at room temperature for 24 h under atmospheric air.

Stimulation with heating (noted as Heat)

E1 samples were placed in an oven (150 °C) for 24 h under a nitrogen atmosphere.

Stimulation with the white fluorescent light (noted as Light)

E1 samples were exposed to the white fluorescent light (20 W) for 24 h under atmospheric air.

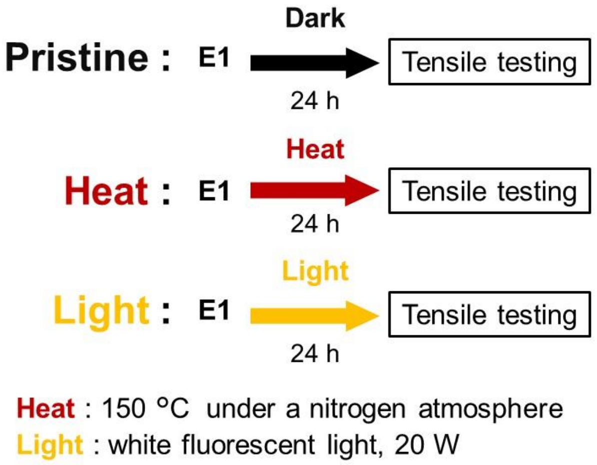

Figure S33. A detailed schematic timeline of the methods of stimulating **E1** under different conditions.

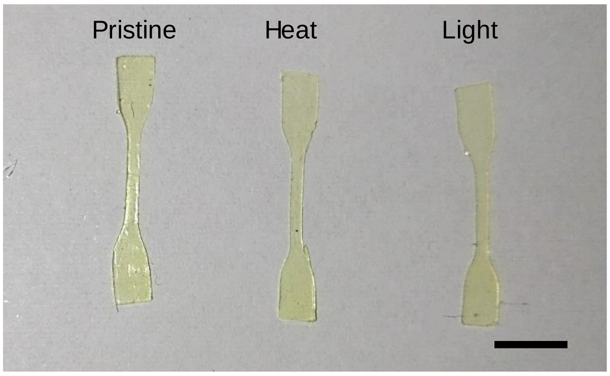

Figure S34. The photograph of pristine, heated, or light irradiated **E1**. Scale bar: 5 mm.

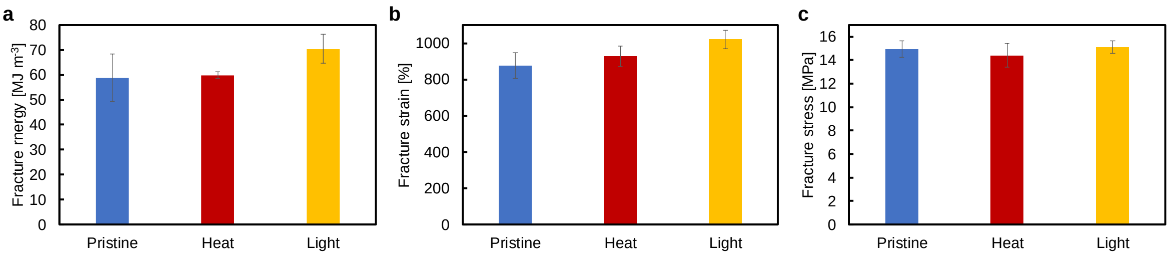

Figure S35. (**a**) Fracture energy, (**b**) Fracture stress, and (**c**) Fracture strain of pristine, heated, or light irradiated **E1**.

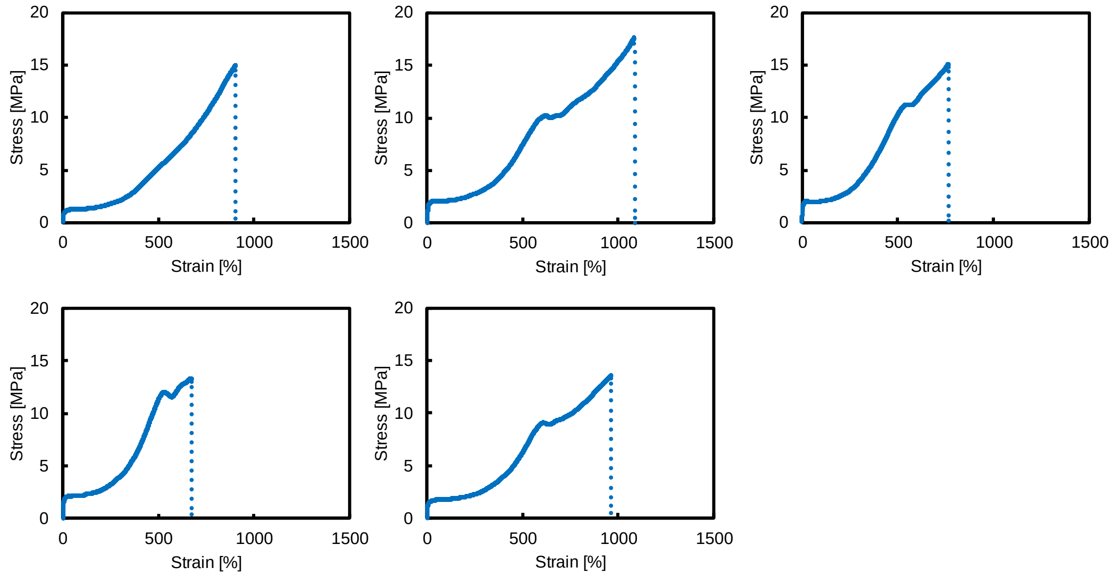

Figure S36. Stress-strain curves of five samples of pristine **E1**.

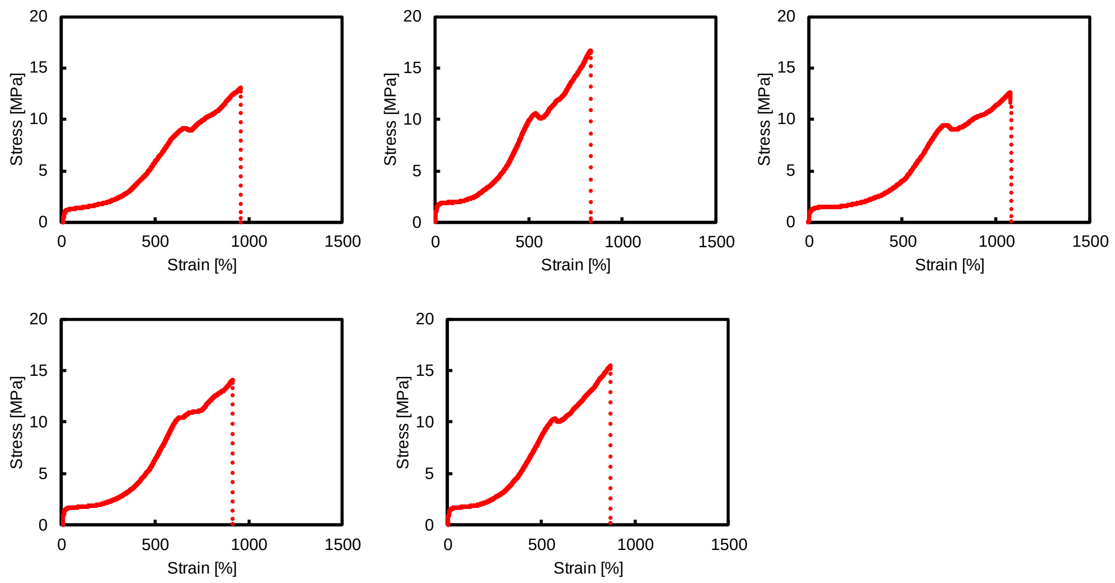

Figure S37. Stress-strain curves of five samples of heated **E1**.

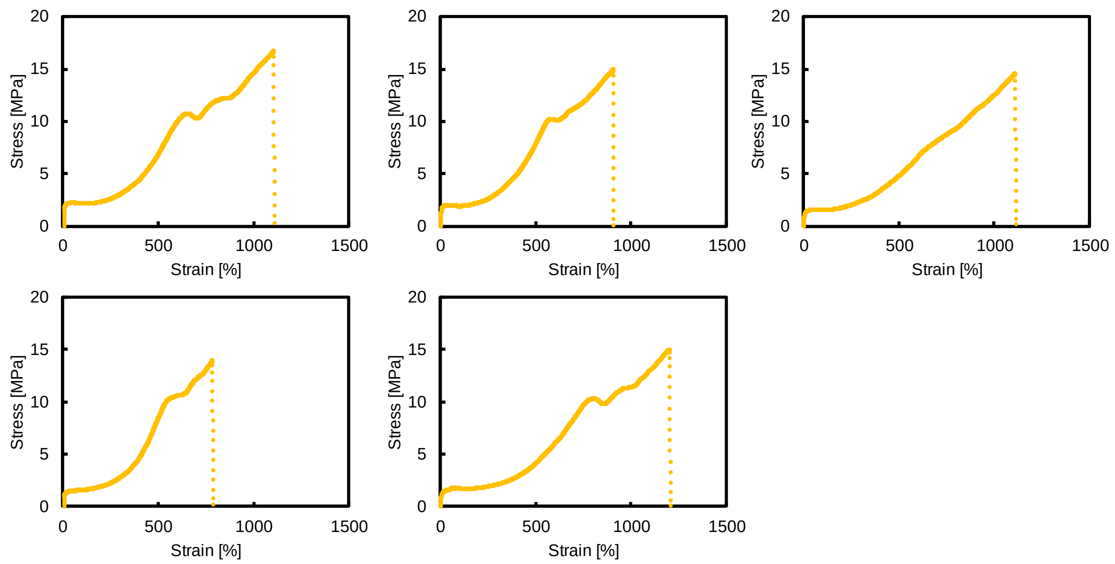

Figure S38. Stress-strain curves of five samples of light irradiated **E1**.

S7.4 Thermogravimetry analysis

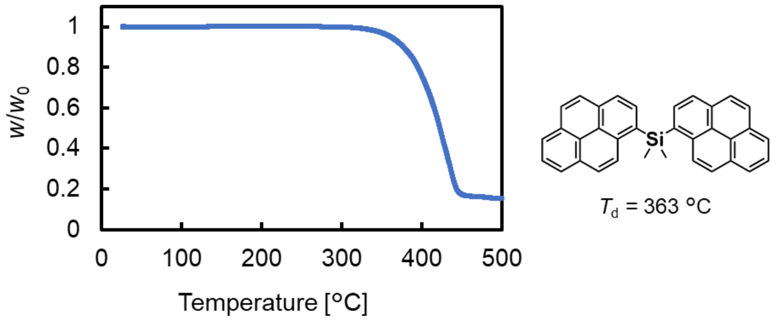

Figure S39. Thermogravimetric analysis of **1a**. The 5% weight reduction temperature was greater than 360 °C, indicating the high thermal resistance.

S7.5 Synergistic photodegradation of E1

To assess the synergistic photodegradation of elastomeric materials, **E1** was placed in the HCl solvent (0.5 M, EtOAc), and was irradiated with a square-shaped UV light (CCS, HLDL-50U6-NWPSC, λ = 365 nm, 45 $mW{\cdot}cm^{-2}$, 5 cm square) for 3 h.

S8. Synergistic photodegradation of photopolymerized materials

S8.1 Synthesis of G11 via photo-initiated radical polymerization

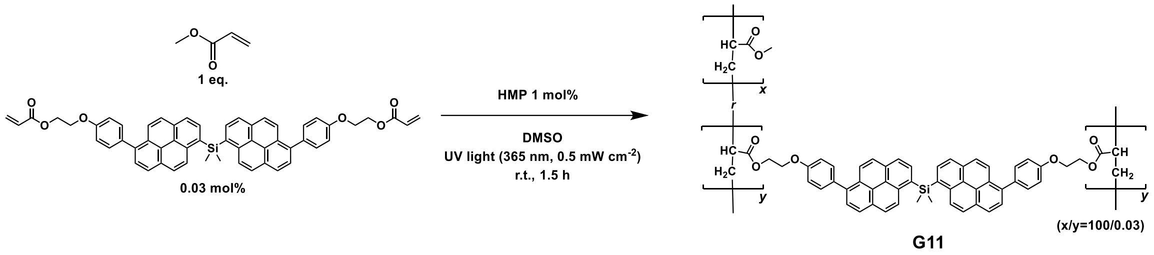

G11 was synthesized via photo-initiated radical polymerization of the corresponding monomer and crosslinker. A pregel solution containing monomers (methyl acrylate, 120 μL, 1 equiv.), crosslinkers (**8**, 0.34 mg, 0.0003 equiv.), and radical initiator (2-hydroxy-2-methylpropiophenone, 2.04 μL, 1 equiv.) in DMSO (50 μL) was degassed three times via freeze-thaw technique and was filled into the gap between two PTFE coated glass slides with a 0.3 mm PTFE thick spacer. The glass slides and spacer were held with binder clips. The reaction solution was then irradiated with two handheld UV lamps (AS ONE, LUV-16, 365 nm) from both sides for 1.5 h to carry out photopolymerization at room temperature. After polymerization, the obtained network material was washed with large amount of DMF and CH_2Cl_2, followed by shrinkage in methanol and drying in a vacuum. The dried samples were reswollen with DMF to yield **G11** samples.

S8.2 Synergistic photodegradation of G11

To assess the synergistic photodegradation of photopolymerizaed materials, after replacement of the solvent of **G11** to the reaction solvent, the gels were exposed to stimulus as shown below (UV or HCl & UV) for 10 min. After the reaction, the solvent of gels was replaced to the original solvent (DMF).

Stimulation with UV light (noted as UV)

The **G11** samples swollen in EtOAc were irradiated with UV light (365 nm, 80 mW·cm^{-2}) for 10 min in the center of the samples.

Stimulation with UV light and HCl (noted as HCl & UV)

The **G11** samples swollen in EtOAc with 0.5 M HCl were irradiated with UV light (365 nm, 80 mW·cm^{-2}) for 10 min in the center of the samples.

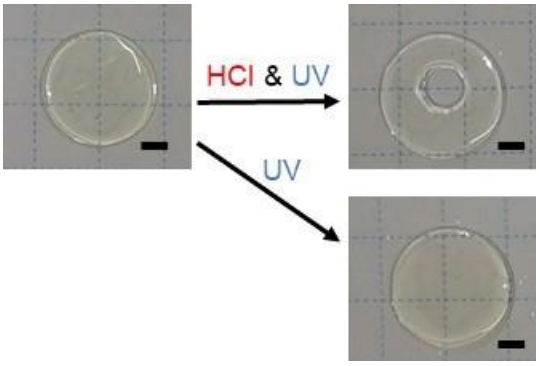

Figure S40. Photographs of synergistic photodegradation of **G11** before and after exposure of 365 nm UV light (80 mW·cm^{-2}) on the center of the gel with or without HCl (0.5 M). Scale bar: 2 mm.

S8.3 3D printing of G6

Compound **8** (1.8 mg, 2.1 μmol) and phenylbis(2,4,6-trimethylbenzoyl)phosphine oxide (BAPO) (29 mg, 69 μmol) were added to 2-phenoxyethyl acrylate (1.2 mL, 6.9 mmol) and dissolved by sonication.-The radical polymerization rate of monomer was critical for successful 3D printings; 2-Phenoxyethyl acrylate possessed fast reaction rate as the resin of 3D printing.[4] The resulting solution was used as resin for 3D printing of gel materials. 3D printing was performed on a Elegoo Mars 3 pro from Elegoo equipped with handmade vat and platform (**Figure S41**) with a layer thickness of 50 μm (405 nm LED). When printing **G6**, the first 5 layers were irradiated for 70 s to ensure adhesion to the platform and all subsequent model layers were each irradiated for 10 s. Because the 3D printing without the support resulted in the failure due to the distortion of the target structure (**Figure S42**), the supporting pillar was necessary to assist the successful 3D printing. Printed material was gently detached from the platform and washed by DMF and EtOAc with 0.5 M HCl.

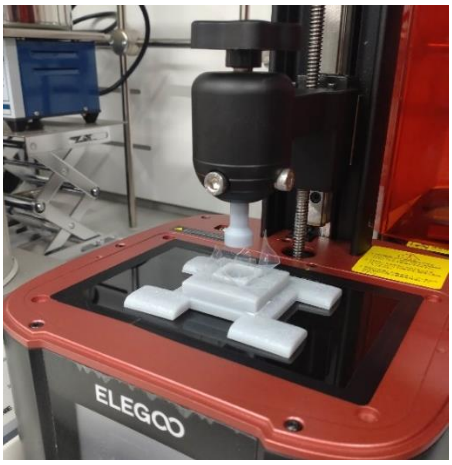

Figure S41. Photograph of the set-up used for 3D printing. The 3D printer was equipped with handmade vat and platform. The reaction solution was placed inside the vat.

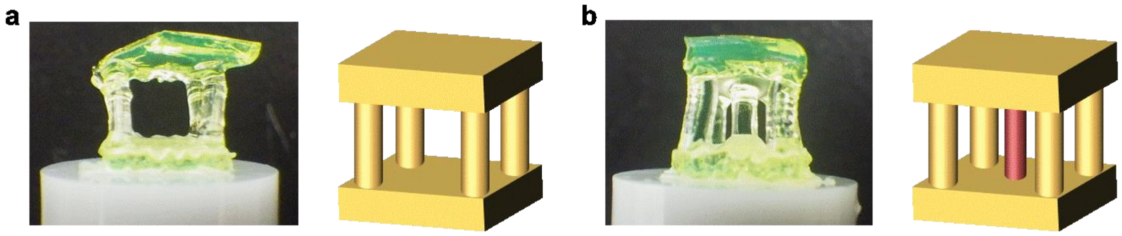

Figure S42. Photographs of the cage objects just after 3D printing (**a**) without and (**b**) with the supporting pillar.

S8.4 Photodegradation of G6

G6 was swollen in large amounts of EtOAc with 0.5 M HCl and irradiated with UV light (365 nm, 45 W·cm^{-2}) for 30 min until photo-degradation. For UV irradiation, a square-shaped UV irradiation lamp (CCS, HLDL-50U6-NWPSC, λ = 365 nm, 5 cm square) was used.

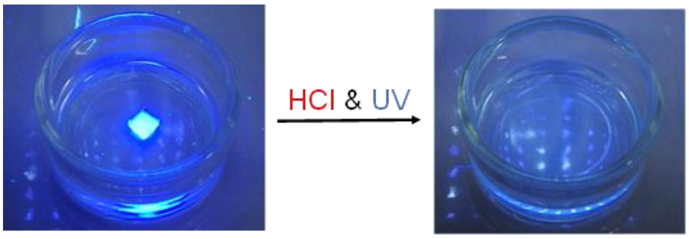

Figure S43. Photographs of **G6** before and after synergistic degradation with light and acid.

S8.5 Local photodegradation of G6

G6 was swollen in large amounts of EtOAc with 0.5 M HCl and irradiated with a spot-type UV light (CCS, 8332A-AC8361-AC8303 covered by aluminum foil with a 2 mm diameter hole, λ = 365 nm, 0.5 W·cm^{-2}) for 2–10 min until photo-degradation. After the degradation, the gel was washed with EtOAc, DMF and MeOH and dried in a vacuum overnight. The dried sample was swollen in mixed solvent of DMF and MeOH.

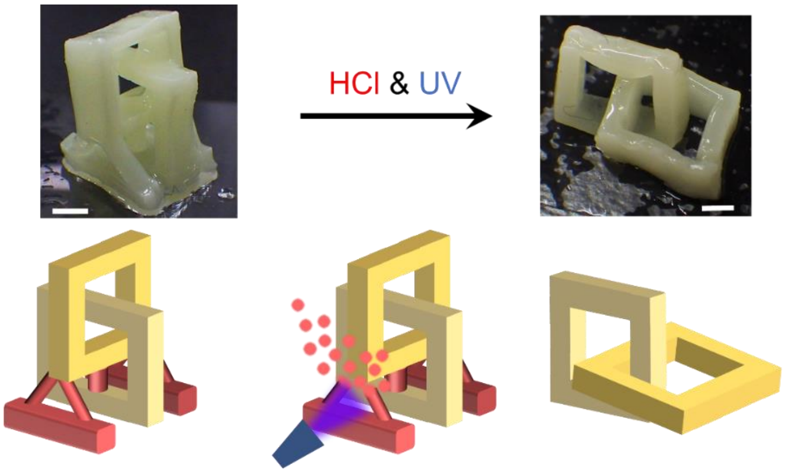

Figure S44. Photographs and schematics of 3D printed chain before and after synergistic degradation with 365 nm UV light and HCl (0.5 M) on the supporting structure, which is marked in pink in the schematic. Scale bar: 2 mm.

S9. Computational calculations

To explore the unique reactivity of **2** under cooperative stimuli with light and acid, a plausible reaction mechanism was evaluated by density functional theory (DFT) calculation with for the model reaction of pyrenylsilicon derivative (**4**) and HCl in the ground (S_0) and excited (S_1) states. All calculations on both the ground state (S_0) and the lowest-energy excited singlet state (S_1) in EtOAc were conducted using density functional theory (DFT) as implemented in Gaussian 16.[5] We selected CAM-B3LYP exchange-correlation functional with aug-cc-pVDZ (for O and Cl atoms) and cc-pVDZ (for the other atoms) as the basis set. The thermal correction to Gibbs energy at 298.15 K was calculated by the frequency calculation at the optimization condition and it was added to the electron energy. The solvation effect of EtOAc was computed with SMD solvation model[6] in all the calculations. The connections of stationary points were verified by intrinsic reaction coordinate (IRC) calculations. Optimized structures of **4**, **4*$_{vert.}$**, **A**, **TS$_{4–5}$**, **5**, **B**, **TS$_{5–6}$**, **6**, **7**, **4***, **A***, **TS$_{4–5}$***, **5*** and HCl are shown in **Table S2–S14**.

A traditional desilylation of arylsilane with HCl was involved in α-protonation on arylsilane to afford an intermediate of arenium ion (β-cation) and subsequent desilylation to yield the corresponding arene (**6**) and chlorosilane (**7**). Hence, a mixture of **4** and HCl (**A**) proceeded the protonation through the first transition state (**TS$_{4–5}$**) to form the intermediate (**5**), followed by changing the coordinate of counter-anion (**B**) before the desilylation of **5** through the second transition state (**TS$_{5–6}$**), generating the desilylated product (**6**). On the other hand, in the excited state with UV irradiation, the vertically excited structure (**4*$_{vert.}$**) rearranged the relaxed structure (**4***). The excited species partly quenched via radiative and non-radiative processes. In the presence of HCl, the mixture (**A***) proceeded the protonation through the transition state in the excited state (**TS$_{4–5}$***) to yield the excited Wheland intermediate (**5***), which was relaxed to the ground state (**5**). Finally, the intermediate **5** in the ground state led to proceed desilylative bond-cleavage reaction to yield **6**, thermally accompanying with the back-reaction from **5** to **4**.

In the ground state without UV irradiation, the two Gibbs free energies of transition states for activating protonation ($\Delta G^{\circ\ddagger}_{4–5}$) and desilylation ($\Delta G^{\circ\ddagger}_{5–6}$), were calculated as similar values of ~23 kcal mol^{-1}. In contrast, the Gibbs free energies of transition state of protonation in the excited state ($\Delta G^{*\circ\ddagger}_{4–5}$) was calculated as 12.3 kcal mol^{-1}, which is significantly lower than that in the ground state ($\Delta G^{\circ\ddagger}_{4–5}$). Moreover, the excited state led the endothermic protonation from **4** to **5** to be more favorable; the Gibbs free energies ($\Delta G^{\circ}_{4–5}$) of formation were 17.9 kcal mol^{-1} and 3.2 kcal mol^{-1} in the ground and excited states, respectively. The lower energies of activation ($\Delta G^{\circ\ddagger}_{4–5}$) and formation ($\Delta G^{\circ}_{4–5}$) in the excited pathway would be attributed to the dearomatization of pyrene moiety in the photoexcited substrate (**4***), as a preparatory state of the protonated intermediate (**5***), confirmed by the degree of bond alternation on the aromatic ring of **4***. (**Figure S40**). Accordingly, the excited state pathway decreased the activation barrier and endotherm in the protonation step of **3** and HCl to accelerate the formation of Wheland intermediate, as compared to the ground state, which contributed to the synergistic reactivity with light and acid.

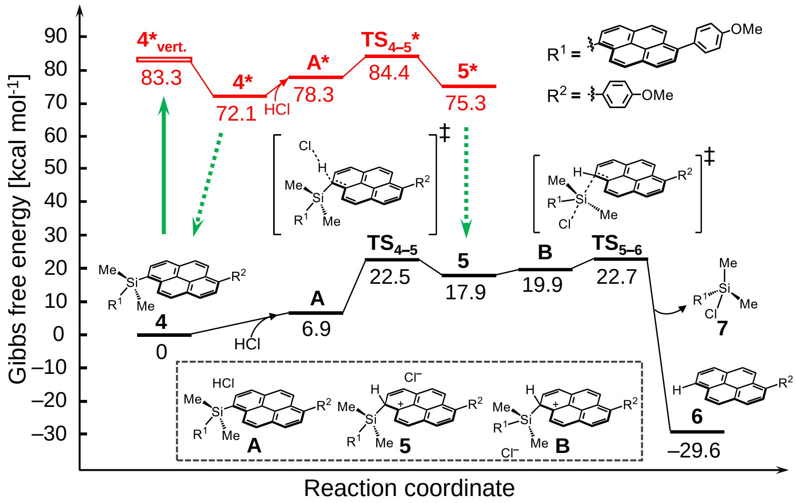

Figure S45. Energy profile for desilylation of **4** with HCl in the ground and excited states (CAM-B3LYP/cc-pVDZ (for H,C,Si); aug-cc-pVDZ (for O, Cl), SMD (EtOAc)). Green rigid arrow: photoexcitation, green dotted arrow: radiative or non-radiative processes.

Table S2. Optimized structural coordinates of **4** and **4***$_{vert.}$

Num.	Atom	Coordinates / c		
		X	Y	Z
1	Si	0.000019	4.234970	-0.000516
2	C	-5.150010	-1.426310	2.147166
3	C	-4.040329	-0.749703	2.629113
4	C	-3.397256	0.213262	1.844576
5	C	-3.908322	0.505947	0.552198
6	C	-5.056466	-0.188211	0.065015
7	C	-5.666891	-1.176688	0.870288
8	C	-2.232537	0.915779	2.306626
9	C	-3.265651	1.505882	-0.251015
10	C	-2.111967	2.196905	0.227791
11	C	-1.622797	1.851461	1.540429
12	C	-1.487146	3.185651	-0.571392
13	C	-2.034681	3.452600	-1.833940
14	C	-3.157354	2.788617	-2.311725
15	C	-3.789774	1.814363	-1.535478
16	C	-4.966171	1.124676	-1.991112
17	C	-5.568382	0.177731	-1.233501
18	H	-6.467986	-0.314204	-1.600403
19	H	-5.373680	1.390579	-2.968879
20	H	-1.842382	0.673580	3.297467
21	H	-5.623574	-2.191109	2.765277
22	H	-3.650800	-0.974619	3.624101
23	H	-0.733067	2.351766	1.921523
24	H	-1.573969	4.207913	-2.473202
25	H	-3.558142	3.027451	-3.299244
26	C	1.486640	3.185033	0.571047
27	C	2.111333	2.195916	-0.227776
28	C	2.034229	3.452297	1.833497
29	C	3.265191	1.505237	0.251042
30	C	1.621622	1.849595	-1.539975
31	C	3.157039	2.788563	2.311340
32	H	1.573559	4.207695	2.472687
33	C	3.907786	0.505056	-0.551939
34	C	3.789554	1.814273	1.535291
35	C	2.231222	0.913618	-2.305933
36	H	0.731536	2.349435	-1.920870
37	H	3.557775	3.027652	3.298817
38	C	3.396315	0.211650	-1.844002
39	C	5.056245	-0.188637	-0.064832
40	C	4.966249	1.125001	1.990825
41	H	1.840662	0.670776	-3.296455
42	C	4.039326	-0.751558	-2.628303
43	C	5.666646	-1.177301	-0.869873
44	C	5.568475	0.177931	1.233394
45	H	5.373991	1.391376	2.968368
46	C	5.149351	-1.427653	-2.146449
47	H	3.649487	-0.977049	-3.623041
48	H	6.468305	-0.313666	1.600204
49	H	5.622877	-2.192622	-2.764381
50	C	0.553937	5.347463	-1.417757
51	H	-0.236817	6.058955	-1.705131
52	H	1.424425	5.935775	-1.084232
53	H	0.858262	4.787915	-2.314983
54	C	-0.552223	5.349510	1.415654
55	H	-1.420520	5.940380	1.080892
56	H	-0.859366	4.791348	2.312786
57	H	0.240412	6.058698	1.703489
58	C	-6.843959	-1.968237	0.414409
59	C	-6.799277	-2.757706	-0.746040
60	C	-8.019267	-1.990120	1.167303
61	C	-7.885007	-3.525406	-1.135195
62	H	-5.888568	-2.782812	-1.347298
63	C	-9.123074	-2.754099	0.787147
64	H	-8.086375	-1.385107	2.073844
65	C	-9.057658	-3.526833	-0.371858
66	H	-7.841649	-4.143264	-2.033583
67	H	-10.021961	-2.733061	1.400987
68	C	6.844095	-1.968322	-0.414088
69	C	8.019040	-1.990435	-1.167538
70	C	6.800181	-2.757039	0.746909
71	C	9.123199	-2.753944	-0.787454
72	H	8.085588	-1.385968	-2.074486
73	C	7.886267	-3.524255	1.136009
74	H	5.889796	-2.781926	1.348667
75	C	9.058534	-3.525949	0.372075
76	H	10.021767	-2.733084	-1.401768
77	H	7.843502	-4.141521	2.034834
78	O	-10.071520	-4.308065	-0.834108
79	O	10.072786	-4.306676	0.834318
80	C	-11.277370	-4.345170	-0.083851
81	H	-11.743864	-3.348884	-0.020863
82	H	-11.949806	-5.024058	-0.622401
83	H	-11.107673	-4.735590	0.932473
84	C	11.278372	-4.343786	0.083634
85	H	11.951261	-5.022098	0.622345
86	H	11.108445	-4.734882	-0.932392
87	H	11.744466	-3.347363	0.019868

Table S3. Optimized structural coordinates of **A**

Num.	Atom	Coordinates / Å		
		X	Y	Z
1	Si	-0.070417	4.163191	-0.311676
2	C	5.058998	-1.569989	-2.322279
3	C	3.923162	-0.934133	-2.798273
4	C	3.294056	0.060361	-2.042200
5	C	3.845767	0.426592	-0.786299
6	C	5.021233	-0.224691	-0.306159
7	C	5.617353	-1.246443	-1.079777
8	C	2.104502	0.723134	-2.498140
9	C	3.216540	1.458026	-0.013337
10	C	2.036612	2.108712	-0.485572
11	C	1.507983	1.690005	-1.760622
12	C	1.423969	3.131636	0.281196
13	C	2.007879	3.468618	1.509333
14	C	3.160020	2.845725	1.982061
15	C	3.783874	1.841664	1.232073
16	C	4.988360	1.196394	1.678975
17	C	5.575535	0.217885	0.949980
18	H	6.496446	-0.240358	1.307317
19	H	5.428716	1.521764	2.623795
20	H	1.683443	0.424721	-3.460543
21	H	5.521488	-2.360192	-2.916156
22	H	3.501986	-1.216215	-3.765351
23	H	0.597943	2.157474	-2.134414
24	H	1.561092	4.252535	2.123516
25	H	3.602092	3.156899	2.931703
26	C	-1.567519	3.107403	-0.842523
27	C	-2.210595	2.164179	-0.003914
28	C	-2.103248	3.323605	-2.119944
29	C	-3.369301	1.465916	-0.459787
30	C	-1.736684	1.874626	1.327482
31	C	-3.230455	2.651976	-2.575650
32	H	-1.629308	4.043420	-2.789627
33	C	-4.030283	0.511746	0.383190
34	C	-3.879958	1.721148	-1.761193
35	C	-2.365397	0.984496	2.131968
36	H	-0.844691	2.382544	1.693355
37	H	-3.621225	2.850123	-3.576107
38	C	-3.533956	0.274220	1.692322
39	C	-5.181899	-0.191884	-0.081534
40	C	-5.060556	1.024295	-2.194703
41	H	-1.988777	0.787010	3.137602
42	C	-4.194329	-0.644063	2.515110
43	C	-5.809617	-1.135728	0.762985
44	C	-5.679216	0.119471	-1.399758
45	H	-5.457437	1.249746	-3.186875
46	C	-5.306849	-1.330950	2.054925
47	H	-3.815440	-0.825925	3.522925
48	H	-6.581331	-0.379078	-1.751342
49	H	-5.793693	-2.061533	2.703371
50	H	1.984350	1.214124	3.122510
51	C	-0.602548	5.327794	1.070924
52	H	0.192899	6.048430	1.319768
53	H	-1.476963	5.904735	0.727993
54	H	-0.894853	4.802858	1.992641
55	C	0.489093	5.219025	-1.768981
56	H	1.359804	5.818418	-1.456577
57	H	0.794855	4.622654	-2.641812
58	H	-0.299450	5.919639	-2.087331
59	Cl	1.501207	0.382373	4.006136
60	C	6.821035	-1.997587	-0.625758
61	C	6.829045	-2.723081	0.576537
62	C	7.968226	-2.046304	-1.419633
63	C	7.938487	-3.456005	0.965545
64	H	5.941177	-2.726436	1.211552
65	C	9.095490	-2.775322	-1.039965
66	H	7.994092	-1.490565	-2.359241
67	C	9.082570	-3.485122	0.160455
68	H	7.935926	-4.025087	1.896558
69	H	9.971119	-2.776395	-1.686934
70	C	-6.989843	-1.936753	0.332626
71	C	-8.172939	-1.912633	1.073139
72	C	-6.940518	-2.780573	-0.788725
73	C	-9.279836	-2.683979	0.717586
74	H	-8.243737	-1.264850	1.949343
75	C	-8.029231	-3.556245	-1.152864
76	H	-6.023881	-2.841896	-1.378240
77	C	-9.209596	-3.511258	-0.402864
78	H	-10.184796	-2.626638	1.320108
79	H	-7.982339	-4.216323	-2.020532
80	O	10.122474	-4.228801	0.626025
81	O	-10.225786	-4.304623	-0.838481
82	C	11.301896	-4.291377	-0.163578
83	H	11.752719	-3.294465	-0.294241
84	H	12.001409	-4.933685	0.385223
85	H	11.102121	-4.735948	-1.151764
86	C	-11.440208	-4.294270	-0.101317
87	H	-12.112919	-4.992696	-0.613921
88	H	-11.285747	-4.636010	0.934788
89	H	-11.897901	-3.291987	-0.092620

Table S4. Optimized structural coordinates of $\mathbf{TS_{4\text{–}5}}$

Num.	Atom	Coordinates / Å		
		X	Y	Z
1	Si	-0.115637	3.396653	1.127261
2	C	-4.870089	-2.782788	-0.199155
3	C	-3.510463	-2.517194	-0.189901
4	C	-3.039811	-1.199051	-0.152226
5	C	-3.974668	-0.134645	-0.101688
6	C	-5.372658	-0.411601	-0.111080
7	C	-5.819473	-1.752991	-0.178683
8	C	-1.642482	-0.892030	-0.176001
9	C	-3.500217	1.214018	-0.041474
10	C	-2.103649	1.492601	-0.027921
11	C	-1.195115	0.390511	-0.122260
12	C	-1.628193	2.854052	0.012077
13	C	-2.628906	3.881147	0.040082
14	C	-3.973370	3.617541	0.027239
15	C	-4.435529	2.284491	0.002419
16	C	-5.832568	1.983826	0.025614
17	C	-6.277785	0.700784	-0.019656
18	H	-7.348324	0.505510	0.022302
19	H	-6.540368	2.811866	0.090482
20	H	-0.931176	-1.717703	-0.245171
21	H	-5.215913	-3.815924	-0.254281
22	H	-2.791969	-3.338422	-0.228409
23	H	-0.127158	0.592537	-0.159703
24	H	-2.302453	4.924038	0.049197
25	H	-4.700763	4.430896	0.037615
26	C	1.269469	2.104739	1.333008
27	C	2.342268	1.882752	0.432831
28	C	1.252057	1.367308	2.526800
29	C	3.369238	0.943125	0.754923
30	C	2.441394	2.571966	-0.828930

31	C	2.241742	0.449248	2.848898
32	H	0.440993	1.513080	3.240927
33	C	4.461857	0.712601	-0.145936
34	C	3.312687	0.226006	1.980412
35	C	3.476031	2.363875	-1.678085
36	H	1.654336	3.264067	-1.119668
37	H	2.193876	-0.101033	3.790993
38	C	4.525998	1.435850	-1.366184
39	C	5.481056	-0.233662	0.176092
40	C	4.367737	-0.697323	2.298208
41	H	3.522210	2.899623	-2.628525
42	C	5.603522	1.218535	-2.231302
43	C	6.541647	-0.452657	-0.732233
44	C	5.397349	-0.913236	1.445849
45	H	4.325218	-1.219244	3.256513
46	C	6.586896	0.294399	-1.915548
47	H	5.654988	1.775671	-3.168968
48	H	6.190750	-1.605513	1.723611
49	H	7.406825	0.121917	-2.614906
50	H	-1.076881	3.046357	-1.216507
51	C	0.511810	5.054159	0.493195
52	H	-0.169771	5.860168	0.807793
53	H	1.499822	5.267184	0.932302
54	H	0.606383	5.099796	-0.600852
55	C	-0.876941	3.680273	2.828336
56	H	-1.614602	4.495625	2.764164
57	H	-1.399082	2.791539	3.215146
58	H	-0.109713	3.977143	3.561544
59	Cl	-0.606123	3.287665	-2.755786
60	C	-7.260875	-2.116691	-0.232713
61	C	-8.111526	-1.619843	-1.233598
62	C	-7.798230	-3.015963	0.690507
63	C	-9.441869	-2.000084	-1.297043
64	H	-7.717535	-0.937585	-1.988947
65	C	-9.136922	-3.402609	0.644197
66	H	-7.162193	-3.418965	1.481164
67	C	-9.966470	-2.891525	-0.354388
68	H	-10.096564	-1.618451	-2.082078
69	H	-9.516440	-4.098105	1.390675
70	C	7.618204	-1.452380	-0.484277
71	C	8.960053	-1.067676	-0.495719
72	C	7.332778	-2.813767	-0.291387
73	C	9.991620	-1.988865	-0.312076
74	H	9.215019	-0.015921	-0.641115
75	C	8.345566	-3.741983	-0.111711
76	H	6.295758	-3.154358	-0.299671
77	C	9.684347	-3.335267	-0.118386
78	H	11.023384	-1.641506	-0.320783
79	H	8.118048	-4.799902	0.028931
80	O	-11.282568	-3.200977	-0.492362
81	O	10.608605	-4.316564	0.067481
82	C	-11.858397	-4.101850	0.444397
83	H	-11.804744	-3.704211	1.470486
84	H	-12.910635	-4.209330	0.154322
85	H	-11.370144	-5.088710	0.406395
86	C	11.980954	-3.949398	0.053135
87	H	12.545220	-4.876651	0.210753
88	H	12.270582	-3.510199	-0.915050
89	H	12.217282	-3.240762	0.863126

Table S5. Optimized structural coordinates of **5**

Num.	Atom	Coordinates / Å		
		X	Y	Z
1	Si	-0.472710	2.867362	1.481518
2	C	-4.739380	-2.679955	-0.914611
3	C	-3.399829	-2.393919	-1.113823
4	C	-2.913469	-1.087616	-0.957135
5	C	-3.813044	-0.065936	-0.564571
6	C	-5.191201	-0.360383	-0.361972
7	C	-5.660498	-1.682352	-0.563118
8	C	-1.545736	-0.756373	-1.195343
9	C	-3.322281	1.260729	-0.379092
10	C	-1.948656	1.550022	-0.584632
11	C	-1.078662	0.513914	-1.021168
12	C	-1.455545	2.890643	-0.326455
13	C	-2.436041	3.931610	-0.119502
14	C	-3.755806	3.646898	0.071155
15	C	-4.221254	2.307312	-0.021842
16	C	-5.586317	1.983584	0.216960
17	C	-6.044561	0.707908	0.067498
18	H	-7.089887	0.491858	0.283294
19	H	-6.263758	2.778974	0.530320
20	H	-0.870442	-1.547032	-1.528507
21	H	-5.100069	-3.697203	-1.070752
22	H	-2.710915	-3.185200	-1.415792
23	H	-0.034124	0.750981	-1.219026
24	H	-2.082960	4.963194	-0.084788
25	H	-4.475627	4.440439	0.275184
26	C	0.863807	1.531839	1.446797
27	C	2.074511	1.607806	0.712353
28	C	0.661638	0.424346	2.284018
29	C	3.058131	0.581537	0.852579
30	C	2.357701	2.689256	-0.197542
31	C	1.612063	-0.576288	2.427933
32	H	-0.263102	0.337130	2.855555
33	C	4.290501	0.643206	0.121346
34	C	2.819476	-0.513162	1.727618
35	C	3.522790	2.750470	-0.886915
36	H	1.623750	3.478648	-0.360927
37	H	1.425248	-1.418312	3.097625
38	C	4.532313	1.740131	-0.746764
39	C	5.271437	-0.383944	0.263338
40	C	3.835396	-1.518905	1.878720
41	H	3.707395	3.580618	-1.571648
42	C	5.743803	1.803225	-1.443656
43	C	6.475090	-0.305078	-0.473430
44	C	4.999129	-1.455850	1.189368
45	H	3.650839	-2.337514	2.577493
46	C	6.690738	0.801117	-1.304644
47	H	5.931285	2.647222	-2.110486
48	H	5.756673	-2.223310	1.341227
49	H	7.620580	0.857350	-1.873201
50	H	-0.594289	3.217057	-0.935587
51	C	0.187354	4.605269	1.698193
52	H	-0.589215	5.252122	2.135028
53	H	1.044731	4.584915	2.390224
54	H	0.515765	5.053130	0.748473
55	C	-1.740599	2.465484	2.799899
56	H	-2.505400	3.254384	2.843275
57	H	-2.244867	1.499887	2.649368
58	H	-1.238065	2.437427	3.780710
59	Cl	0.442830	5.307516	-2.126102
60	C	-7.091774	-2.054801	-0.430502
61	C	-8.099360	-1.385692	-1.145297
62	C	-7.468731	-3.132053	0.375217
63	C	-9.424609	-1.773758	-1.045382
64	H	-7.836519	-0.562278	-1.811579
65	C	-8.799509	-3.527255	0.493516
66	H	-6.708316	-3.671576	0.943150
67	C	-9.786199	-2.844814	-0.219999
68	H	-10.202864	-1.259502	-1.611126
69	H	-9.050459	-4.363792	1.143194
70	C	7.531228	-1.353208	-0.401516

71	C	8.841773	-1.014961	-0.059459
72	C	7.267868	-2.694217	-0.724209
73	C	9.861286	-1.966309	-0.020585
74	H	9.078602	0.019476	0.197747
75	C	8.269855	-3.650634	-0.694215
76	H	6.259941	-2.990476	-1.020651
77	C	9.574987	-3.293826	-0.337737
78	H	10.866754	-1.657765	0.260497
79	H	8.061144	-4.689890	-0.953261
80	O	-11.108695	-3.147157	-0.180474
81	O	10.489914	-4.300853	-0.331040
82	C	-11.521543	-4.237614	0.633866
83	H	-11.279899	-4.061214	1.694012
84	H	-12.609898	-4.306484	0.518418
85	H	-11.061710	-5.182259	0.302885
86	C	11.828513	-3.980717	0.021779
87	H	12.391390	-4.920240	-0.037538
88	H	12.266567	-3.251997	-0.678945
89	H	11.891097	-3.585637	1.048496

Table S6. Optimized structural coordinates of **B**

Num.	Atom	Coordinates / Å		
		X	Y	Z
1	Si	-0.478946	2.886490	0.053134
2	C	-4.968258	-2.880138	0.092368
3	C	-3.595643	-2.791561	-0.060105
4	C	-2.999228	-1.607107	-0.517549
5	C	-3.824801	-0.490511	-0.798893
6	C	-5.237154	-0.582732	-0.643912
7	C	-5.814253	-1.803962	-0.212830
8	C	-1.590316	-1.497251	-0.711923
9	C	-3.224286	0.727796	-1.232997
10	C	-1.815387	0.817634	-1.377025
11	C	-1.016578	-0.331605	-1.131686
12	C	-1.218398	2.091625	-1.731623
13	C	-2.100711	3.141519	-2.185577
14	C	-3.453245	3.043932	-2.050747
15	C	-4.042214	1.852043	-1.543823
16	C	-5.446640	1.750977	-1.348257
17	C	-6.013987	0.592034	-0.904393
18	H	-7.089339	0.560383	-0.735545
19	H	-6.066320	2.626174	-1.547625
20	H	-0.969318	-2.373638	-0.515492
21	H	-5.411705	-3.819363	0.424422
22	H	-2.964606	-3.654422	0.161613
23	H	0.061178	-0.267859	-1.277469
24	H	-1.658349	4.050765	-2.594977
25	H	-4.104588	3.869312	-2.340278
26	C	0.987939	1.798698	0.529144
27	C	2.206716	1.701396	-0.189196
28	C	0.845889	1.048372	1.705822
29	C	3.241271	0.834423	0.275728
30	C	2.458678	2.450522	-1.395143
31	C	1.854708	0.220268	2.177086
32	H	-0.075513	1.115456	2.285179
33	C	4.471795	0.704502	-0.450225
34	C	3.055592	0.093181	1.474761
35	C	3.623729	2.339236	-2.078028
36	H	1.702670	3.142444	-1.761665
37	H	1.715002	-0.338274	3.104592
38	C	4.669792	1.463267	-1.633723
39	C	5.496715	-0.175067	0.011253
40	C	4.118338	-0.755704	1.940397
41	H	3.790176	2.925738	-2.983985
42	C	5.878008	1.342621	-2.328573
43	C	6.694219	-0.298980	-0.729221
44	C	5.274968	-0.883231	1.248330
45	H	3.973990	-1.293170	2.879769
46	C	6.864153	0.478103	-1.881847
47	H	6.033062	1.929692	-3.236096
48	H	6.067395	-1.521230	1.636726
49	H	7.790788	0.379585	-2.449758
50	H	-0.253514	2.022201	-2.249784
51	C	-0.039775	4.669028	-0.317205
52	H	0.618662	5.028690	0.490797
53	H	0.478582	4.827745	-1.272927
54	H	-0.950771	5.286687	-0.306392
55	C	-1.855133	2.793376	1.301798
56	H	-2.160105	1.767095	1.549503
57	H	-1.521587	3.294269	2.229076
58	H	-2.735323	3.342137	0.933966
59	Cl	-0.965839	4.717813	4.527173
60	C	-7.279862	-1.997321	-0.078227
61	C	-8.161353	-1.741181	-1.141648
62	C	-7.817230	-2.500442	1.109417
63	C	-9.520861	-1.969113	-1.013443
64	H	-7.771710	-1.381032	-2.095213
65	C	-9.184342	-2.723459	1.256577
66	H	-7.157020	-2.708118	1.953708
67	C	-10.044196	-2.456343	0.189860
68	H	-10.201548	-1.780438	-1.844890
69	H	-9.563402	-3.101502	2.204330
70	C	7.791071	-1.226002	-0.333978
71	C	9.088960	-0.747830	-0.144804
72	C	7.578548	-2.607047	-0.195368
73	C	10.145471	-1.597720	0.182449
74	H	9.286096	0.321748	-0.241917
75	C	8.618150	-3.465348	0.123899
76	H	6.580829	-3.019216	-0.357288
77	C	9.910728	-2.965798	0.318017
78	H	11.139402	-1.178980	0.330602
79	H	8.448830	-4.538846	0.222161
80	O	-11.387956	-2.640377	0.224211
81	O	10.865693	-3.882720	0.630370
82	C	-11.964873	-3.137993	1.425535
83	H	-11.787399	-2.453049	2.269689
84	H	-13.042983	-3.207150	1.237053
85	H	-11.575599	-4.138189	1.673496
86	C	12.193250	-3.418714	0.834142
87	H	12.791945	-4.306593	1.071277
88	H	12.597310	-2.941796	-0.073252
89	H	12.247587	-2.710522	1.676431

Table S7. Optimized structural coordinates of **TS$_{5\text{–}6}$**

Num.	Atom	Coordinates / Å		
		X	Y	Z
1	Si	-0.486649	3.327051	0.414406
2	C	-4.604870	-2.868220	-0.075834
3	C	-3.244940	-2.669906	-0.249701
4	C	-2.745489	-1.410372	-0.604630
5	C	-3.653734	-0.332742	-0.759682
6	C	-5.051826	-0.539466	-0.584215
7	C	-5.529624	-1.830864	-0.257006
8	C	-1.349250	-1.182601	-0.824315

9	C	-3.150933	0.962013	-1.093740
10	C	-1.755584	1.165876	-1.264215
11	C	-0.871618	0.049763	-1.145814
12	C	-1.271472	2.484422	-1.545771
13	C	-2.212910	3.511901	-1.841269
14	C	-3.561444	3.311519	-1.675767
15	C	-4.052505	2.048470	-1.279351
16	C	-5.447913	1.825925	-1.064534
17	C	-5.920058	0.597405	-0.723276
18	H	-6.986280	0.468853	-0.542520
19	H	-6.131481	2.669802	-1.169284
20	H	-0.665225	-2.028308	-0.728900
21	H	-4.974280	-3.863116	0.176362
22	H	-2.550107	-3.502955	-0.126083
23	H	0.193428	0.203620	-1.315627
24	H	-1.849064	4.484541	-2.172744
25	H	-4.268843	4.121588	-1.857804
26	C	0.868957	2.085150	0.780269
27	C	2.094503	1.969787	0.078963
28	C	0.612535	1.198492	1.836401
29	C	3.025897	0.950731	0.442547
30	C	2.450453	2.840606	-1.013392
31	C	1.522051	0.221973	2.211131
32	H	-0.320446	1.276442	2.395856
33	C	4.258936	0.795088	-0.273406
34	C	2.730881	0.076991	1.524326
35	C	3.620019	2.703837	-1.683983
36	H	1.772847	3.642423	-1.299529
37	H	1.298861	-0.442743	3.047814
38	C	4.564162	1.678906	-1.341586
39	C	5.177410	-0.238229	0.080290
40	C	3.691844	-0.926902	1.890363
41	H	3.868114	3.383016	-2.502221
42	C	5.774175	1.528974	-2.027254
43	C	6.375687	-0.387416	-0.654547
44	C	4.851388	-1.077008	1.207295
45	H	3.467443	-1.566928	2.746026
46	C	6.655858	0.515246	-1.687703
47	H	6.012602	2.212208	-2.844943
48	H	5.566276	-1.836618	1.520093
49	H	7.582999	0.397557	-2.251152
50	H	-0.266228	2.565355	-1.974165
51	C	-0.024904	5.036190	-0.179849
52	H	0.841858	5.381378	0.399437
53	H	0.212572	5.074808	-1.251906
54	H	-0.860475	5.723544	0.020092
55	C	-1.971237	3.181289	1.523560
56	H	-2.411588	2.174825	1.521850
57	H	-1.643371	3.435053	2.541936
58	H	-2.738783	3.908891	1.224015
59	Cl	0.559426	4.958655	3.247828
60	C	-6.976932	-2.135985	-0.105242
61	C	-7.896600	-1.883010	-1.136050
62	C	-7.453658	-2.740149	1.059973
63	C	-9.234556	-2.213028	-0.998661
64	H	-7.552982	-1.438486	-2.071766
65	C	-8.798973	-3.069565	1.216541
66	H	-6.762963	-2.948189	1.879472
67	C	-9.697241	-2.804989	0.182086
68	H	-9.943990	-2.024440	-1.805846
69	H	-9.130546	-3.528395	2.146288
70	C	7.355566	-1.474740	-0.378496
71	C	8.692269	-1.175943	-0.108811
72	C	6.989267	-2.829121	-0.437157
73	C	9.639910	-2.176330	0.108794
74	H	9.008055	-0.132203	-0.053107
75	C	7.919146	-3.834606	-0.228653
76	H	5.957222	-3.099992	-0.667546
77	C	9.252770	-3.514894	0.049017
78	H	10.669472	-1.896418	0.325537
79	H	7.630917	-4.885612	-0.283705
80	O	-11.024948	-3.087438	0.228056
81	O	10.091973	-4.568017	0.241826
82	C	-11.539376	-3.687514	1.410007
83	H	-11.394162	-3.036623	2.286849
84	H	-12.613587	-3.825080	1.236972
85	H	-11.074509	-4.668373	1.598127
86	C	11.456479	-4.289223	0.523190
87	H	11.947045	-5.263004	0.642275
88	H	11.935029	-3.742101	-0.304826
89	H	11.565746	-3.712875	1.455781

Table S8. Optimized structural coordinates of **6**

Num.	Atom	Coordinates / Å		
		X	Y	Z
1	C	-0.432984	2.265821	0.132493
2	C	0.850401	2.792595	0.137144
3	C	1.966150	1.951233	0.090655
4	C	1.762154	0.546276	0.016547
5	C	0.441021	0.009440	0.002355
6	C	-0.665347	0.886480	0.080817
7	C	3.310764	2.466586	0.114355
8	C	2.896781	-0.325053	-0.048989
9	C	4.214675	0.211333	-0.021784
10	C	4.381894	1.639497	0.064216
11	C	5.309204	-0.660055	-0.081523
12	C	5.119239	-2.035414	-0.170780
13	C	3.834979	-2.568156	-0.204304
14	C	2.713911	-1.731013	-0.144979
15	C	1.370747	-2.243753	-0.192135
16	C	0.295991	-1.421323	-0.124429
17	H	-0.706899	-1.842315	-0.179404
18	H	1.232011	-3.322303	-0.293748
19	H	3.445927	3.548631	0.175423
20	H	-1.289174	2.940058	0.193421
21	H	0.998109	3.873237	0.190525
22	H	5.397309	2.041034	0.084693
23	H	5.983908	-2.700247	-0.216919
24	H	3.687696	-3.647654	-0.278788
25	H	6.319076	-0.245023	-0.058167
26	C	-2.073506	0.400884	0.111281
27	C	-2.529678	-0.480790	1.104624
28	C	-3.002803	0.857065	-0.825265
29	C	-3.852487	-0.890196	1.150008
30	H	-1.836301	-0.840602	1.867028
31	C	-4.337417	0.451421	-0.796203
32	H	-2.679082	1.541192	-1.612351
33	C	-4.766737	-0.428706	0.196595
34	H	-4.202806	-1.569506	1.928832
35	H	-5.024673	0.827035	-1.552424
36	O	-6.041859	-0.887626	0.319343
37	C	-7.003526	-0.441954	-0.626583
38	H	-6.730721	-0.742290	-1.651088
39	H	-7.948502	-0.925262	-0.349807
40	H	-7.131559	0.651719	-0.586674

Table S9. Optimized structural coordinates of **7**

Num.	Atom	Coordinates / Å		
		X	Y	Z
1	Si	5.395434	-0.260257	0.200547
2	C	3.588355	-0.775267	0.022957
3	C	2.486139	0.112922	0.090412
4	C	3.332565	-2.143778	-0.141757
5	C	1.155047	-0.395091	0.011833
6	C	2.651649	1.538740	0.229823
7	C	2.041601	-2.646655	-0.230552
8	H	4.163642	-2.848114	-0.203683
9	C	0.027578	0.487847	0.094759
10	C	0.940221	-1.790862	-0.152530
11	C	1.589577	2.377000	0.294890
12	H	3.656895	1.955617	0.269350
13	H	1.877397	-3.718091	-0.362253
14	C	0.242345	1.884465	0.232797
15	C	-1.302230	-0.026576	0.034954
16	C	-0.408155	-2.280681	-0.248318
17	H	1.742749	3.453622	0.394114
18	C	-0.862001	2.740524	0.298860
19	C	-2.398093	0.860461	0.135507
20	C	-1.470562	-1.446056	-0.160468
21	H	-0.558182	-3.351137	-0.403172
22	C	-2.151014	2.233851	0.252129
23	H	-0.698327	3.815304	0.400791
24	H	-2.479042	-1.846587	-0.252128
25	H	-2.999691	2.915591	0.329614
26	C	5.777369	0.659548	1.782507
27	H	5.192117	1.582589	1.897220
28	H	5.555023	0.006246	2.642907
29	H	6.847230	0.919144	1.817708
30	C	6.546817	-1.714339	-0.016209
31	H	6.406748	-2.450005	0.792350
32	H	6.398895	-2.220746	-0.981745
33	H	7.588039	-1.358619	0.028390
34	Cl	5.872793	1.076551	-1.406675
35	C	-3.812675	0.393506	0.122355
36	C	-4.719923	0.905538	-0.806984
37	C	-4.296962	-0.525187	1.067564
38	C	-6.060170	0.518609	-0.817192
39	H	-4.373669	1.619731	-1.556880
40	C	-5.626010	-0.916391	1.073875
41	H	-3.621194	-0.928932	1.823770
42	C	-6.517967	-0.398877	0.128090
43	H	-6.729491	0.937573	-1.566638
44	H	-5.998517	-1.624521	1.815843
45	O	-7.801125	-0.843575	0.209795
46	C	-8.741385	-0.338164	-0.727841
47	H	-9.698064	-0.817627	-0.487099
48	H	-8.851825	0.754516	-0.638610
49	H	-8.457978	-0.595252	-1.761152

Table S10. Optimized structural coordinates of **4***.

Num.	Atom	Coordinates / Å		
		X	Y	Z
1	Si	-0.051901	-4.353798	0.101900
2	C	5.174464	1.294716	2.221723
3	C	4.079190	0.635370	2.704163
4	C	3.388373	-0.338335	1.900669
5	C	3.907141	-0.659766	0.610964
6	C	5.072230	0.013882	0.114441
7	C	5.672890	1.084181	0.908555
8	C	2.238503	-0.990226	2.357264
9	C	3.274327	-1.678013	-0.160967
10	C	2.092726	-2.335008	0.319623
11	C	1.597920	-1.950108	1.583255
12	C	1.456004	-3.358666	-0.474898
13	C	2.032529	-3.676303	-1.717964
14	C	3.176753	-3.064147	-2.189412
15	C	3.831918	-2.054891	-1.419972
16	C	5.015392	-1.437827	-1.849150
17	C	5.626123	-0.440275	-1.093957
18	H	6.565954	-0.022478	-1.449252
19	H	5.465149	-1.751329	-2.793512
20	H	1.840529	-0.734912	3.341495
21	H	5.634194	2.069350	2.836518
22	H	3.689579	0.862781	3.698226
23	H	0.690611	-2.411785	1.971288
24	H	1.568710	-4.444245	-2.340723
25	H	3.601028	-3.348113	-3.154893
26	C	-1.527502	-3.253842	0.616410
27	C	-2.115552	-2.278004	-0.226010
28	C	-2.102133	-3.463330	1.877912
29	C	-3.260618	-1.544511	0.208259
30	C	-1.595429	-1.988381	-1.539845
31	C	-3.216353	-2.757184	2.313094
32	H	-1.670602	-4.206202	2.551126
33	C	-3.865637	-0.558300	-0.640028
34	C	-3.813189	-1.796099	1.493202
35	C	-2.169961	-1.066414	-2.349015
36	H	-0.708472	-2.520432	-1.882960
37	H	-3.638954	-2.951898	3.301284
38	C	-3.325999	-0.322625	-1.932510
39	C	-5.004584	0.179586	-0.196834
40	C	-4.980344	-1.064229	1.903949
41	H	-1.756294	-0.867089	-3.339875
42	C	-3.933232	0.627068	-2.761071
43	C	-5.576722	1.154542	-1.045818
44	C	-5.546756	-0.129769	1.104058
45	H	-5.411467	-1.287211	2.882342
46	C	-5.033826	1.346002	-2.322127
47	H	-3.522045	0.807277	-3.756473
48	H	-6.440693	0.395097	1.437565
49	H	-5.478670	2.099232	-2.974950
50	C	-0.611169	-5.503439	-1.284279
51	H	-0.892365	-4.968796	-2.203938
52	H	0.168685	-6.239809	-1.536698
53	H	-1.497630	-6.063050	-0.943266
54	C	0.438374	-5.433547	1.570735
55	H	1.288196	-6.069108	1.272853
56	H	0.753891	-4.850469	2.449000
57	H	-0.384981	-6.100178	1.874540
58	C	6.711551	1.963747	0.407000
59	C	6.760725	2.407250	-0.942245
60	C	7.703360	2.483878	1.273042
61	C	7.725739	3.288299	-1.382017
62	H	5.988069	2.090313	-1.641749
63	C	8.687971	3.355892	0.836098
64	H	7.726867	2.160268	2.314291
65	C	8.709145	3.766043	-0.502550
66	H	7.732442	3.638683	-2.415468
67	H	9.441632	3.703657	1.540778
68	C	-6.739262	1.992309	-0.637916
69	C	-6.694956	2.816102	0.488953
70	C	-7.906324	2.018346	-1.417332
71	C	-7.767793	3.633597	0.843919
72	H	-5.794062	2.838117	1.105076
73	C	-8.982943	2.821704	-1.076221
74	H	-7.974347	1.384700	-2.303928

75	C	-8.921350	3.635803	0.059412
76	H	-7.685780	4.265082	1.726936
77	H	-9.890922	2.830414	-1.681422
78	O	9.623492	4.611959	-1.030868
79	O	-10.024382	4.390254	0.316518
80	C	10.634421	5.132198	-0.174727
81	H	11.261919	4.326780	0.238364
82	H	11.252150	5.787202	-0.800602
83	H	10.197962	5.720288	0.647748
84	C	-10.003900	5.230188	1.462107
85	H	-10.976508	5.736739	1.484907
86	H	-9.878708	4.646068	2.388003
87	H	-9.204745	5.985759	1.393949

Table S11. Optimized structural coordinates of **A***

Num.	Atom	Coordinates / Å		
		X	Y	Z
1	Si	-0.144808	4.169899	-0.362031
2	C	5.137778	-1.448449	-2.435043
3	C	3.997711	-0.849098	-2.893273
4	C	3.312782	0.137855	-2.104450
5	C	3.877281	0.535165	-0.855475
6	C	5.088406	-0.073215	-0.387464
7	C	5.689057	-1.156995	-1.160294
8	C	2.122559	0.731841	-2.538021
9	C	3.243165	1.563789	-0.099155
10	C	2.021246	2.161271	-0.556399
11	C	1.487095	1.706266	-1.778367
12	C	1.385338	3.206039	0.216418
13	C	1.997374	3.593775	1.416405
14	C	3.179682	3.028945	1.877493
15	C	3.844585	2.014376	1.113455
16	C	5.070830	1.464069	1.508541
17	C	5.683788	0.456155	0.769581
18	H	6.655578	0.090988	1.095158
19	H	5.552979	1.837217	2.414429
20	H	1.690395	0.419578	-3.490622
21	H	5.594863	-2.236415	-3.034472
22	H	3.569545	-1.137479	-3.855012
23	H	0.550026	2.123526	-2.145424
24	H	1.541004	4.381558	2.019758
25	H	3.665127	3.416120	2.776845
26	C	-1.610873	3.055359	-0.869512
27	C	-2.193330	2.078447	-0.024642
28	C	-2.183192	3.254779	-2.133886
29	C	-3.335508	1.339061	-0.456891
30	C	-1.667493	1.789803	1.287003
31	C	-3.286648	2.533762	-2.571836
32	H	-1.755347	3.997572	-2.809364
33	C	-3.941581	0.359000	0.397617
34	C	-3.879624	1.573145	-1.748743
35	C	-2.229080	0.856987	2.092984
36	H	-0.783607	2.327649	1.629878
37	H	-3.701545	2.713862	-3.566030
38	C	-3.384219	0.109886	1.679883
39	C	-5.093293	-0.366304	-0.033196
40	C	-5.016560	0.803348	-2.174818
41	H	-1.806811	0.653618	3.079241
42	C	-3.971948	-0.858032	2.501382
43	C	-5.682959	-1.315570	0.832532
44	C	-5.591508	-0.116285	-1.364007
45	H	-5.406281	0.971752	-3.181084
46	C	-5.095999	-1.551619	2.081677
47	H	-3.541900	-1.054544	3.485640
48	H	-6.441853	-0.694458	-1.722412
49	H	-5.553266	-2.286478	2.746676
50	H	2.167901	1.846822	3.230144
51	C	-0.712224	5.324363	1.016121
52	H	0.054987	6.078222	1.254891
53	H	-1.611842	5.863433	0.676567
54	H	-0.975829	4.793361	1.943193
55	C	0.342267	5.234978	-1.842089
56	H	1.187534	5.879400	-1.550575
57	H	0.664013	4.639940	-2.710296
58	H	-0.483725	5.892195	-2.158683
59	Cl	1.699372	1.063970	4.192071
60	C	6.784156	-1.972372	-0.669527
61	C	6.907067	-2.352367	0.693950
62	C	7.757949	-2.489754	-1.556718
63	C	7.925486	-3.173117	1.128078
64	H	6.151641	-2.035029	1.411785
65	C	8.796899	-3.299039	-1.126689
66	H	7.723257	-2.212669	-2.610939
67	C	8.891278	-3.647482	0.226746
68	H	7.990653	-3.477227	2.173933
69	H	9.534406	-3.647291	-1.847918
70	C	-6.909253	-2.080609	0.471248
71	C	-8.108037	-1.442567	0.144983
72	C	-6.907515	-3.483832	0.505541
73	C	-9.267261	-2.161614	-0.145863
74	H	-8.151463	-0.351850	0.131047
75	C	-8.049609	-4.213345	0.215574
76	H	-5.985904	-4.013908	0.754236
77	C	-9.239626	-3.555969	-0.112849
78	H	-10.181551	-1.622501	-0.388401
79	H	-8.038864	-5.304343	0.236141
80	O	9.861697	-4.428632	0.749201
81	O	-10.310395	-4.352069	-0.380534
82	C	10.866263	-4.933981	-0.124628
83	H	11.434733	-4.115622	-0.593471
84	H	11.538940	-5.530360	0.502977
85	H	10.429995	-5.577083	-0.904819
86	C	-11.541542	-3.722769	-0.706459
87	H	-12.261302	-4.532799	-0.876549
88	H	-11.900521	-3.087822	0.119421
89	H	-11.455339	-3.117880	-1.623503

Table S12. Optimized structural coordinates of **TS_{4-5}***

Num.	Atom	Coordinates / Å		
		X	Y	Z
1	Si	-0.007256	3.538531	-0.887019
2	C	4.983069	-2.707859	-0.631862
3	C	3.641104	-2.466287	-0.718856
4	C	3.116759	-1.145625	-0.524545
5	C	4.021773	-0.068198	-0.294067
6	C	5.431553	-0.318507	-0.219833
7	C	5.914874	-1.684253	-0.317089
8	C	1.742154	-0.886669	-0.566484
9	C	3.512426	1.256634	-0.161685
10	C	2.106039	1.505362	-0.216168
11	C	1.252166	0.401901	-0.398615
12	C	1.579606	2.861388	-0.037234
13	C	2.556565	3.900592	0.135395
14	C	3.902270	3.666968	0.176079

15	C	4.425549	2.339501	0.018809
16	C	5.799667	2.081230	0.017240
17	C	6.296138	0.789560	-0.114131
18	H	7.373027	0.644405	-0.156485
19	H	6.495335	2.916676	0.119461
20	H	1.048807	-1.713918	-0.728331
21	H	5.344387	-3.731881	-0.728146
22	H	2.942271	-3.283352	-0.907158
23	H	0.175549	0.559853	-0.409421
24	H	2.208840	4.929737	0.257842
25	H	4.604430	4.491271	0.318482
26	C	-1.428110	2.297355	-1.191808
27	C	-2.362707	1.859260	-0.219854
28	C	-1.590037	1.835379	-2.506885
29	C	-3.435837	0.991367	-0.587897
30	C	-2.267422	2.250148	1.163749
31	C	-2.619600	0.979813	-2.874804
32	H	-0.889102	2.150301	-3.280535
33	C	-4.393354	0.553499	0.386780
34	C	-3.556665	0.549079	-1.932781
35	C	-3.159568	1.826899	2.091048
36	H	-1.445134	2.888841	1.478291
37	H	-2.706860	0.639608	-3.909001
38	C	-4.255164	0.969006	1.737672
39	C	-5.474624	-0.299417	0.009962
40	C	-4.631957	-0.339184	-2.280865
41	H	-3.055239	2.132486	3.134264
42	C	-5.183679	0.525381	2.685398
43	C	-6.414646	-0.705011	0.984712
44	C	-5.540923	-0.742120	-1.361668
45	H	-4.695816	-0.692207	-3.312384
46	C	-6.238095	-0.292104	2.311040
47	H	-5.075855	0.840214	3.725395
48	H	-6.335269	-1.427515	-1.653350
49	H	-6.965125	-0.608624	3.061010
50	H	1.160029	2.793051	1.480444
51	C	-0.609717	5.051762	0.068702
52	H	0.036939	5.917699	-0.144664
53	H	-1.629129	5.314888	-0.257566
54	H	-0.629904	4.910189	1.159027
55	C	0.565760	4.147796	-2.582689
56	H	1.327395	4.931172	-2.442294
57	H	1.024144	3.348251	-3.185347
58	H	-0.266498	4.584498	-3.158630
59	Cl	0.949468	2.682422	2.928415
60	C	7.297231	-2.061246	-0.067319
61	C	8.080676	-1.465301	0.954397
62	C	7.900358	-3.107884	-0.800735
63	C	9.368340	-1.882626	1.211867
64	H	7.643896	-0.695830	1.589960
65	C	9.202113	-3.519606	-0.566029
66	H	7.344589	-3.586069	-1.608242
67	C	9.949930	-2.907132	0.448088
68	H	9.950933	-1.435555	2.018682
69	H	9.630098	-4.310782	-1.179217
70	C	-7.591463	-1.559430	0.661648
71	C	-8.542971	-1.178775	-0.287152
72	C	-7.808476	-2.760678	1.354681
73	C	-9.668791	-1.958323	-0.552600
74	H	-8.418135	-0.238705	-0.827856
75	C	-8.919581	-3.548785	1.100224
76	H	-7.082613	-3.088799	2.101372
77	C	-9.859093	-3.152761	0.142730
78	H	-10.388007	-1.616869	-1.295253
79	H	-9.077925	-4.485760	1.636647
80	O	11.219273	-3.234733	0.763880
81	O	-10.917608	-3.988580	-0.038250
82	C	11.852829	-4.282218	0.034160
83	H	11.931602	-4.031250	-1.034925
84	H	12.858624	-4.378474	0.459569
85	H	11.312833	-5.233979	0.154975
86	C	-11.898065	-3.623831	-0.999326
87	H	-12.648080	-4.424194	-0.987057
88	H	-12.383502	-2.669760	-0.738015
89	H	-11.464618	-3.550014	-2.009768

Table S13. Optimized structural coordinates of **5***

Num.	Atom	Coordinates / Å		
		X	Y	Z
1	Si	-0.339256	3.050008	1.494744
2	C	-4.723126	-2.703588	-0.658164
3	C	-3.409507	-2.403993	-0.881776
4	C	-2.934206	-1.060990	-0.765518
5	C	-3.842023	-0.039796	-0.357230
6	C	-5.218174	-0.344967	-0.128661
7	C	-5.683034	-1.701164	-0.347699
8	C	-1.594826	-0.727179	-1.036945
9	C	-3.356266	1.288973	-0.172369
10	C	-1.995575	1.591598	-0.411406
11	C	-1.141871	0.568025	-0.869429
12	C	-1.477097	2.956297	-0.171438
13	C	-2.494002	3.985890	0.034439
14	C	-3.798140	3.679707	0.271279
15	C	-4.263418	2.322503	0.223376
16	C	-5.594337	1.982349	0.507557
17	C	-6.057982	0.686224	0.364269
18	H	-7.083697	0.463165	0.646627
19	H	-6.277293	2.764009	0.846169
20	H	-0.911599	-1.507565	-1.375217
21	H	-5.064584	-3.727825	-0.805956
22	H	-2.707592	-3.184147	-1.180408
23	H	-0.100321	0.807477	-1.085230
24	H	-2.172357	5.028932	0.034883
25	H	-4.524419	4.470415	0.469974
26	C	0.988718	1.699229	1.484740
27	C	2.134157	1.685910	0.647268
28	C	0.837597	0.658821	2.414762
29	C	3.112654	0.654833	0.789375
30	C	2.347932	2.675269	-0.377506
31	C	1.775079	-0.354213	2.551639
32	H	-0.037669	0.636696	3.065054
33	C	4.283676	0.636740	-0.038049
34	C	2.923591	-0.371728	1.753984
35	C	3.445279	2.649556	-1.173687
36	H	1.606674	3.455474	-0.549708
37	H	1.625581	-1.145181	3.289720
38	C	4.453047	1.639323	-1.028798
39	C	5.269990	-0.382894	0.121329
40	C	3.910072	-1.409181	1.869727
41	H	3.573201	3.406245	-1.950142
42	C	5.590720	1.606722	-1.843564
43	C	6.425172	-0.369136	-0.694317
44	C	5.022839	-1.415404	1.096551
45	H	3.738645	-2.205988	2.596560
46	C	6.552237	0.623754	-1.673974
47	H	5.719080	2.374320	-2.609385
48	H	5.744186	-2.224829	1.197900
49	H	7.444177	0.630664	-2.302885
50	H	-0.719682	3.281922	-0.914079
51	C	0.367148	4.790508	1.538915
52	H	-0.374576	5.487874	1.958807
53	H	1.259460	4.815015	2.185301
54	H	0.653383	5.157221	0.542108

55	C	-1.478131	2.781572	2.964439
56	H	-2.224050	3.589423	3.005508
57	H	-2.015738	1.822117	2.926825
58	H	-0.899833	2.812765	3.902231
59	Cl	0.271788	4.970235	-2.595604
60	C	-7.079567	-2.079997	-0.309434
61	C	-8.116805	-1.244650	-0.811950
62	C	-7.462509	-3.360236	0.169050
63	C	-9.425913	-1.659232	-0.828496
64	H	-7.867690	-0.279148	-1.249608
65	C	-8.778060	-3.774804	0.186247
66	H	-6.703297	-4.026143	0.580041
67	C	-9.777902	-2.924903	-0.318802
68	H	-10.211195	-1.027660	-1.245538
69	H	-9.027337	-4.752065	0.594787
70	C	7.518548	-1.370987	-0.557071
71	C	8.202958	-1.557273	0.646225
72	C	7.934450	-2.124951	-1.665838
73	C	9.258067	-2.462389	0.758285
74	H	7.922334	-0.969585	1.522300
75	C	8.977961	-3.031620	-1.570116
76	H	7.419409	-2.004783	-2.621032
77	C	9.647935	-3.207413	-0.354926
78	H	9.768663	-2.569374	1.713851
79	H	9.290492	-3.620677	-2.433887
80	O	-11.078197	-3.232855	-0.361777
81	O	10.659606	-4.117201	-0.354280
82	C	-11.505728	-4.509350	0.121250
83	H	-11.279560	-4.618288	1.192225
84	H	-12.590669	-4.534783	-0.029516
85	H	-11.034940	-5.322909	-0.450329
86	C	11.368595	-4.322192	0.859658
87	H	12.127305	-5.084205	0.643464
88	H	11.868426	-3.399372	1.195264
89	H	10.703669	-4.690300	1.657431

Table S14. Optimized structural coordinates of HCl

Num.	Atom	Coordinates / Å		
		X	Y	Z
1	Cl	0.000000	0.000000	0.072145
2	H	0.000000	0.000000	-1.226469

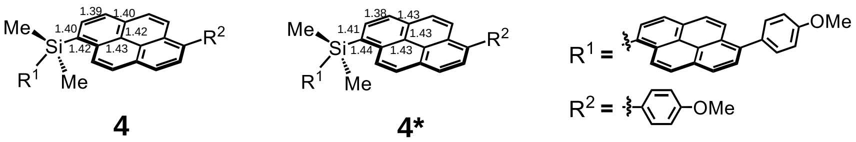

4 4*

Figure S46. Chemical structures and C–C bond length (Å) on the aromatic rings of **4** and **4***.

S10. Absorption spectra

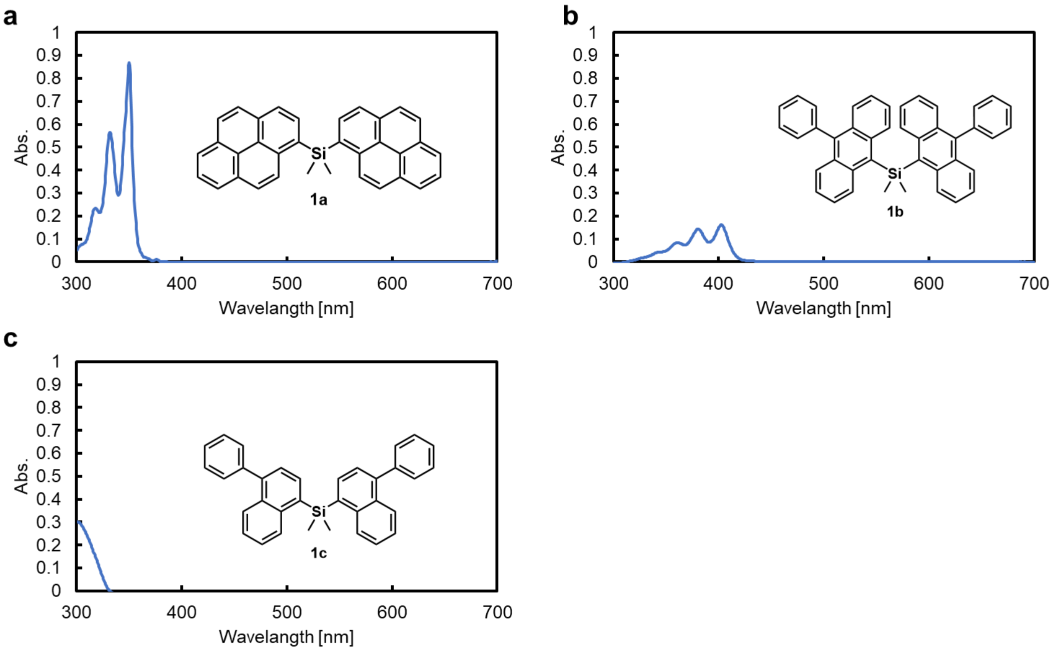

Figure S47. Absorption spectra in THF (1×10^{-5} M) of (**a**) **1a**, (**b**) **1b**, and (**c**) **1c**.

Absorption spectra of **2** and **8** in EtOAc (1×10^{-5} M) were measured. Both UV-vis spectra of **2** and **8** showed a similar absorption band at the wavelength of around 365 nm. Similarly, absorption spectra of **1** and **2** in 0.2 M HCl solution in EtOAc (1×10^{-5} M) were measured. In both **2** and **8**, the spectra were not changed by HCl.

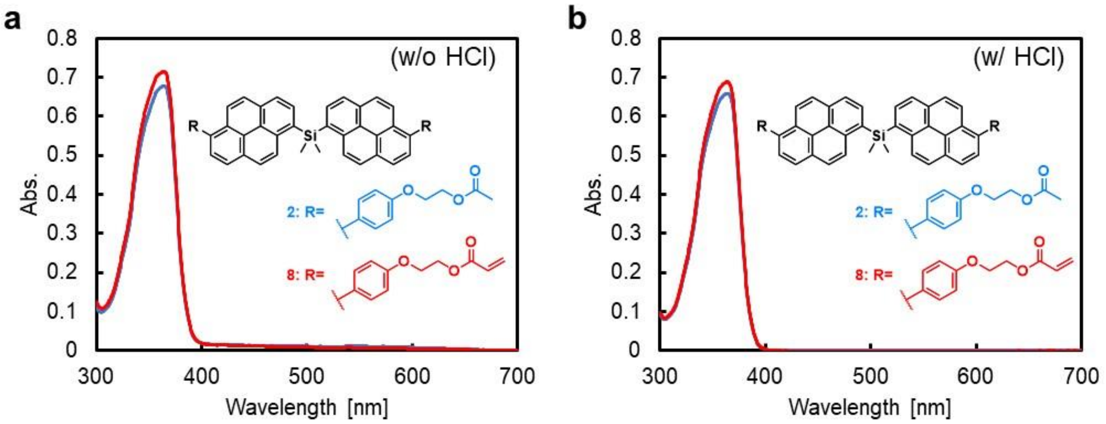

Figure S48. Absorption spectra of **2** and **8** in (**a**) EtOAc, and (**b**) 0.2 M HCl solution in EtOAc (1×10^{-5} M).

S11. NMR spectra

S11.1 ^{1}H NMR, $^{13}C\{^{1}H\}$ NMR, and $^{29}Si\{^{1}H\}$ NMR spectra

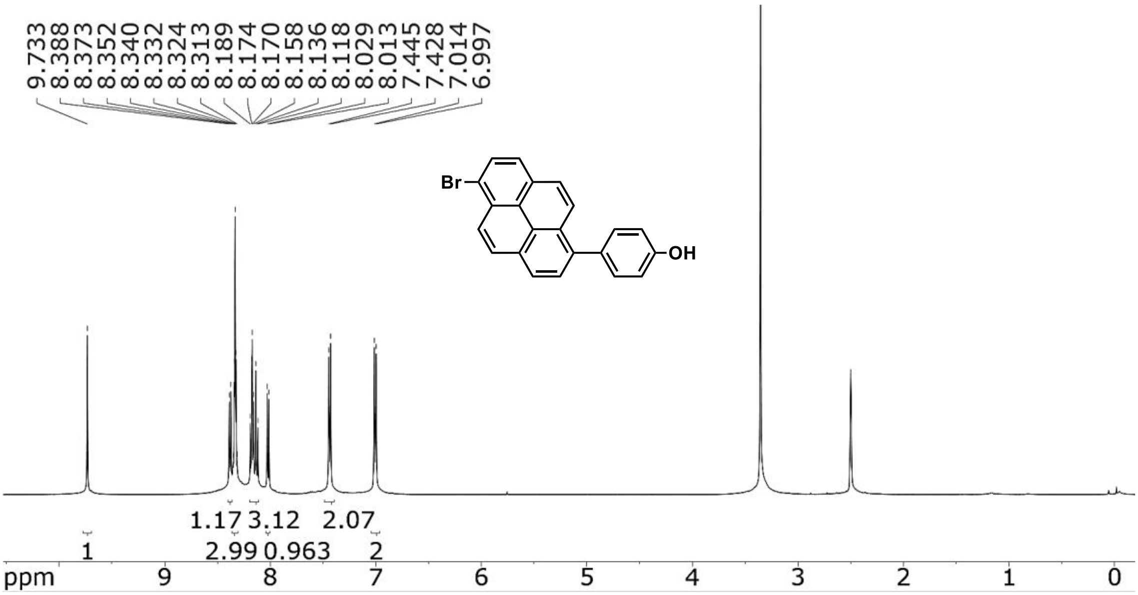

Figure S49. ^{1}H NMR spectrum of **S1** (500 MHz, DMSO-d_6, r.t.).

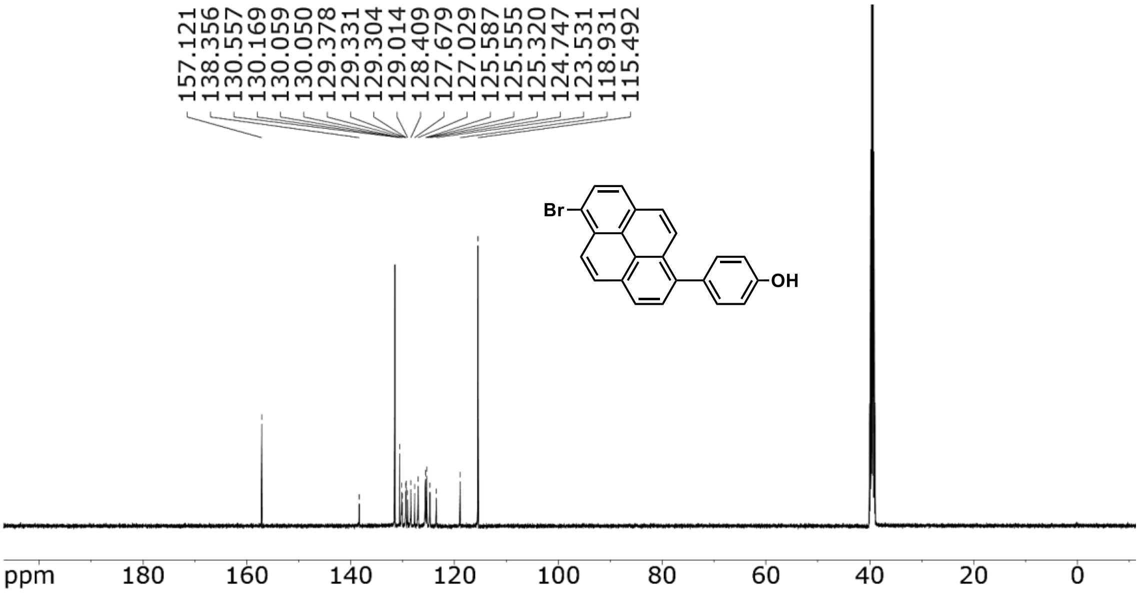

Figure S50. $^{13}C\{^{1}H\}$ NMR spectrum of **S1** (126 MHz, DMSO-d_6, r.t.).

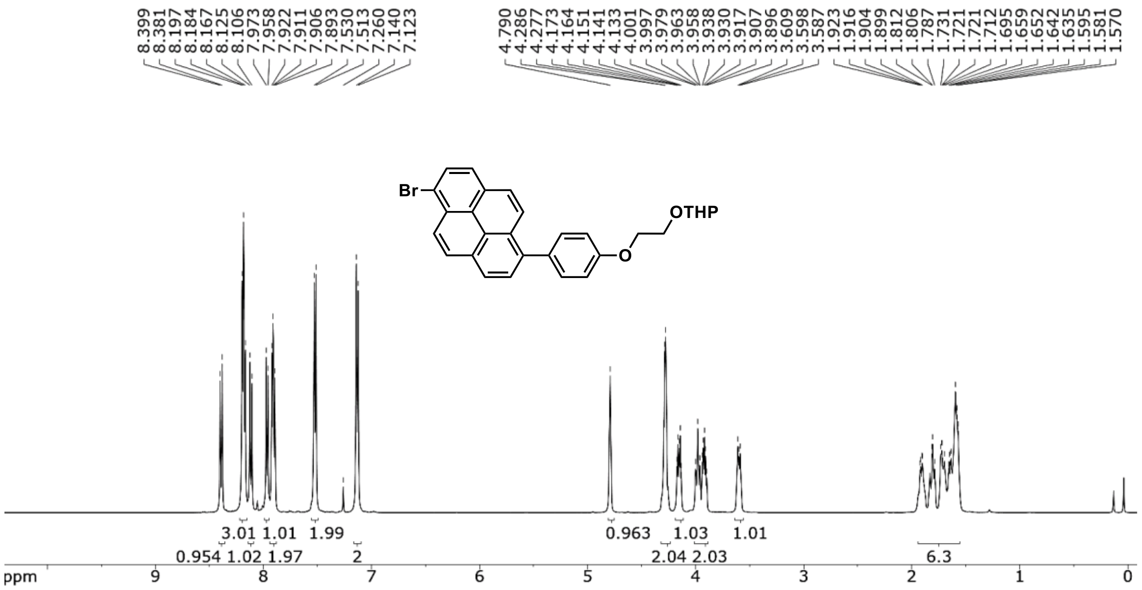

Figure S51. ^{1}H NMR spectrum of **S2** (500 MHz, $CDCl_3$, r.t.).

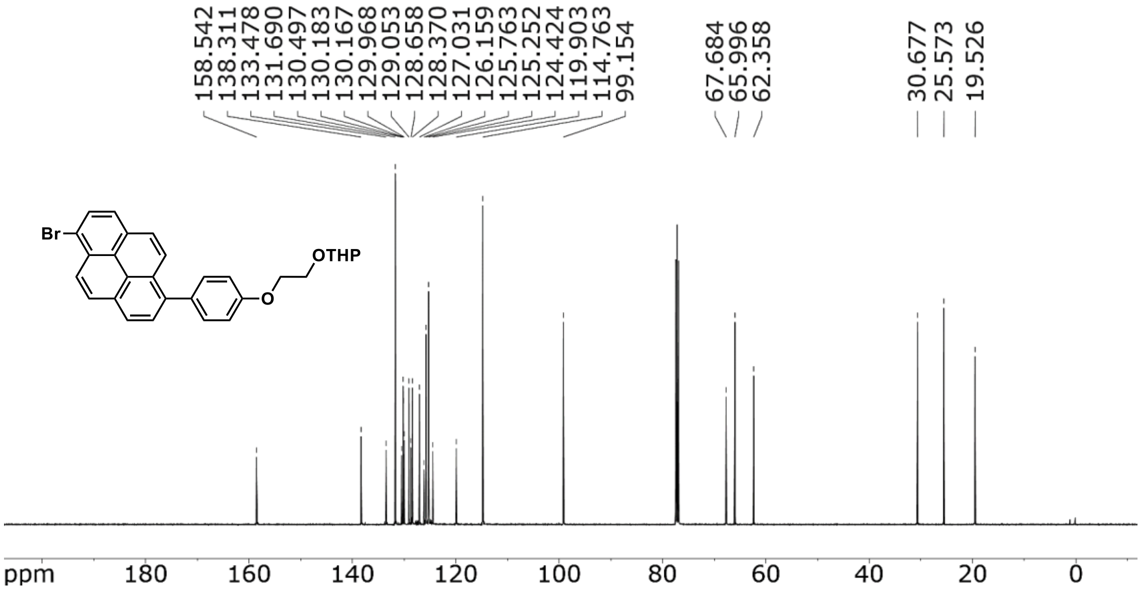

Figure S52. $^{13}C\{^{1}H\}$ NMR spectrum of **S2** (126 MHz, $CDCl_3$, r.t.).

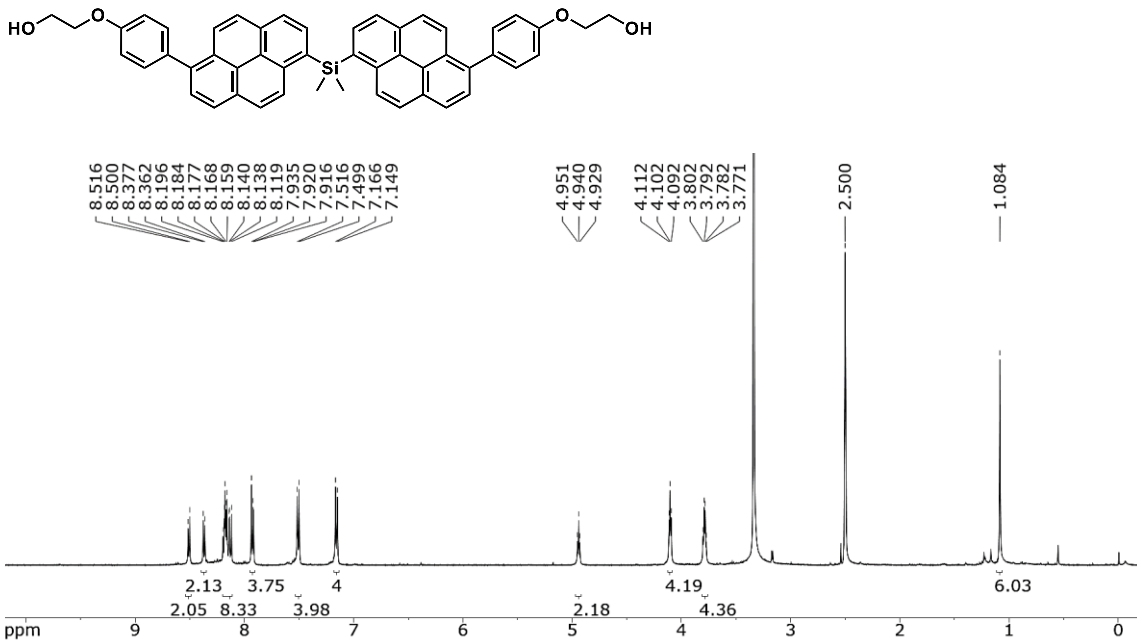

Figure S53. ^{1}H NMR spectrum of **S3** (500 MHz, DMSO-d_6, r.t.).

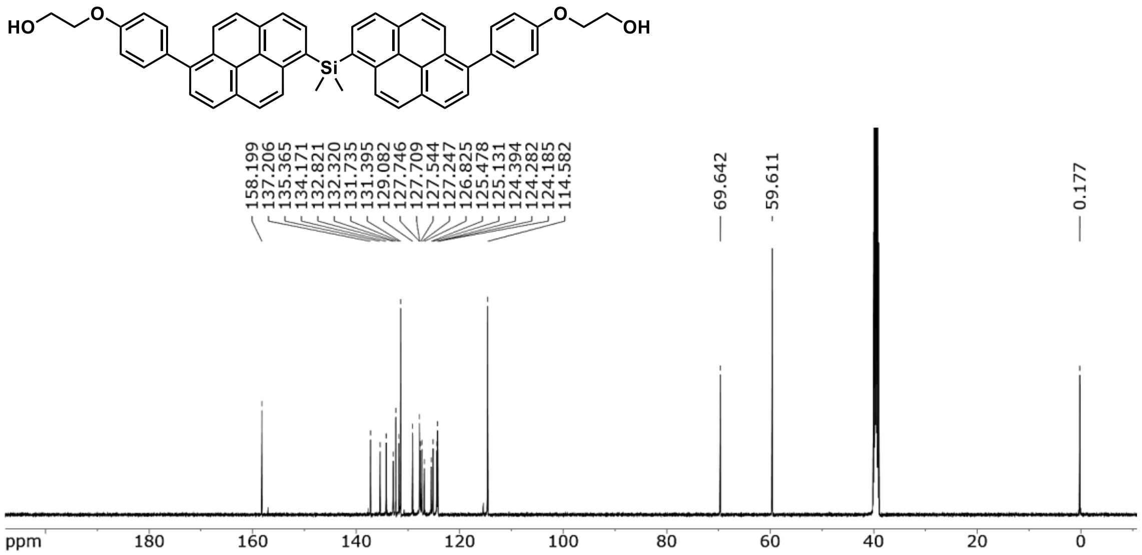

Figure S54. $^{13}C\{^{1}H\}$ NMR spectrum of **S3** (126 MHz, DMSO-d_6, r.t.).

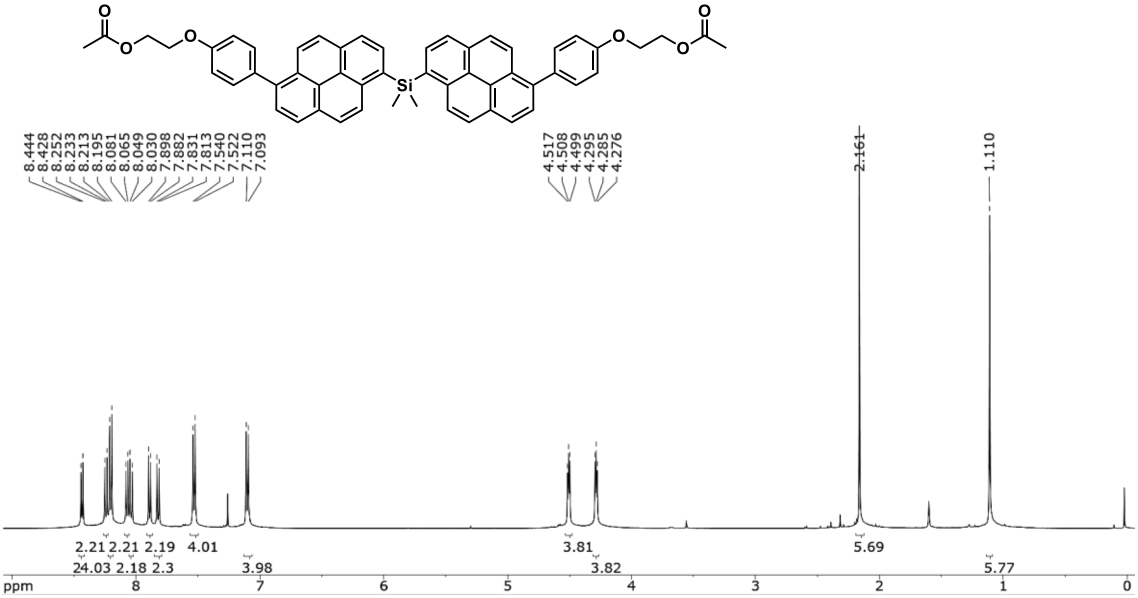

Figure S55. ^{1}H NMR spectrum of **2** (500 MHz, $CDCl_3$, r.t.).

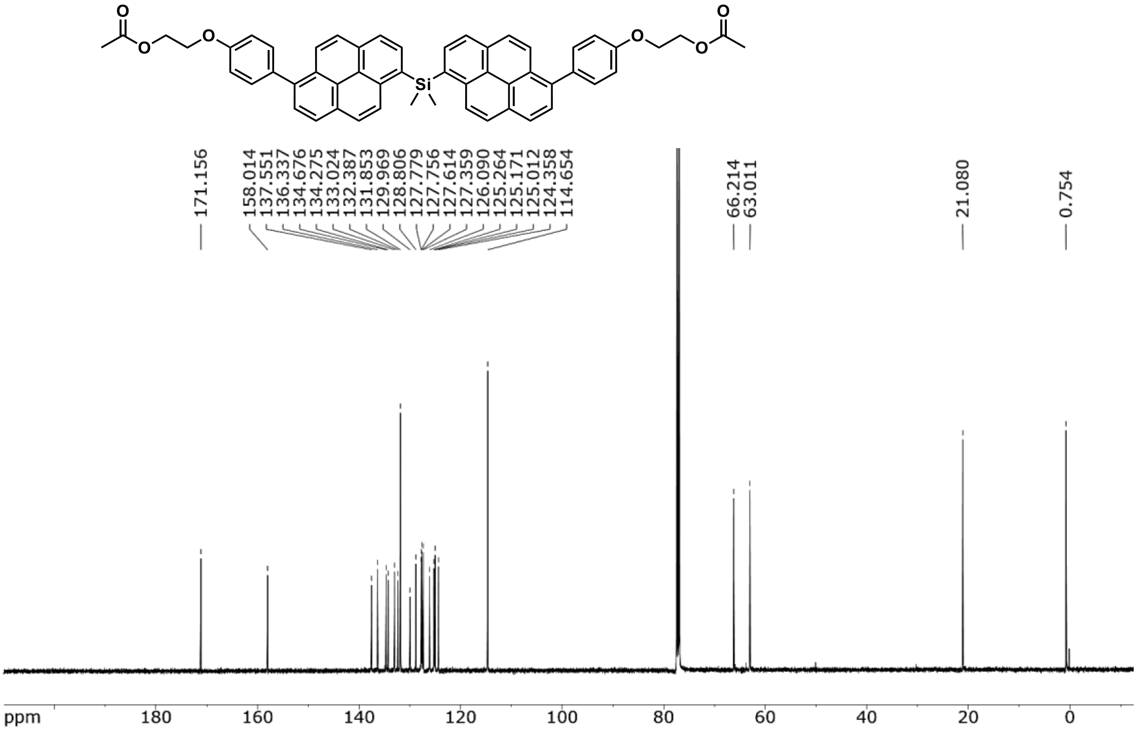

Figure S56. $^{13}C\{^{1}H\}$ NMR spectrum of **2** (126 MHz, $CDCl_3$, r.t.).

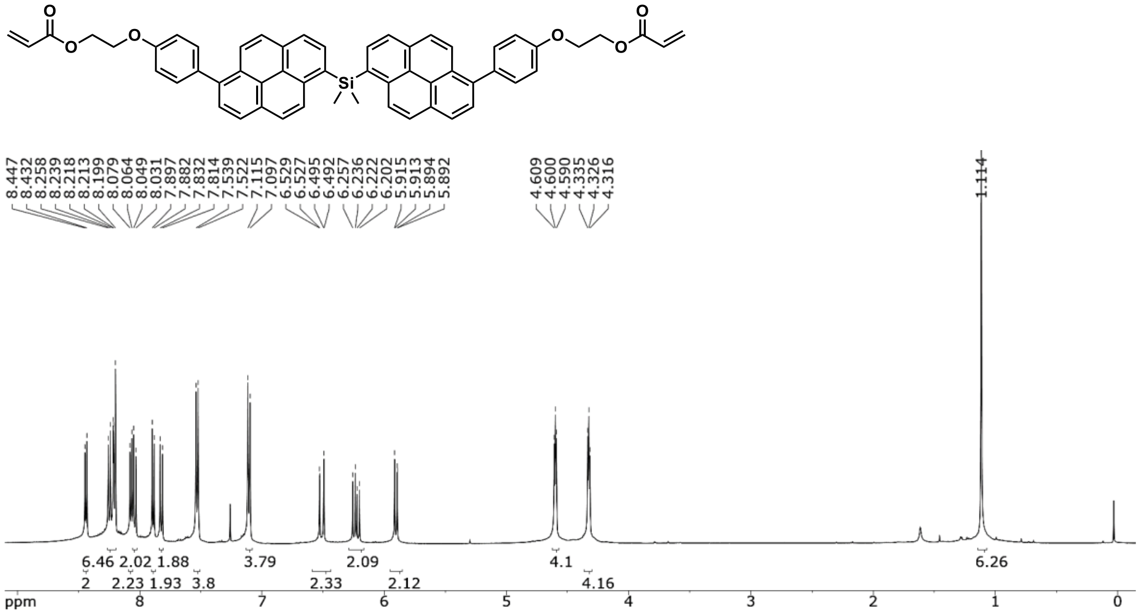

Figure S57. ^{1}H NMR spectrum of **8** (500 MHz, $CDCl_3$, r.t.).

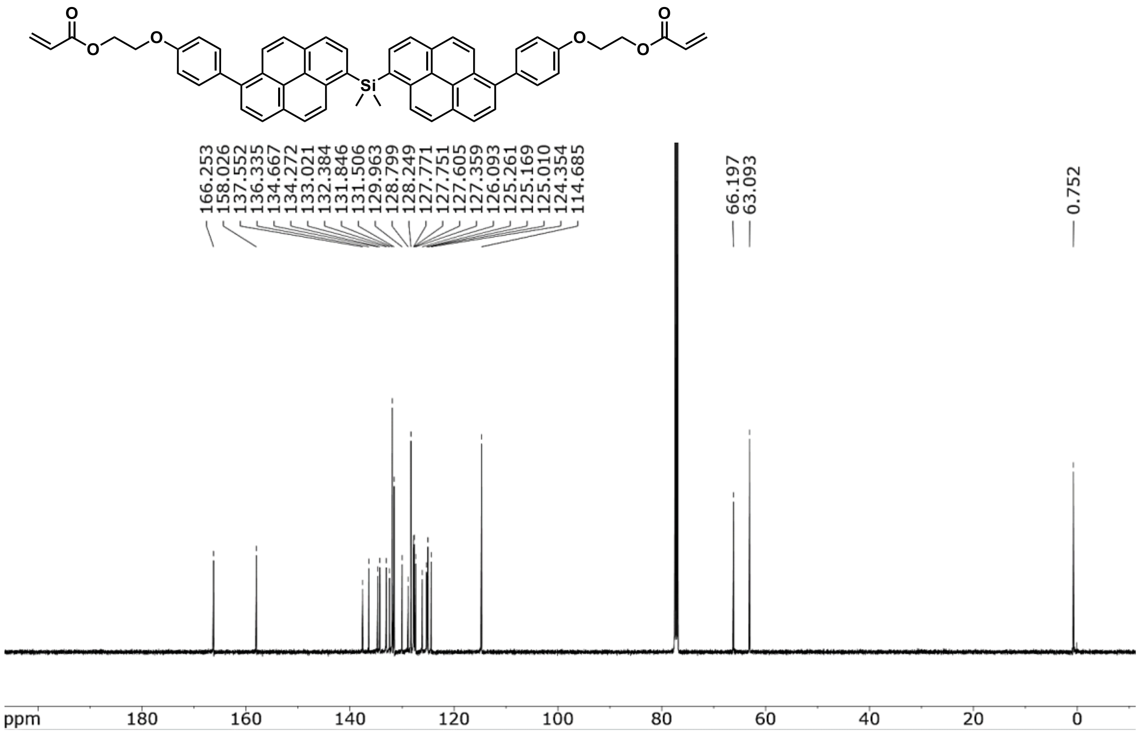

Figure S58. $^{13}C\{^1H\}$ NMR spectrum of **8** (126 MHz, $CDCl_3$, r.t.).

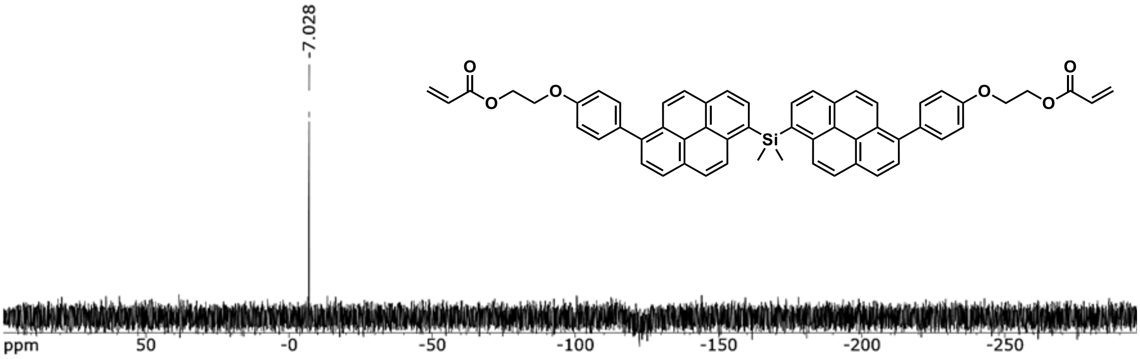

Figure S59. $^{29}Si\{^{1}H\}$ NMR spectrum of **8** (99 MHz; $CDCl_3$, r.t.).

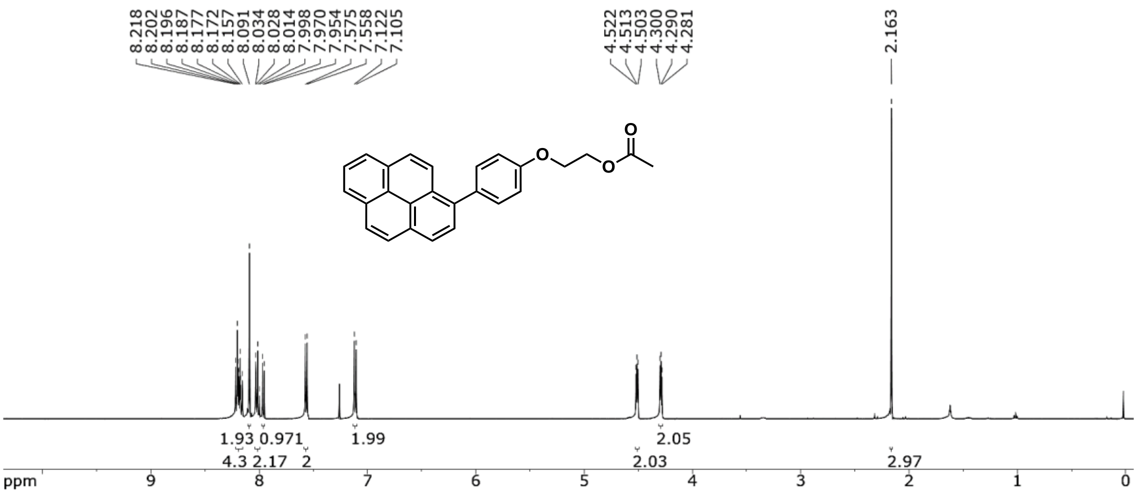

Figure S60. ^{1}H NMR spectrum of **3** (500 MHz, $CDCl_3$, r.t.).

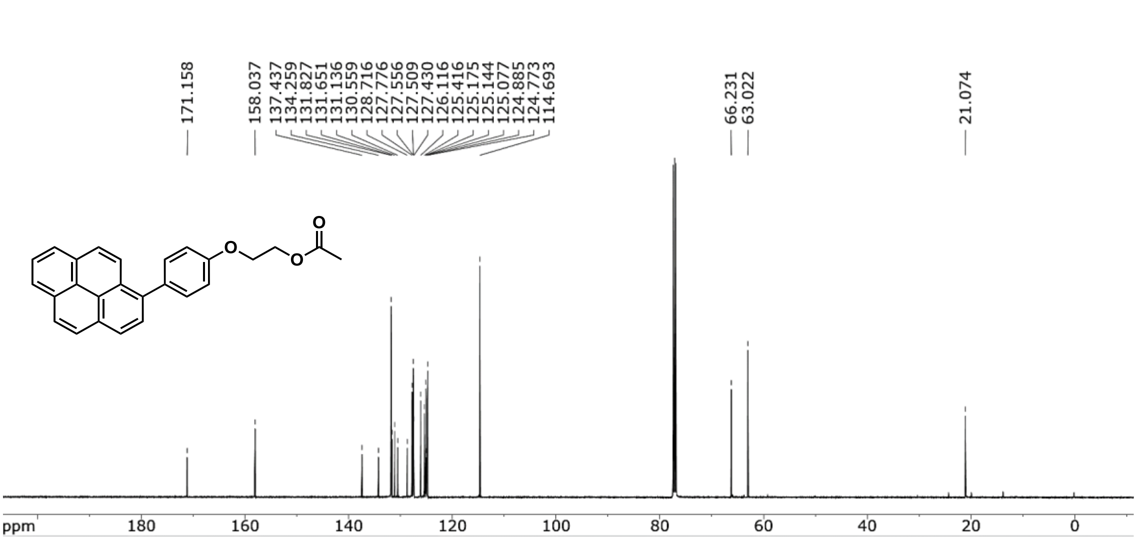

Figure S61. $^{13}C\{^{1}H\}$ NMR spectrum of **3** (126 MHz, $CDCl_3$, r.t.).

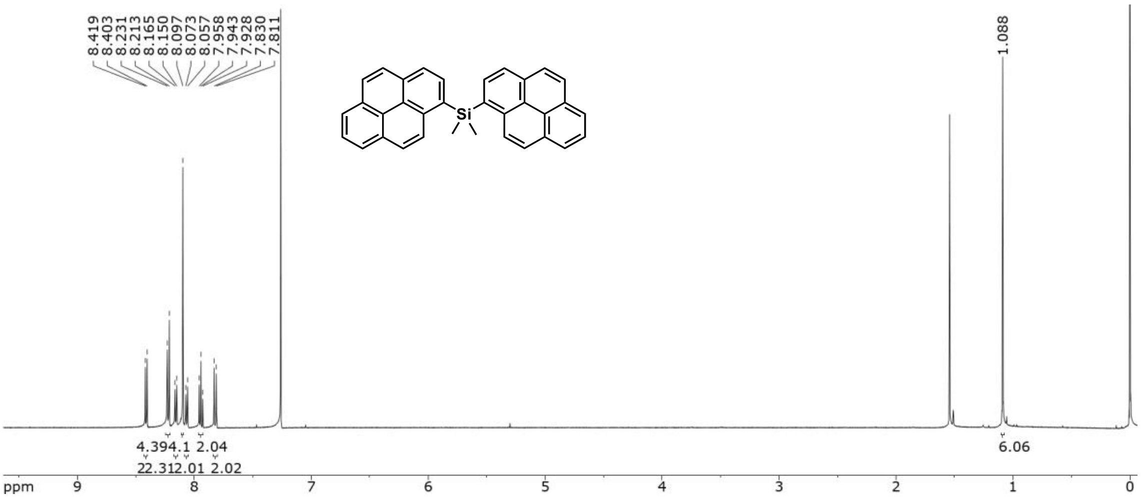

Figure S62. ^{1}H NMR spectrum of **1a** (500 MHz, $CDCl_3$, r.t.).

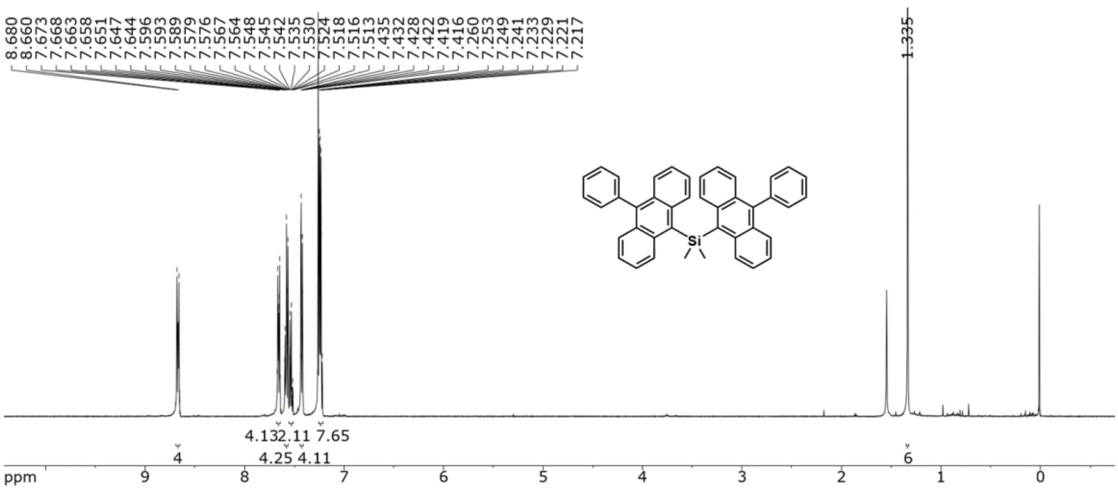

Figure S63. ^{1}H NMR spectrum of **1b** (500 MHz, $CDCl_3$, r.t.).

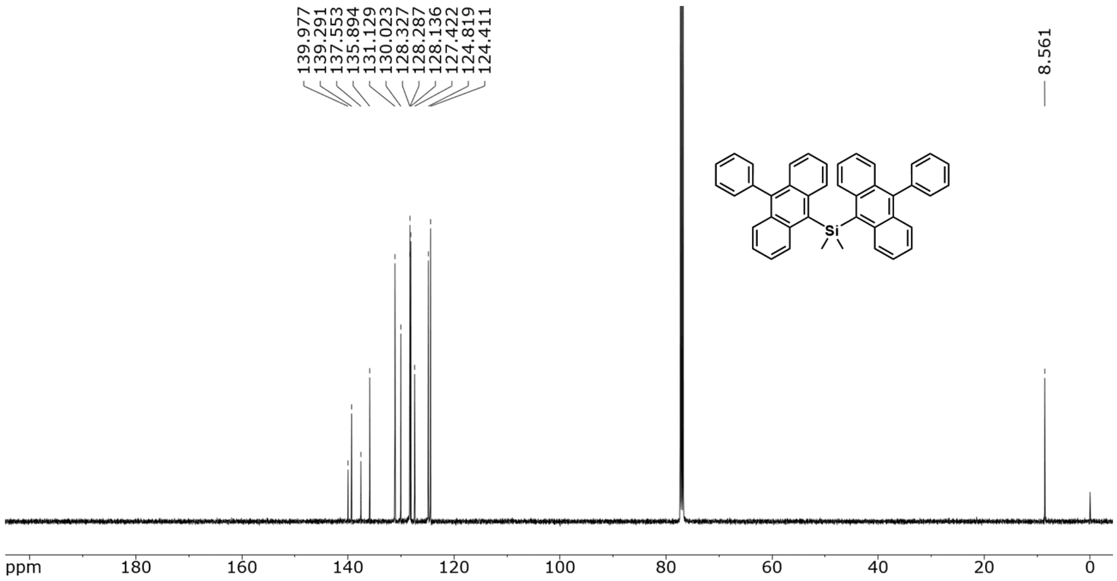

Figure S64. $^{13}C\{^{1}H\}$ NMR spectrum of **1b** (126 MHz, $CDCl_3$, r.t.).

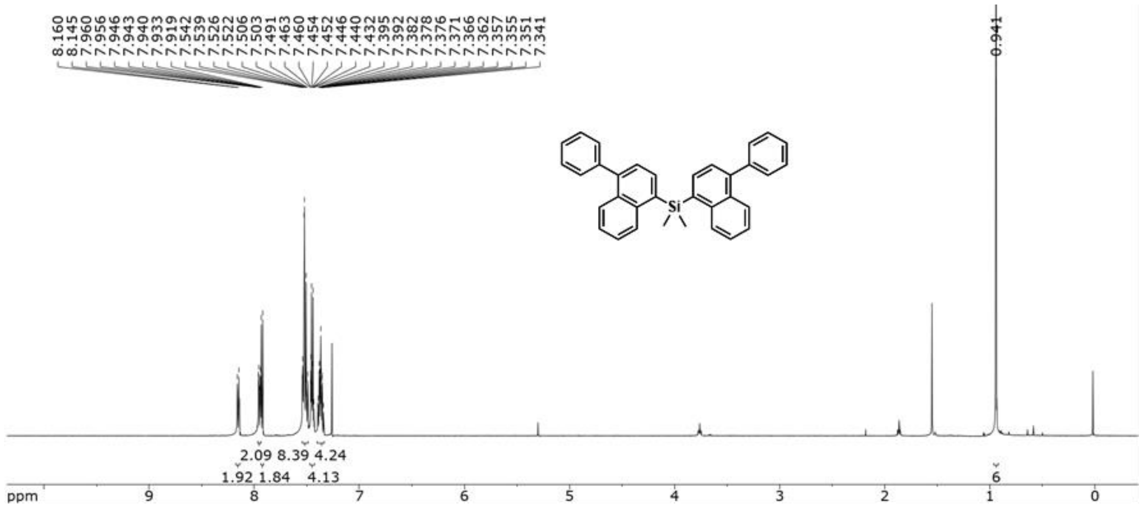

Figure S65. ^{1}H NMR spectrum of **1c** (500 MHz, $CDCl_3$, r.t.).

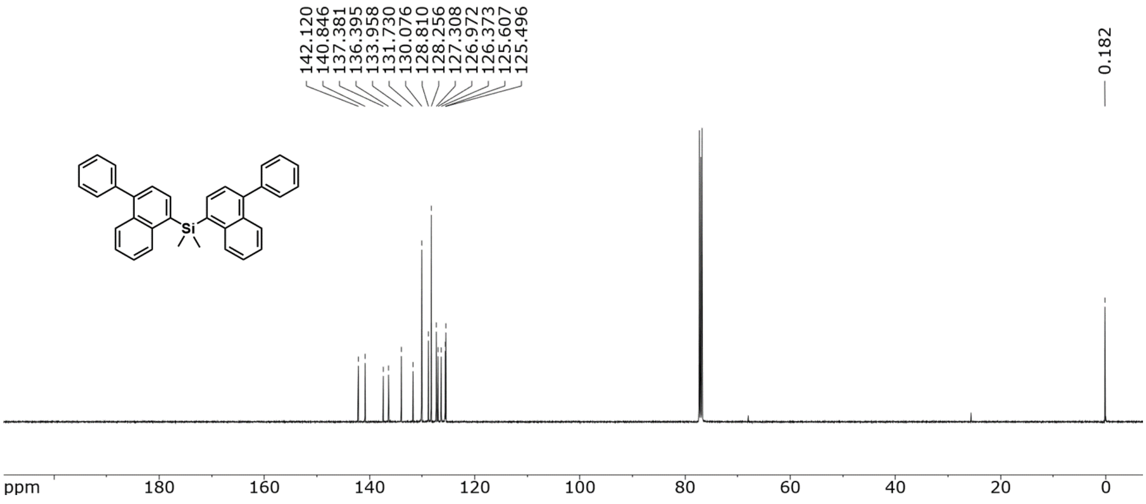

Figure S66. $^{13}C\{^1H\}$ NMR spectrum of **1c** (126 MHz, $CDCl_3$, r.t.).

S11.2 2D NMR spectra

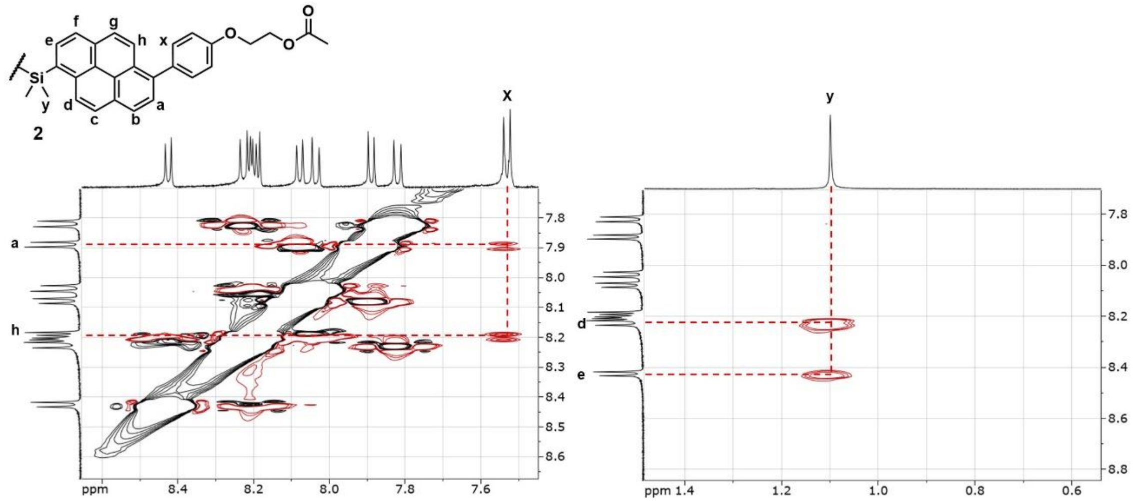

Figure S67. Selected region of NOESY NMR spectra of **2** (500 MHz, $CDCl_3$, r.t.).

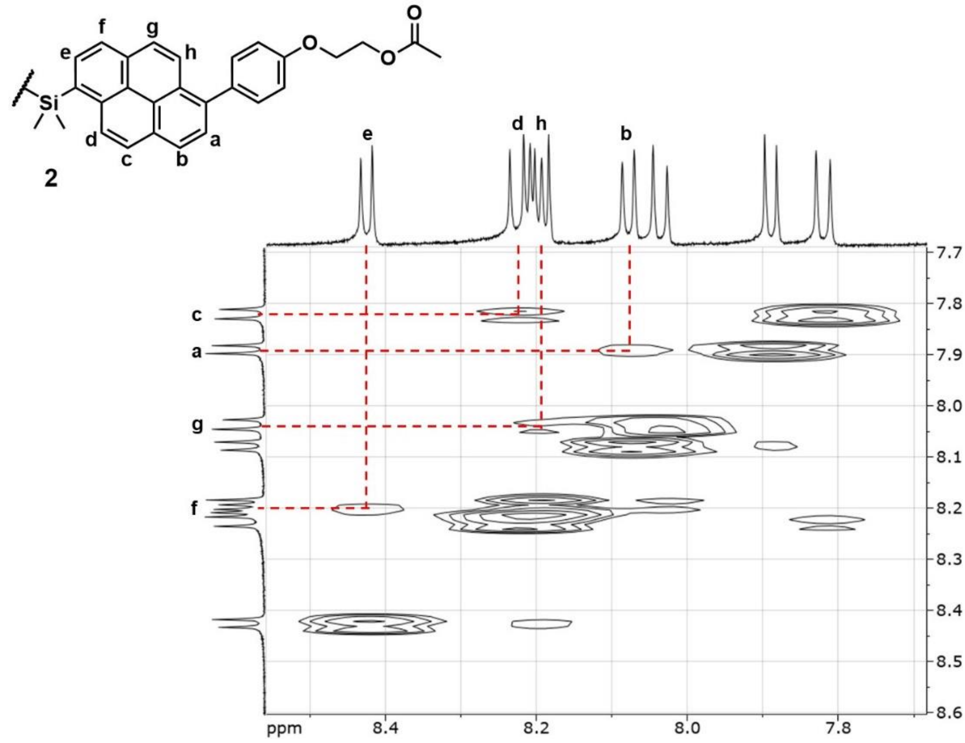

Figure S68. Selected region of COSY NMR spectrum of **2** (500 MHz, $CDCl_3$, r.t.).

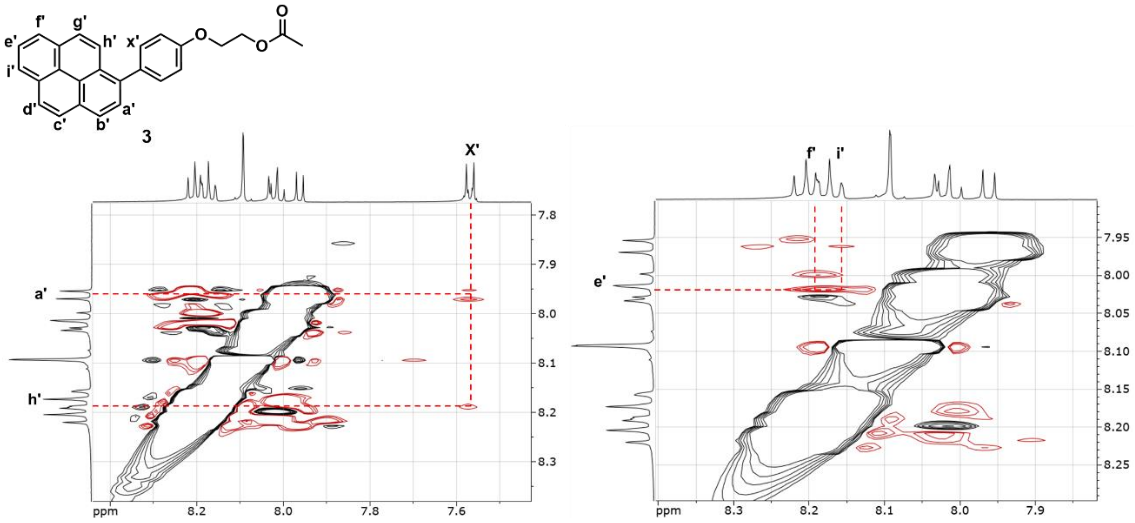

Figure S69. Selected region of NOESY NMR spectra of **3** (500 MHz, $CDCl_3$, r.t.).

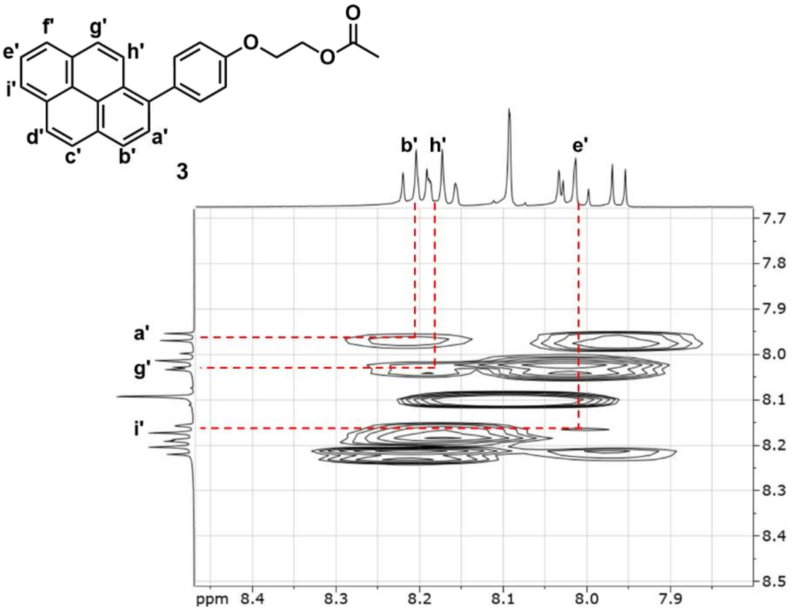

Figure S70. Selected region of COSY NMR spectrum of **3** (500 MHz, $CDCl_3$, r.t.).

S12. Reference

[1] A. B. Pangborn, M. A. Giardello, R. H. Grubbs, R. K. Rosen, F. J. Timmers, *Organometallics* **1996**, *15*, 1518.

[2] G. M. Russell, T. Kaneko, S. Ishino, H. Masai, J. Terao, *Adv. Funct. Mater.* **2022**, *32*, 202205855.

[3] S. I. Kondo, Y. Taguchi, Y. Bie, *RSC Adv.* **2015**, *5*, 5846.

[4] J. F. G. A. Jansen, A. A. Dias, M. Dorschu, B. Coussens, *Macromolecules* **2003**, *36*, 3861.

[5] M. J. Frisch, G. W. Trucks, H. B. Schlegel, G. E. Scuseria, M. A. Robb, J. R. Cheeseman, G. Scalmani, V. Barone, G. A. Petersson, H. Nakatsuji, X. Li, M. Caricato, A. V. Marenich, J. Bloino, B. G. Janesko, R. Gomperts, B. Mennucci, H. P. Hratchian, J. V. Ortiz, A. F. Izmaylov, J. L. Sonnenberg, D. Williams-Young, F. Ding, F. Lipparini, F. Egidi, J. Goings, B. Peng, A. Petrone, T. Henderson, D. Ranasinghe, V. G. Zakrzewski, J. Gao, N. Rega, G. Zheng, W. Liang, M. Hada, M. Ehara, K. Toyota, R. Fukuda, J. Hasegawa, M. Ishida, T. Nakajima, Y. Honda, O. Kitao, H. Nakai, T. Vreven, K. Throssell, J. A. Montgomery, Jr., J. E. Peralta, F. Ogliaro, M. J. Bearpark, J. J. Heyd, E. N. Brothers, K. N. Kudin, V. N. Staroverov, T. A. Keith, R. Kobayashi, J. Normand, K. Raghavachari, A. P. Rendell, J. C. Burant, S. S. Iyengar, J. Tomasi, M. Cossi, J. M. Millam, M. Klene, C. Adamo, R. Cammi, J. W. Ochterski, R. L. Martin, K. Morokuma, O. Farkas, J. B. Foresman, and D. J. Fox Gaussian 16, Revision B.01; Gaussian, Inc., Wallingford CT (2016).

[6] A. V. Marenich, C. J. Cramer, D. G. Truhlar, *J. Phys. Chem. B* **2009**, *113*, 6378.
